# Proteomic analysis of *Biomphalaria glabrata* plasma proteins with binding affinity to those expressed by early developing larval *Schistosoma mansoni*

**DOI:** 10.1371/journal.ppat.1006081

**Published:** 2017-05-16

**Authors:** Xiao-Jun Wu, Nathalie Dinguirard, Grzegorz Sabat, Hong-di Lui, Laura Gonzalez, Michael Gehring, Utibe Bickham-Wright, Timothy P. Yoshino

**Affiliations:** 1 Department of Pathobiological Sciences, University of Wisconsin, Madison, WI, United States of America; 2 Biotechnology Center, Mass Spectrometry/Proteomics Facility, University of Wisconsin, Madison, WI, United States of America; George Washington University School of Medicine and Health Sciences, UNITED STATES

## Abstract

Interactions between early developing *Schistosoma mansoni* larval stages and the hemolymph of its snail intermediate host represent the first molecular encounter with the snail’s immune system. To gain a more comprehensive understanding of this early parasite-host interaction, biotinylated sporocyst tegumental membrane (Mem) proteins and larval transformation proteins (LTP) were affixed to streptavidin-agarose beads and used as affinity matrices to enrich for larval-reactive plasma proteins from susceptible (NMRI) and resistant (BS-90) strains of the snail *Biomphalaria glabrata*. Nano-LC/MS-MS proteomic analyses of isolated plasma proteins revealed a diverse array of 94 immune-and nonimmune-related plasma proteins. Included among the immune-related subset were pattern recognition receptors (lectins, LPS-binding protein, thioester-containing proteins-TEPs), stress proteins (HSP60 and 70), adhesion proteins (dermatopontins), metalloproteases (A Disintegrin And Metalloproteinase (ADAM), ADAM-related Zn proteinases), cytotoxins (biomphalysin) and a Ca^2+^-binding protein (neo-calmodulin). Variable immunoglobulin and lectin domain (VIgL) gene family members, including fibrinogen-related proteins (FREPs), galectin-related proteins (GREPs) and C-type lectin-related proteins (CREPs), were the most prevalent of larval-reactive immune lectins present in plasma. FREPs were highly represented, although only a subset of FREP subfamilies (FREP 2, 3 and 12) were identified, suggesting potential selectivity in the repertoire of plasma lectins recognizing larval glycoconjugates. Other larval-binding FREP-like and CREP-like proteins possessing a C-terminal fibrinogen-related domain (FReD) or C-type lectin binding domain, respectively, and an Ig-fold domain also were identified as predicted proteins from the *B*. *glabrata* genome, although incomplete sequence data precluded their placement into specific FREP/CREP subfamilies. Similarly, a group of FReD-containing proteins (angiopoeitin-4, ficolin-2) that lacked N-terminal Ig-fold(s) were identified as a distinct group of FREP-like proteins, separate from the VIgL lectin family. Finally, differential appearance of GREPs in BS-90 plasma eluates, and others proteins exclusively found in eluates of the NMRI strain, suggested snail strain differences in the expression of select larval-reactive immune proteins. This hypothesis was supported by the finding that differential gene expression of the GREP in BS-90 and ADAM in NMRI snail strains generally correlated with their patterns of protein expression. In summary, this study is the first to provide a global comparative proteomic analysis of constitutively expressed plasma proteins from susceptible and resistant *B*. *glabrata* strains capable of binding early-expressed larval *S*. *mansoni* proteins. Identified proteins, especially those exhibiting differential expression, may play a role in determining immune compatibility in this snail host-parasite system. A complete listing of raw peptide data are available via ProteomeXchange using identifier PXD004942.

## Author summary

Transmission of the human blood fluke *Schistosoma mansoni* critically depends on the successful establishment of infections within species of its snail intermediate host, *Biomphalaria*. One of the most important barriers to infection is the host’s innate immune system, comprised of plasma proteins and immunocytes (hemocytes) circulating in the hemolymph. Although expression of plasma lectin genes appears to be associated with larval resistance in *B*. *glabrata*, few studies have attempted an in depth analysis of gene-encoded lectins, and other immune proteins, that are capable of directly binding schistosome larvae. Using affinity matrices linked to schistosome proteins expressed during early larval development, we identified and compared the parasite-reactive plasma proteins from the susceptible NMRI and resistant BS-90 strains of *B*. *glabrata*. Proteomic analyses of isolated plasma proteins revealed a diversity immune-related proteins including lectins, pathogen recognition receptors, cytotoxins, adhesion proteins, metalloproteinases, and Ca^2+^-binding proteins. Of the lectins, the variable immunoglobulin and lectin domain (VIgL) gene family of proteins comprised of fibrinogen-related proteins (FREPs), galectin-related proteins (GREPs) and C-type lectin-related proteins (CREPs), were highly represented, and consistent with their role in host immunity. Two proteins (GREP and a Zn-metalloproteinase) exhibited snail strain-associated protein and gene expression patterns suggesting their involvement in innate immune responses to larval infection. This comparative proteomic analysis of larval *S*. *mansoni*-reactive plasma proteins from susceptible and resistant *B*. *glabrata* strains represents the first of its kind and provides valuable insights into possible pathogen recognition receptors and other immune factors regulating parasite-host compatibility in this model system.

## Introduction

Human blood flukes of the genus *Schistosoma* are the causative agents of schistosomiasis, and represent one of the most important of the neglected tropical diseases (WHO, http://www.who.int/mediacentre/factsheets/fs115/en/). An estimated 230 million people are infected with this parasite and over 700 million are at risk of infection in over 70 resource-poor and developing countries worldwide [1–3]. All schistosome species require a compatible snail intermediate host in which asexually reproducing sporocysts give rise to the human-infective cercarial stage. For the blood fluke *Schistosoma mansoni*, miracidial penetration of, and successful development to the primary sporocyst within, its snail host *Biomphalaria* spp. is essential to the continued parasitic transmission to humans. The physiological changes occurring during this early period of intramolluscan larval development and the host responses to parasite infection result in a myriad of cellular, biochemical and molecular interactions that are still poorly understood [4–8].

Upon entry into susceptible strains of *Biomphalaria* spp., *S*. *mansoni* miracidia undergo dramatic morphologic and physiologic changes associated with early larval development. During this miracidium- to-sporocyst transition, the parasite produces and presents a complex repertoire of molecules to the snail host’s immune system in the form of glycoconjugates at the tegumental surface. In addition, other molecules, including proteins/glycoproteins, are released by miracidia as they shed their ciliated epidermal plates en route to become primary sporocysts [9,10]. Previous studies have shown that the molecules exposed at the sporocyst tegument and those released during *in vitro* larval development, termed excretory-secretory proteins (ESP; [9]) or larval transformation proteins (LTP; [10]), interact with numerous hemolymph proteins [11–14], although the identity of these reactive host molecules remains largely unknown. LTP have been shown to influence various immune-related hemocyte functions such as motility, phagocytosis, reactive oxygen species (ROS) production, and gene/protein expression [4,5,7] suggesting a possible role of specific LTP-hemolymph interactions in determining success or failure in establishing initial infections.

Cell-free hemolymph or plasma of *B*. *glabrata* snails contains a variety of proteins with putative immune functions, foremost among these being the large family of Variable Immunoglobulin and Lectin domain (VIgL) family of lectins that include the fibrinogen-related proteins (FREPs) [15–17], galectin-relate proteins (GREPs) and C-type lectin-related proteins (CREPs) [18]. FREPs of the VIgL family are distinguished from other recognized fibrinogen-related proteins [19] by possessing one or two immunoglobulin-like domains or Ig-folds in addition to a conserved C-terminal fibrinogen domain (FReD) [15]. Importantly, these VIgL FREP family proteins have been shown to be capable of binding to sporocysts and are responsive to larval infection [15,20]. Functional linkages between FREPs and snail susceptibility to trematode infections also have been established [21, 22], demonstrating a putative role of these plasma proteins in mediating larval immune recognition and/or host immune activation. In addition, recent evidence suggests that a diversified family of polymorphic mucins from *S*. *mansoni* sporocysts (*Sm*PoMuc) may be serving as targets or ligands for FREP reactivity [23,24]. Similar functional activities of GREPs and CREPs have yet to be investigated. Together, the ability of these lectins to interact with schistosome proteins lend support to the compatibility polymorphism hypothesis [25]: that the success or failure of establishing schistosome infections within its snail host depends on the reactivities of subsets of highly diversified immune receptors (e.g., FREPs) with their “matching” subsets of larval counter-receptors or ligands (e.g., *Sm*PoMucs).

In order to begin dissecting the molecular interplay during the critical early development of larval *S*. *mansoni* and its host snail *B*. *glabrata*, we have taken a proteomics approach combining affinity chromatography and proteomic analyses to enrich for and identify *B*. *glabrata* plasma proteins that are capable of binding larval proteins associated with the surface tegument of primary sporocysts or those released *in vitro* during early miracidium-to-sporocyst transformation (LTP). The overall aim of this research was to identify the major sporocyst-reactive host plasma proteins (and their encoding genes) that may be involved in mediating schistosome-snail compatibility/incompatibility under laboratory conditions. Following up on previous work [24], investigating the binding interaction between *B*. *glabrata* plasma and a sporocyst extract, we conducted a more comprehensive investigation of the “interactome” between constitutively expressed plasma proteins of susceptible and resistant *B*. *glabrata* strains and their recognized larval proteins associated with the sporocyst tegument and LTPs.

## Results

**Fig 1:**
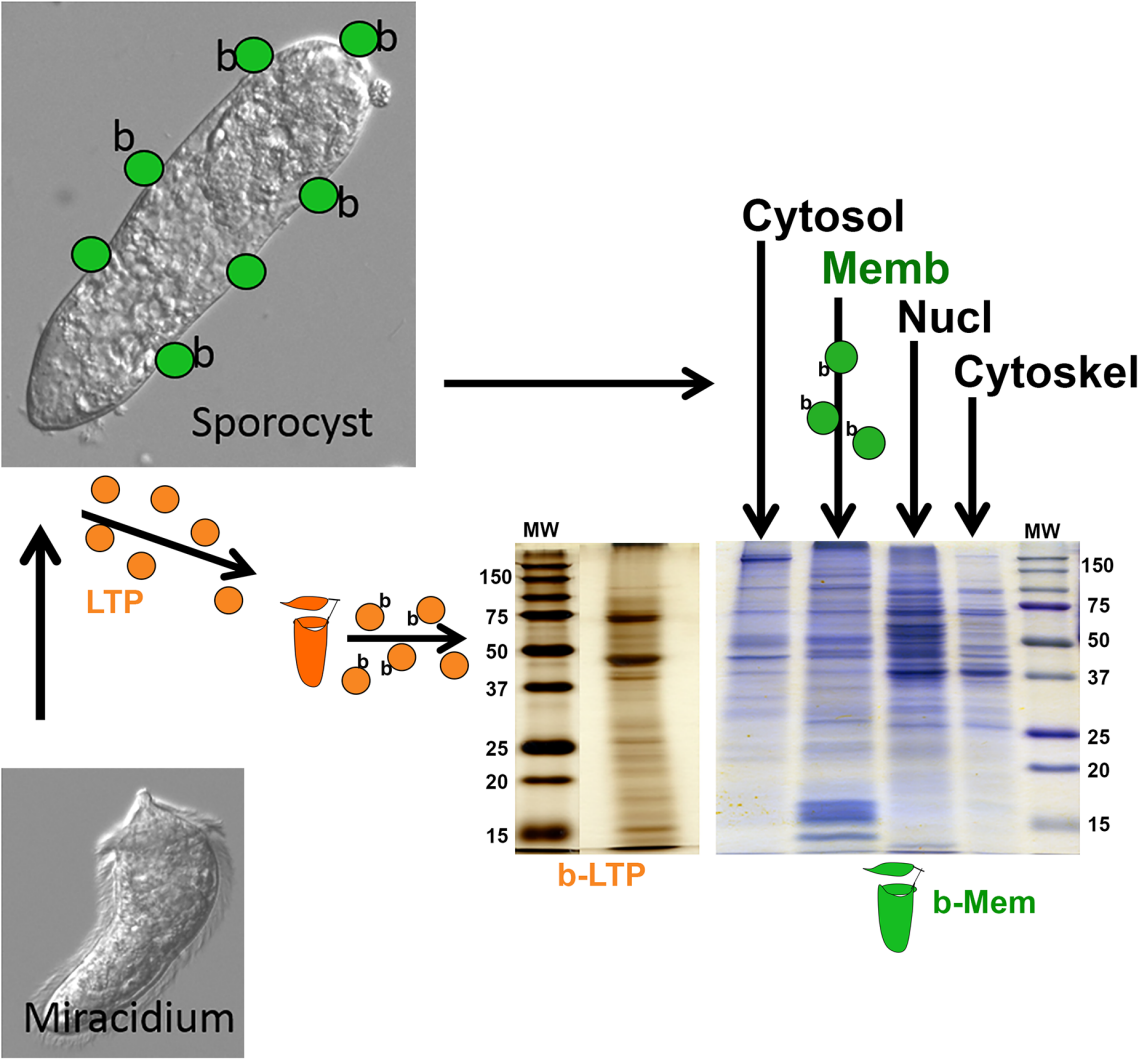
Schematic diagram illustrating the work-flow involved in the affinity-chromatographic isolation of larval *Schistosoma mansoni*-reactive *Biomphalaria glabrata* plasma proteins. Coomassie blue and silver stained SDS-PAGE gels show the protein profiles of the post-biotinylation sporocyst membrane-enriched fraction (b-Mem) and larval transformation proteins (b-LTP) used in the construction of affinity matrices.

As illustrated in **Fig** 1, larval protein pools obtained from the membrane-enriched fraction (Mem) of pre-biotinylated *S*. *mansoni* primary sporocysts and isolated larval transformation proteins (LTP) contained diverse populations of biotinylated proteins that served as immobilized “baits” in affinity chromatography pull-down experiments. Pooled plasma samples from resistant BS-90 and susceptible NMRI strains of *B*. *glabrata* snails introduced to the biotinylated membrane (bMem) and LTPs (bLTP) affinity columns yielded protein elution profiles that exhibited qualitative differences depending on the bait source used and snail strain (**S1 Fig**). In some cases, based on total unique peptide numbers, differences in relative protein abundance also could be observed. Control affinity columns, consisting of plasma introduced to columns containing only streptavidin beads (nonbiotinylated-protein control) or biotinylated protein columns eluted with TBS alone (buffer control), yielded little to no detectable eluted proteins.

Using the Protein Prophet algorithm’s criteria [26] for protein identification, 36 to 58 proteins were identified or predicted for all treatment groups (**S1 Table**) with >99% probability to achieve FDR < 1%. However, as detailed in the Methods section, the number of unique plasma peptides/spectra identified by MS analyses varied considerably between and within replicate treatment groups, thereby precluding a quantitative comparison of eluted protein subsets. Alternatively, we combined the peptides from all replicate datasets within each treatment group (matrix/snail strain) for protein identifications in order to provide a qualitative assessment of larval-reactive plasma proteins under each treatment condition. In some cases, we also used unique peptide abundance as an estimated measure of relative protein abundance, when feasible. When MS data were pooled in this manner, the number of total unique peptides and identified protein sequences representing each pooled treatment/snail group was generally consistent (**S1 Table**), enabling a qualitative approach to comparing protein populations comprising matrix-eluted plasma samples. The mass spectrometry proteomics data have been deposited to the ProteomeXchange Consortium via the PRIDE partner repository with the dataset identifier PXD004942 and 10.6019/PXD004942.

### Non-immune *Biomphalaria glabrata* plasma proteins

Proteomic analyses of plasma proteins eluted from both the sporocyst membrane (Mem) and larval transformation protein (LTP) affinity columns revealed similar predicted protein repertoires for the two snail strains. Not surprisingly, hemoglobin (Types 1 and 2) represented the most abundant proteins found in plasma eluates of both Mem and LTP columns. Approximately 50% of total unique peptides identified by MS/MS spectral analyses were assigned to *B*. *glabrata* hemoglobin, predominantly Type 1 (**S2 Table**). Other highly represented proteins in plasma eluates, mapped to the snail genome, including the common proteins, α-actins, α/β-tubulins, collagens α-1/5, and α-amylase. However, several proteins were unexpectedly found in relatively high abundance including acetylcholine-binding protein Type2 (second in unique peptide counts after hemoglobin), balbiani ring protein and apolipohorin (**S2 Table**). The majority of these eluted proteins was present in the plasma of both BS-90 and NMRI snail strains. However, α/β-tubulins, ATP synthases and Na/K-transporting ATPase were recovered almost exclusively from the Mem affinity column, suggesting a degree of tissue binding specificity among selected plasma proteins.

### *Biomphalaria glabrata* immune-related plasma proteins: Lectins

For plasma proteins with potential immune function, the largest single group with larval binding activity belonged to the Variable Immunoglobulin and Lectin domain (VIgL) family of plasma proteins. These included all three major groups; the *B*. *glabrata* fibrinogen-related proteins (FREPs) [15], galectin-related proteins (GREPs) and the C-type lectin-related proteins (CREPs) [18] (**Table 1**). Of the most widespread and diverse of VIgL gene family members, the FREPs, only three subfamilies were identified in plasma eluates: FREP2 and FREP3 eluted from both Mem and LTP affinity columns charged with NMRI and BS-90 snail plasma, and a FREP12, identified only from NMRI plasma eluates of the LTP column. In addition to the identified VIgL family of FREPs, several other FReD-containing proteins were recovered that matched several FREP-like proteins predicted in the nonredundant NCBI (NCBInr) protein database, including a ficolin-2-like, angiopoeitin-4-like and an uncharacterized FReD-containing protein (**Table 1**). Domain analyses showed that angiopoeitin-4-like and ficolin-2-like proteins both contained coiled-coil domains and a C-terminal FReD sequence, but lacked a N-terminal Ig-fold domain. The uncharacterized protein contained both single Ig-fold and fibrinogen domains, which, by definition, identified it as a putative VIgL family FREP. However, for this protein, no discriminating (i.e., unambiguous) sequence matches were found in NCBI or VectorBase, precluding its assignment to a specific FREP subfamily.

**Table 1:** VIgL domain proteins from NMRI and BS-90 *Biomphalaria glabrata* plasma eluted from *Schistosoma mansoni* sporocyst membrane-enriched (Mem) and larval transformation protein (LTP) affinity columns.

Protein Identification	Scaffold No.	NMRI	BS-90
**FREP 2.28** [*Biomphalaria glabrata*] (100%); Sequence ID: ADK11410	LGUN_random_Scaffold563550; 9301; 534902	**LDKDGVDSIQISR** **TDGGGWIIFQR** **VVVTLASGLK** **YQPVATSLYPSVTK** SSTDDLAVALSYIQDR DGFGDYDIGEFYLGNENIFK	**LDKDGVDSIQISR** **TDGGGWIIFQR** **VVVTLASGLK** **YQPVATSLYPSVTK** SSTDDLAVALSYIQDR
**FREP 3–2 precursor** [*Biomphalaria glabrata]* (100%); Sequence ID: AAK28656	LG3_random_Scaffold284: BGLB000204	**TDGGGWIIFQR** GVNWYDLSR INGNVDFYR	**TDGGGWIIFQR** LQSLYILHESK VYFSGSSDIIK YITLILHNPTQFDAR INGNVDFYR
**FREP12 FBG variant 2, partial** [*Biomphalaria glabrata*] (100%); Sequence ID: AAT58639	LGUN_random_Scaffold1434: BGLB000011 (86%) LGUN_random_Scaffold2702: BGLB000021 (86%) LGUN_random_Scaffold4104: BGLB0000133 (85%)	DGFGDYDIGEFYLGNENIFK LQIGDYLGNAGDDLSPHNNMFFSTFDR LTSTGQYDLR TDGGGWIIFQR YFAQYEDFK	No peptides
**PREDICTED: ficolin-2-like** [*Biomphalaria glabrata*] (100%); Sequence ID: XP_013071685 **PREDICTED: fibrinogen C domain-containing protein** [*Biomphalaria glabrata*]; (50%); Sequence ID: XP_013072469.1	LGUN_random_Scaffold11134	**IKNIQEDFDTR** **LVLNLASGLK** DGFGDYNIGEFYLGNENIYK DWQEYRDGFGDYNIGEFYLGNENIYK FNQGINWYGLTGYK FQIMNESNK FQIMNESNKYK GMVDFNR GMVDFNRDWQEYR NIQEDFDTR NNIPNCLFK QQNLSPFK RLVLNLASGLK SAYFSEMK SDRFNQGINWYGLTGYK VMCDTMTDGGGWIIIQR YPGPGGWWFGAGHGSNLNGVWR	**DGFGDYNIGEFYLGNENIYK** **IKNIQEDFDTR** **NIQEDFDTR** FNQGINWYGLTGYK FQIMNESNKYK LTSSGNYELR LVLNLASGLK QQNLSPFK RLVLNLASGLK SAYFSEMK SDRFNQGINWYGLTGYK YPGPGGWWFGAGHGSNLNGVWR
**PREDICTED: angiopoietin-4-like** [*Biomphalaria glabrata*] (100%); Sequence ID: XP_013069966 **PREDICTED: fibrinogen C domain-containing protein** [*Biomphalaria glabrata*] (47%); Sequence ID: XP_013093995.1	LG3_random_Scaffold99	**INTDLDSKEQQFTR** **LPTFEDLVNVIQK** **LTSTGQYDLR** **TDGGGWIIFQR** GQTVVTAIQEVGSNNVK IINQDLDNQK IINQDLDNQKQNIIR LLIQANDDREILQDNK MYKDLDNHEQNFIR SSDSGKYFCGAHVIGSDGR SSDTYVFVLLPSGLK YFAQYEDFK YFCGAHVIGSDGR	**LTSTGQYDLR** **TDGGGWIIFQR**
**PREDICTED: fibrinogen C domain-containing protein** [*Biomphalaria glabrata*] (100%); Sequence ID: XP_013072469	LGUN_random_Scaffold1199	**TDGGGWIIFQR** **VVVTLASGLK** VNSNGVIWNTLTK	**TDGGGWIIFQR** **VVVTLASGLK** GESASFTEIK SIEEDLNTK TIRDFIALITLDSSTLNLK TYGIVDFYR
**Galectin-related protein precursor (GREP)** [*Biomphalaria glabrata*] (100%); SequenceID: AKS26835.1	LGUN_random_Scaffold_18083; 47310; 120741; 5606	SLVLSQVEK	**AEDLIYFR** **ASLEITIEK** **ATLHFSVNSK** **FPDHGSEMYLK** **FPDHGSEMYLKR** **HETNGVLATISR** **LEANSIMEDNVPLK** **LEANSIMEDNVPLKNIEK** **SLVLSQVEK** **SLVLSQVEKR** **TFDVLYTIDKETLSLK** **VIQFGGTIVLHELSL** **YDNFYIDLYEDNYNINYQFR** **YVCGANIVNQEGQLEK** **TIKELLQPMIMK** LFANIKTPR SAISGLRFPDHGSEMYLK TFDVLYTIDK YVCGANIVNQEGQLEKLK ELLQPMIMK KLEANSIMEDNVPLK
**C-type lectin-related protein 2 precursor, partial (CREP2)** [*Biomphalaria glabrata*] (100%) Sequence ID: AKS26832	LGUN_random_Scaffold 35213; 34937; 205317; 46202; 113935	**GQPFAYSTEPR** SNSDDLVPALGR LIEDIFKWDDDNSICNPLCR SYLQVTR	**No peptides**
**C-type lectin-related protein 3 precursor, partial (CREP3)** [*Biomphalaria glabrata*] (100%); AKS26833	LG3_random_Scaffold2051; 38320	**WTDDDSISNLSSIQSFFR** IQEALNQNIEK LTTDDLTMVVSR	**No peptides**
**PREDICTED: uncharacterized protein LOC106065543** [*Biomphalaria glabrata*] (100%); Sequence ID: XP_013079834 **PREDICTED: C-type lectin domain family protein** [*Biomphalaria glabrata*] (28%); Sequence ID: XP_013081459.1	LGUN_random_Scaffold20861	**INSLVLSR** YASLNEVLQK	INSLVLSR YASLNEVLQK
**PREDICTED: uncharacterized protein LOC106062883 “CREP-like”** [*Biomphalaria glabrata*] (100%); Sequence ID: XP_013076651 **PREDICTED: C-type lectin domain family protein** [*Biomphalaria glabrata*] (32%); Sequence ID: XP_013081459.1	LGUN_random_Scaffold16778	**FTSLASVNTLDNK** **GGNWYMADAR** **NLFDVVVTGANDEEKEGTWIYNR** **VFNWATTEPNSGR** APLKVFNWATTEPNSGR NLFDVVVTGANDEEK CVYFNNKPNDGYVYSYLCEMPESSTDV IANCQCFWK LTWKNPMSEK RVNTPELSALFSLTLLHSNAMDEPK	**FTSLASVNTLDNK** **NLFDVVVTGANDEEKEGTWIYNR** **VFNWATTEPNSGR** GGNWYMADAR NLFDVVVTGANDEEK
**PREDICTED: uncharacterized protein LOC106062128** [*Biomphalaria glabrata*] (100%); Sequence ID: XP_013075857 **PREDICTED: techylectin-like protein** [*Biomphalaria glabrata*] (87%); Sequence ID: XP_013089083.1	LGUN_random_Scaffold6870	No peptides	EADTNKQDIIR QQNMLSIR
**PREDICTED: uncharacterized protein LOC106065054** [*Biomphalaria glabrata*] (100%); Sequence ID: XP_013079269 **Uncharacterized protein** containing C-type lectin, signal peptide, TM, EGF domains	LG4_random_Scaffold311	**CDFSKSESLIR** **CFNQCHCLGEPCNPK** **ENEGILLEHDLQVTIK** **FGLKEGSLIK** **GITPYDCYATSPDNK** **NSAYSVIGQPTELHK** **QLIDSQGCPMSGSPISAFSER** **SVECKPGSFGDK** **SVECKPGSFGDKCFNQCHCLGEPCNPK** **YGCQTGWMGNACDLEK** **YGCQTGWMGNACDLEKQDPDVK** **YYCVNSQEK** CKYGCQTGWMGNACDLEK GITPYDCYATSPDNKFK KQLIDSQGCPMSGSPISAFSER LENDIYIGLR LENDIYIGLRK NFGSSLVK	**CFNQCHCLGEPCNPK** **FGLKEGSLIK** **GITPYDCYATSPDNK** **NSAYSVIGQPTELHK** **QLIDSQGCPMSGSPISAFSER** LENDIYIGLR YYCVNSQEK
**PREDICTED: uncharacterized protein LOC106061984** [*Biomphalaria glabrata*] (100%); Sequence ID: XP_013075688 **PREDICTED: fibroleukin-like** [*Biomphalaria glabrata*] (45%) XP_013070005.1	LGUN_random_Scaffold15796	NVEGEIFDNESKDSYLQVTWNDLK TLIQGERDTSALQSLYLLHETNGVIAYINK	TLIQGERDTSALQSLYLLHETNGVIAYINK

Protein identifications (Sequence ID) were determined using Mascot and Sequest against the current *Biomphalaria glabrata* annotated NCBInr database. Letters highlighted in light gray = peptides recovered from *S*. *mansoni* Mem affinity columns; Letters highlighted in dark gray = peptides recovered from LTP affinity columns. Non-highlighted black lettering = peptides common to both Mem and LTP columns. See **S3 and S4 Tables** for predicted protein sequences and locations of coding regions within the *B*. *glabrata* assemblage (VectorBase) for each identified protein. For VIgL domain-containing proteins that lacked N-terminal sequence data, identifications were based on the highest NCBI BLASTp hit.

**Fig 2:**
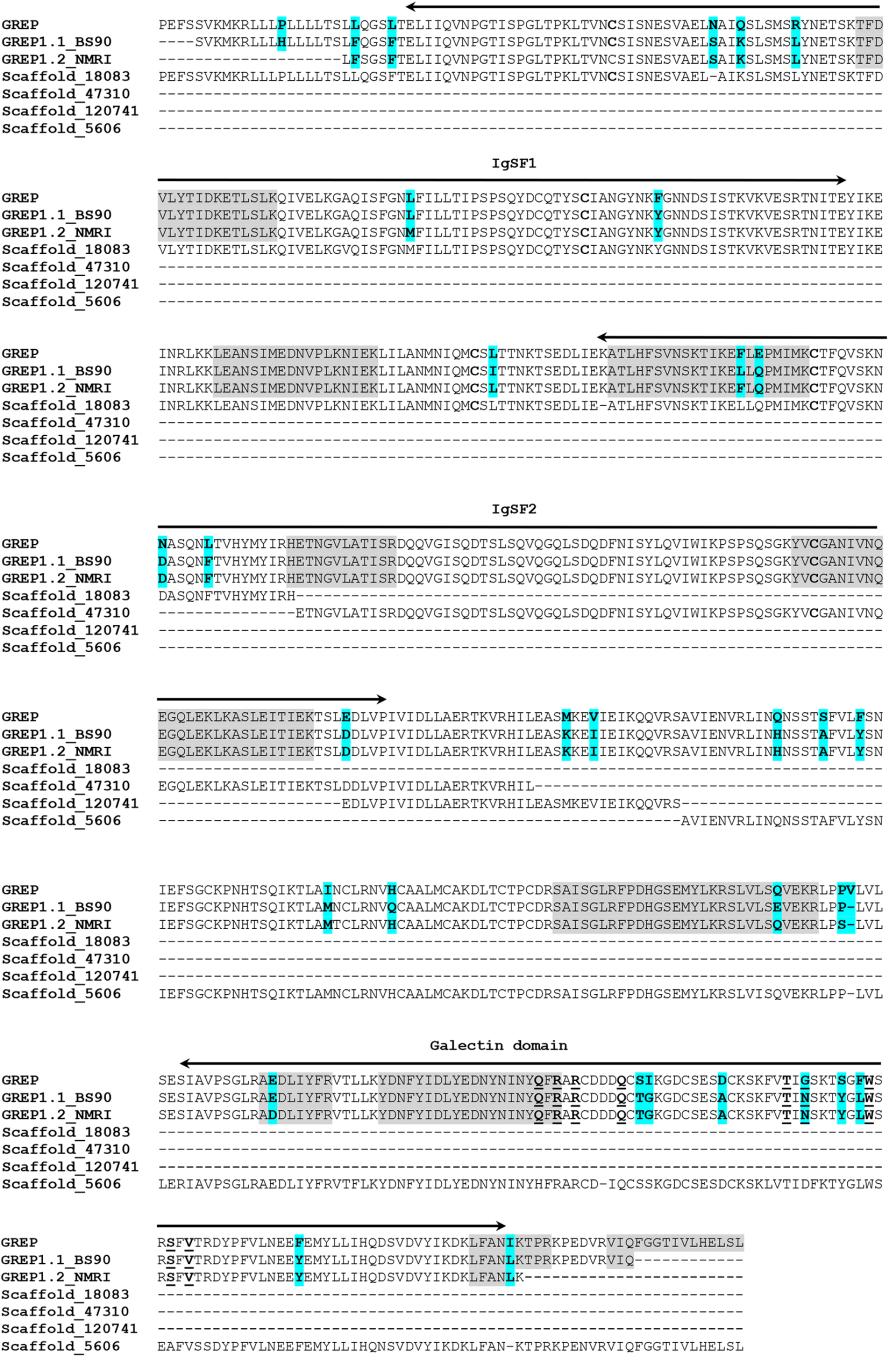
Multi-sequence alignment and comparison of the predicted galectin-related protein (GREP) sequences with the BS-90 and NMRI *Biomphalaria glabrata* variant GREP proteins. Amino acid sequence predicted from an RNAseq assembly (RNAseq-GREP; KM975647) [18] is compared to 4 predicted proteins encoded within Scaffolds18083, 47310, 210741 and 5606. Near-complete ORFs for the GREP, encoding IgSF1/IgSF2 domains and galectin binding domain, were amplified from BS-90 (GREP1.1) and NMRI (GREP1.2) snail cDNA. Original peptide sequences (shaded grey) aligned with the translated scaffolds and the GREP1.1 and 1.2 proteins. Cysteines involved in the formation of Ig-loop domains are shown in boldface, while residues associated with the sugar-binding pocket of the galectin domain, as predicted by NCBI delta-blast, are shown as underlined bolded letters (Q/H, R, R, T/Q, N/G/S, W, A/S, V). Amino acid differences between GREP [18], GREP1.1 and GREP 1.2 are highlighted in blue.

As previously mentioned, two other groups of VIgL domain-containing plasma proteins recovered from both Mem and LTP affinity columns were the galectin-related proteins (GREPs) and C-type lectin-related proteins (CREPs) [18] (**Table 1**). GREP-associated plasma peptide sequences were mapped to four Scaffolds in VectorBase (Scaffolds18083/47310/120741/5606) and multiple-protein sequence alignment of their predicted ORFs showed high identity to the GREP IgSF1, IgSF2 and galectin domains (**Fig 2**). This prediction was confirmed by RT-PCR amplification and sequencing of the nearly complete GREP transcript from BS-90 whole body cDNA. This GREP shared ~95% aa identity with the published GREP protein sequence [27] and is designated here as a new GREP variant, GREP1.1 (Accession no. KX950826). A second GREP variant (designated GREP1.2; Accession no. KY095833) also was identified by sequencing PCR-generated amplicons using GREP primers and cDNA from the NMRI snail strain. Alignment of the translated BS-90 (GREP1.1) and NMRI (GREP1.2) snail proteins with the published GREP sequence [18] revealed consistent strain-specific aa substitutions (**Fig 2)**.

Five C-type lectin (CTL)-domain containing proteins were eluted from both Mem and LTP columns, two of which were identified as C-type lectin-related proteins (CREP2 and CREP3) encoded in multiple Scaffolds within the *B*. *glabrata* genome (**Table 1**). One of the three remaining CTL-domain proteins, identified as uncharacterized protein LOC106062883 (Scaffold 16778), also contained an Ig-fold domain, in addition to a CTL binding region. This protein was designated a CREP-like protein but could not be assigned to a CREP subfamily (**S3 and S4 Tables**). The absence of N-terminal sequence information for the other CTL-containing proteins precluded further identification as CREP family members. In addition, as noted for the GREP, peptides for both larval-reactive CREP2 and 3 were recovered only from NMRI *B*. *glabrata* plasma eluates. Protein sequences and locations of coding regions within VectorBase for all identified immune lectins are shown in **S3 and S4 Tables**.

### *Biomphalaria glabrata* immune-related plasma proteins: Other immune proteins

In addition to the VIgL-family of lectin receptors, proteomic analyses also identified or predicted numerous other immune-related proteins recovered from the larval Mem and LTP affinity matrices. Foremost among these were pathogen recognition receptors/adhesion proteins (LPS-binding protein, TEPs, dermatopontins), cytolysins (biomphalysin), metalloproteases (A Disintegrin And Metalloproteinase with thrombospondin domain (ADAM-TS), Zn-metalloproteinases (Zn-MP) with ADAM/reprolysin type domains), stress proteins (HSP60 and 70), oxidative enzymes (GATA Zn-finger/Cu-Zn SOD, monooxygenase), signaling proteins (14-3-3, TGFβ-induced protein) and a Ca^2+^-binding protein (**Table 2**). Several of these proteins were notable due to their relative high representation/abundance in plasma eluates. Among these were three proteins, initially identified as thioester-containing protein (TEP) family members (TEP, TEP1.5, and CD109-like antigen), and a predicted complement C4-like protein. Peptides identified for the TEP1.5 and C4-like proteins matched those of the TEP sequence, and therefore likely represent the same protein. The CD109-like protein clustered with the TEP family, but differed from the identified *B*. *glabrata* TEP (**Table 2**). TEPs were common in plasma eluates of both NMRI and BS-90 snail strains.

**Table 2:** Other immune-related proteins from NMRI and BS-90 *Biomphalaria glabrata* plasma eluted from *Schistosoma mansoni* sporocyst membrane-enriched (Mem) and larval transformation protein (LTP) affinity columns.

Protein Identification	Scaffold No.	NMRI	BS90
**PREDICTED: Lipopolysaccharide-binding protein-like** [*Biomphalaria glabrata*] (100%); Sequence ID: XP_013070669.1	LGUN_random_Scaffold1023	IVGNVAQFR ALDDGMNMVVIPQLNAVGDTGIALPVLK DLTFQNPR QIPLESDIADKFVIDYR	**IVGNVAQFR** ALDDGMNMVVIPQLNAVGDTGIALPVLK DLTFQNPR LLSPVLFTDSYLETQHK
**Thioester-containing protein (TEP)** [*Biomphalaria glabrata*] (100%); Sequence ID: ACL00841.1	LGUN_random_Scaffold4524; LGUN_random_Scaffold10725; LGUN_random_Scaffold9700; LGUN_random_Scaffold8482; LGUN_random_Scaffold2300; LGUN_random_Scaffold76986; LGUN_random_Scaffold7766; LGUN_random_Scaffold45951; LGUN_random_Scaffold53075; LGUN_random_Scaffold6063; LGUN_random_Scaffold7260	**FTPNIHIGR** **IIDLLNDGYQR** **NIAFTLPDSLVPESQR** **TYDYYEPANQATVFYQPR** HDTGMVVQELSIPSGFVPDLSTLGQVAGVK ITSTEAIDSLAYEIR QNIDGSFNEFGK SFFPESWLWTSVK SNMAADAIVR SQLAQSTFEK SYSMSNNYLQLSLLSK TETVSGAESQFFEVAEYDLPR VFRPFFVSLTYPR VGPSWAPIAQLLMYYIR DSTVCDVCPNCCP TLRDSTVCDVCPNCCP QNLDGSFNEFGK	**IIDLLNDGYQR** **ITSTEAIDSLAYEIR** **NIAFTLPDSLVPESQR** **NIAFTLPDSLVPESHR** **SFFPESWLWTSVK** **TYDYYEPANQATVFYQPR** ATNQLSEELNKK FTPNIHIGR LDEALPSVR QNIDGSFNEFGK SQLAQSTFEK SYSMSNNYLQLSLLSK VFRPFFVSLTYPR VRSFFPESWLWTSVK ENPFLTPVTPGPGNQASNIQVR QNLDGSFNEFGK
**PREDICTED: CD109 antigen-like** [*Biomphalaria glabrata*] (100%) Sequence ID: XP_013071291.1	LGUN_random_Scaffold108	**SVNQLSGDIQTK** **VTQPDGLPMTSTAEQVK** IYNVPVLIDLTEGK SVYFPIVPADLGK TTNTWDYPNPTK ALPVTTLQVPLNGEAK	IYNVPVLIDLTEGK NQVTAFYQSQILK SVYFPIVPADLGK TVDLTLPSNTVK VTQPDGLPMTSTAEQVK
**PREDICTED: Heat shock protein 70 kDa cognate 4** [*Biomphalaria glabrata*] (100%) Sequence ID: XP_013082115.1	LGUN_random_Scaffold253: BGLB007783	ARFEELNADLFR IINEPTAAAIAYGLDK IINEPTAAAIAYGLDKK	FEELNADLFR IINEPTAAAIAYGLDK IINEPTAAAIAYGLDKK LLQDFFNGK QTQTFTTYSDNQPGVLIQVYEGER
**Heat shock protein 60** [*Biomphalaria glabrata*] (100%); Sequence ID: ACL00842	LGUN_random_Scaffold791: BGLB013300	ALMLQGVDLLADAVAVTMGPK	ALMLQGVDLLADAVAVTMGPK KPLLIVAEDVDGEALSTLVLNR
**Biomphalysin** [*Biomphalaria glabrata*] (100%); Sequence ID: AGG38744.1	LGUN_random_Scaffold10: BGLB000137	**ASSPVTESIER** **FGDSSVPFYK** **FKGYLFNLESAR** ADGDDLYFLK ADGDDLYFLKK GYLFNLESAR NGFTWAADTR SVIEDLQAESVDSGVLYNR TNSGRPTFNYR TTVPYTAIITTK VGYFLQGLYR WEDGNGNFHQDYR	FGDSSVPFYK FKGYLFNLESAR SVIEDLQAESVDSGVLYNR
**Dermatopontin 2** [*Biomphalaria glabrata*] (100%); Sequence ID: AAZ80786	LGUN_random_Scaffold8114:1204:2972: BGLB013420	**LTLVVPEGTAVK** CPDGQVVSYVSSIHNNRR FGFYCCDVQGSTPR QTHSCTDSGYVNDFDGPLVYTCPGNK WQFQICTL	CPDGQVVSYVSSIHNNRR FGFYCCDVQGSTPR LTLVVPEGTAVK QTHSCTDSGYVNDFDGPLVYTCPGNK
**PREDICTED: Millepora cytotoxin-1-like** [*Biomphalaria glabrata*] (100%); Sequence ID: XP_013061974.1	LG24_random_Scaffold7889	**FYCCTNKDYLVHACQHTLTINNAK** **GLFSSYDSTAGDR** **GLFSSYDSTAGDRLYR** VPEAMYVR GFISFRVPEAMYVR	**GLFSSYDSTAGDR** GLFSSYDSTAGDRLYR
**PREDICTED: Uncharacterized protein LOC106068784** [*Biomphalaria glabrata*] (100%); Sequence ID: XP_013083707.1 **Uncharacterized protein** containing a Zn-dependent metalloprotease ADAM/reprolysin-like subgroup domain	LGUN_random_Scaffold286	SLPQIYNNLLPK AIGNLLGASNDGALSTSIMAIINAPGDVNR CSLGQCSSYSTPVVDTNCVFGDQK GNAVCGQIFCR IPNTMTCTPVYTSDGLVCDNQKR	AIGNLLGASNDGALSTSIMAIINAPGDVNR SLPQIYNNLLPK CSLGQCSSYSTPVVDTNCVFGDQK
**PREDICTED: A disintegrin and metalloproteinase (ADAM) with thrombospondin motifs** [*Biomphalaria glabrata*] (100%); Sequence ID: XP_013075983.1	LGUN_random_Scaffold16032	TYHTALLTAQQISELLGSQHDGVLSSITNR WFFSSAVAGDIK YAVSMSEFDR YAVSMSEFDRTYHTALLTAQQISELLGSQHDGVLSSITNR	**No peptides**
**PREDICTED: Serpin B6-like** [*Biomphalaria glabrata*] (100%); Sequence ID: XP_013077880.1	LGUN_random_Scaffold1827	**IQSDVIDLVPALK** **SNLNQLLQNMK** **VDRPFLYVIR**	**No peptides**
**PREDICTED: 14-3-3 protein epsilon-like** [*Biomphalaria glabrata*] (100%); Sequence ID: XP_013082420.1	LGUN_random_Scaffold260: BGLB007883	DSTLIMQLLR	AAFDDAIAELDTLSEESYK AAFDDAIAELDTLSEESYKDSTLIMQLLR DSTLIMQLLR YLAEFATGNDR
**PREDICTED: Transforming growth factor-beta-induced protein** [*Biomphalaria glabrata*] (100%); Sequence ID: XP_013079848.1	LGUN_random_Scaffold209	FTTFYQLLK LPAGSLDALK LSAETLDYLNNHISDLTDVLR SDISTFESLLVK	No peptides
**PREDICTED: Neo-calmodulin-like** [*Biomphalaria glabrata*] (100%); Sequence ID: XP_013067017.1	LG27_random_Scaffold161:BGLB001498	**SLGQNPTEAELQDMINEVDADGNGTIDFPEFLTMMAR** **VFDKDGNGFISAAELR** EAFSLFDKDGDGTITTK	**VFDKDGNGFISAAELR** EAFSLFDKDGDGTITTK
**PREDICTED: Uncharacterized protein LOC106079481** [*Biomphalaria glabrata*] (100%); Sequence ID: XP_013096101 **Ca-binding protein 1-like (100%); Sequence ID:** AAV91525.1	LGUN_random_Scaffold6449	ADYTAAIDENFPNETHDPIITNAYR FFQTYDANHDNR	ADYTAAIDENFPNETHDPIITNAYR FFQTYDANHDNR
**PREDICTED: GATA zinc finger domain-containing protein** [*Biomphalaria glabrata*] (100%); Sequence ID: XP_013076718.1 **Superoxide dismutase (Cu-Zn)** [*Megathura crenulata*] (40%); XP_013083707.1, CAF22060.1	LGUN_random_Scaffold1689	**DLTAKNEIPIFAR** **EGLLEKDLTAK** **HLNEQYKPNLK** **HNPIQIHIYKGEK** **NEIPIFAR** **QFLNLSSNDISLNK** **YNHIHSLNNQDLR** **YNHIHSLNNQDLRR** GCHSMGGHFNPEGVHHGYR HNPIQIHIYK MSVEQIKK NDDDNLQDHVHVHSK QLHGFHVHEFGDSSK QLNKNDDDNLQDHVHVHSK	**DLTAKNEIPIFAR** **EGLLEKDLTAK** **HLNEQYKPNLK** **HNPIQIHIYKGEK** **NEIPIFAR** **QFLNLSSNDISLNK** HNPIQIHIYK MSVEQIKK QLHGFHVHEFGDSSK
**PREDICTED: Uncharacterized protein LOC106059379** [*Biomphalaria glabrata*] (100%); Sequence ID: XP_013072448.1 **Uncharacterized protein** containing **Cu-type II ascorbate-dependent monooxygenase**, C-terminal domain	LGUN_random_Scaffold1196: 16772–16869; 15959–16119; 14884–15048; 11587–12745	**QHVQILPGDEIITR** **SQSIPVDGCDWK** TFLSYGDLELIDMYCQLR	**QHVQILPGDEIITR** GLVSTIR SQSIPVDGCDWK

Protein identifications (Sequence ID) were determined using Mascot and Sequest against the current *Biomphalaria glabrata* annotated NCBInr database. Letters highlighted in light gray = peptides recovered from *S*. *mansoni* Mem affinity columns; Letters highlighted in dark gray = peptides recovered from LTP affinity columns. Non-highlighted black lettering = peptides common to both Mem and LTP columns. See **S5 and S6 Tables** for predicted protein sequences and locations of coding regions within the *B*. *glabrata* assemblage (VectorBase) for each identified protein.

Other highly represented immune-related proteins included a GATA Zn-finger domain protein containing a Cu-Zn SOD binding domain, biomphalysin, several Zn-metalloproteinases (Zn-MP), dermatopontins (dermatopontin2 and *Millepora* cytotoxin containing a conserved dermatopontin domain), LPS-binding protein and HSP70. The Zn-MPs were a diverse group of enzymes that included ADAM-TS and two other Zn-MP identified as uncharacterized proteins. The ADAM-TS was among several proteins that were exclusively recovered from plasma of the NMRI snail strain (ADAM-TS, serpin B6-like) or were eluted only from the Mem affinity column (HSP60, HSP70, ADAM-TS, 14-3-3, TGF-β-induced protein) (**Table 2**). It is important to keep in mind, however, that these protein “abundances” are only estimates based on the assumption that the relative number of unique peptides identified by MS is generally correlated with protein abundance in our pooled dataset. Predicted protein sequences and locations of coding regions for identified immune proteins within VectorBase are shown in **S5 and S6 Tables**.

### Evaluation of differential transcript expression between *B*. *glabrata* strains

**Fig 3:**
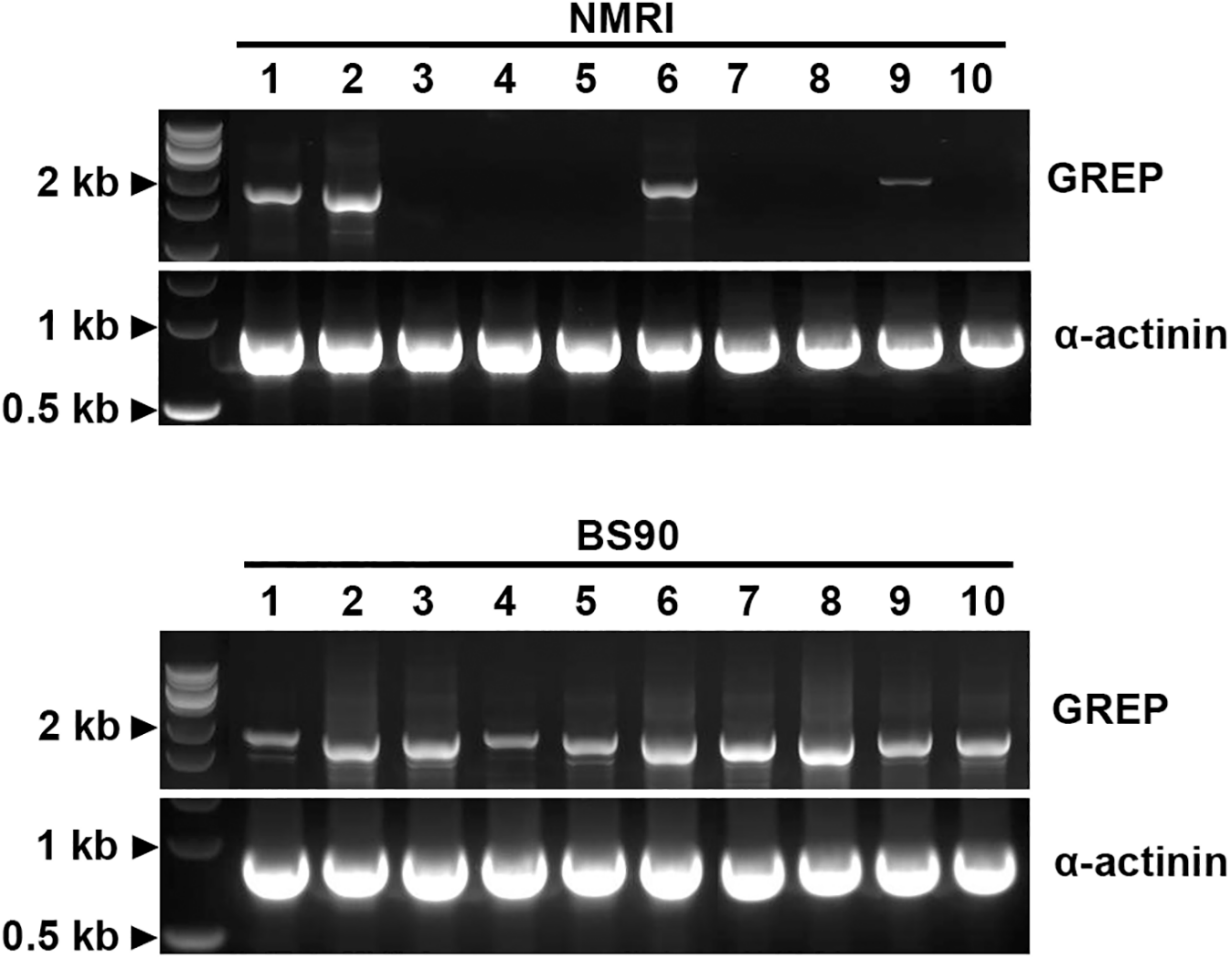
PCR amplification of NMRI and BS-90 *B*. *glabrata* galectin-related protein (GREP) transcripts. Complementary DNA synthesized from whole body RNA extracts of 10 individual NMRI and 10 BS-90 *B*. *glabrata* snails were used to generate amplification products of the near-complete coding region of the BS-90 GREP sequence. GREP amplicons for each snail sample (1–10) are shown. Primers to *B*. *glabrata* α-actinin served as a loading control. Note that GREP amplicons were generated using cDNA from all BS-90 samples tested, while only 4/10 NMRI snails produced amplicons, demonstrating differential GREP gene expression in the NMRI snail population.

**Fig 4:**
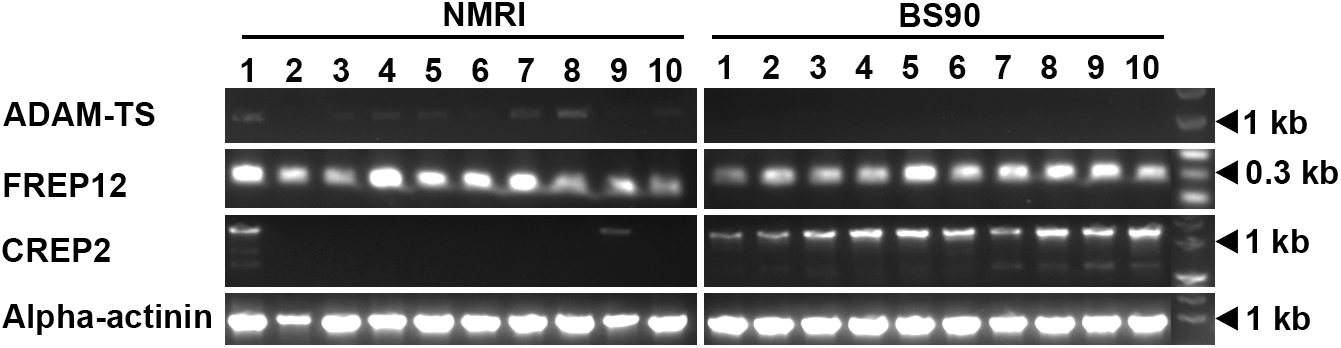
PCR amplification of NMRI and BS-90 *B*. *glabrata* ADAM-TS, FREP12, and CREP2 transcripts. Whole body total RNA from 10 individual NMRI and 10 BS-90 *B*. *glabrata* snails were subjected to cDNA synthesis and used in PCR analysis of the ADAM-TS metalloproteinase, FREP12 and CREP2 transcript expression. Amplicons of the predicted size are shown for each snail sample (1–10). Primers to *B*. *glabrata* α-actinin served as a loading control.

**Fig 5:**
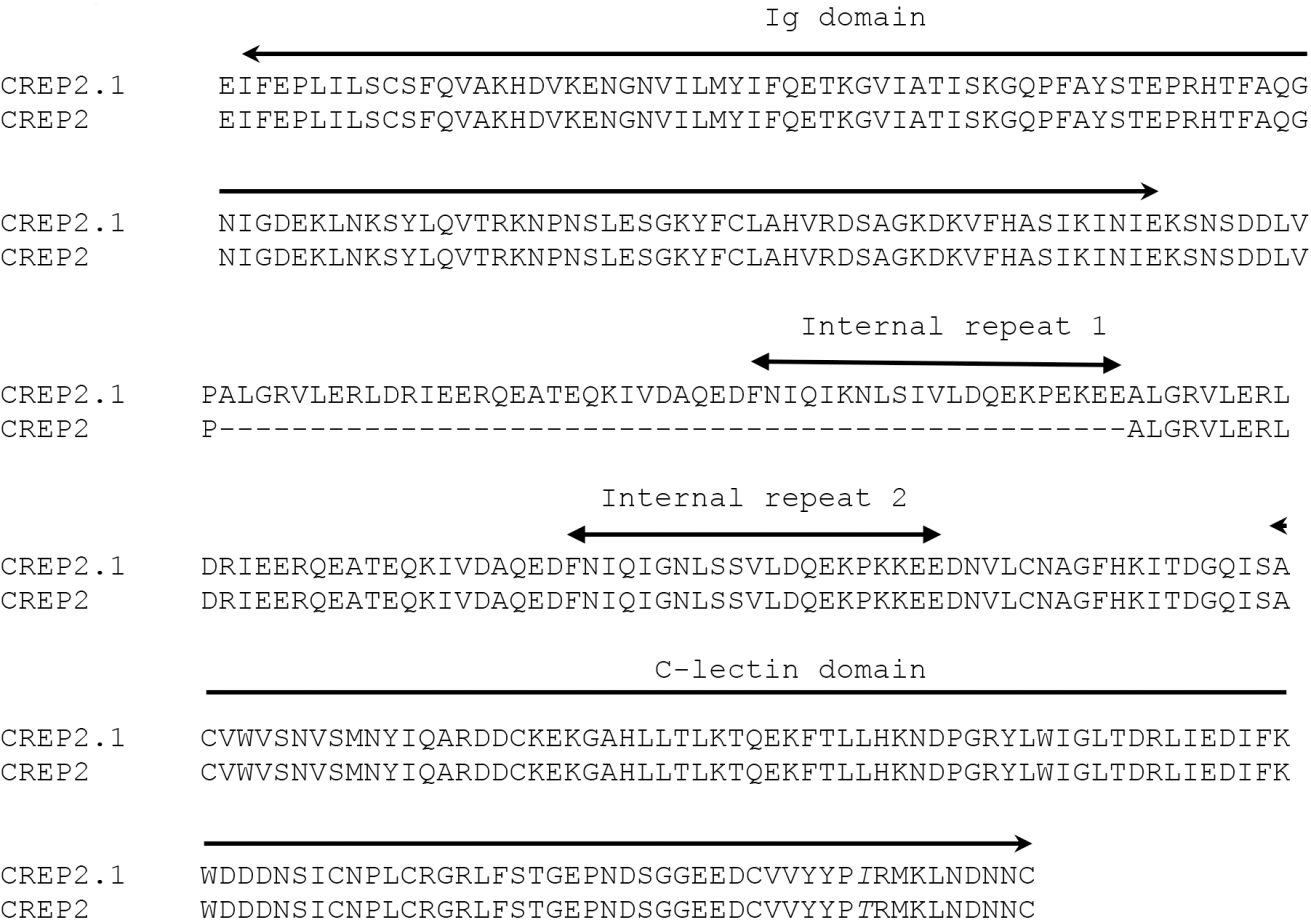
Alignment of a partial C-type lectin-related protein 2 (CREP2.1) sequence with the predicted CREP2 protein. Amino acid sequence predicted from an RNAseq assembly (CREP2; AKS26832.1) [18] is compared to a partial ORF encoding a CREP2, presenting an Ig domain, 2 internal repeat domains and a C-type lectin domain, from BS-90 and NMRI snail cDNA (CREP2.1). CREP2.1 presented an additional 50 aa sequence containing an internal repeat domain (internal repeat domain1) and a single aa difference (italics) when compared to CREP2.

Since we were interested in larval-reactive plasma proteins that may be differentially expressed in either resistant (BS-90) and susceptible (NMRI) *B*. *glabrata* strains, we followed up on selected proteins that were identified in our initial proteomic analyses to be exclusively recovered from plasma of a single snail strain and exhibited a minimum of four unique peptides. For these experiments forward and reverse primers were synthesized to coding regions for the following proteins: GREP, FREP12, CREP2, and ADAM-TS (**S7 Table**). Comparative PCR analyses of cDNA derived from 10 individual *B*. *glabrata* snails of each strain demonstrated that gene expression levels varied both within and between snail strains. For the GREP, all ten BS-90 cDNA samples produced amplicons of predicted size, whereas 4 of 10 NMRI snail samples were also positive (**Fig 3**). Similarly, transcript expression of the ADAM-TS gene, although weak, was evident in 7 of 10 NMRI snails while all tested BS-90 samples were completely negative (**Fig 4**). For both the GREP and ADAM-TS, gene expression patterns generally were consistent with their respective patterns of protein expression. However, in contrast to the general correlation of transcript and protein expression, PCR products for FREP12 were amplified from cDNA of both snail strains (**Fig 4**) despite the identification of FREP12 peptides exclusively from NMRI plasma (**Table 1**). Unexpectedly, for CREP2, transcript expression was completely opposite that of its protein expression. In this case, CREP2 peptides were recovered only from eluted NMRI plasma samples (**Table 1**), whereas CREP2 transcripts were expressed in all BS-90 snails and only 2 NMRI snail (**Fig 4**). Because of the inconsistency of protein vs. transcript expression for FREP12 and CREP2, amplicons from three individual snails of each *B*. *glabrata* strain were sequenced. All amplified FREP12 sequences from both strains were identical, likely representing the same protein. Similarly, all of the aa sequences of the translated PCR-amplified CREP2 bands aligned exactly to the previously published CREP2 [18], except for a 50 aa gap in the repeat region of the published sequence (**Fig 5**). This appeared to be a CREP2 variant and was designated CREP2.1 (Accession number KX950827). Finally, sequencing of GREP transcripts from individual BS-90 and NMRI strain snails revealed consistent single aa sequence differences between strains (**Fig 2**) and with the published GREP sequence [18], demonstrating probable genetic polymorphism within this VIgL group of proteins. Primers specific for *B*. *glabrata* α-actinin, common to both strains, produced amplicons of similar intensity and predicted size using cDNA of BS-90 and NMRI snails, indicating integrity of sample and similar sample loading. Sequencing of selected bands from both BS-90 and NMRI confirmed the identity of amplicons.

## Discussion

Within the first 24 hr following infection of the snail host by *S*. *mansoni* miracidia, there is rapid and intimate contact between host plasma and the parasite, specifically the tegumental surface of the newly developing primary sporocyst and products, mainly glycoproteins, released during miracidium-to-sporocyst transformation (termed larval transformation proteins or LTPs [10]). In *Biomphalaria* spp.*-S*. *mansoni* model systems, plasma has been shown to play an integral role in regulating the cellular immune response to larval infection depending on the degree of compatibility exhibited between the snail and its schistosome parasite [27–29]. For incompatible host-parasite pairings, it is hypothesized that plasma factors may be serving as immune recognition molecules targeting the parasite for hemocyte encapsulation and destruction [4–6,8]. However, for compatible host-parasite relationships, the binding of plasma factors to the larval surface may serve a parasite-protective role by disguising the parasite from immune recognition or by blocking of immune factors by secreted larval molecules [4,5,8]. Although there is evidence for the involvement of *B*. *glabrata* FREPs [21, 22, 30] and stress proteins [31–33] in various immune processes, the full spectrum of larval-interactive plasma proteins that may be contributing to the regulation of host immune responses, has not been explored.

In the present study, using a combination of affinity chromatographic and proteomics approaches, we were able to enrich for, and identify, specific plasma proteins that exhibited binding reactivity to two groups of larval proteins most likely to be involved in immune interactions; namely proteins associated with the sporocyst tegument and with larval transformation proteins or LTPs. Using this combined approach, the most prominent group of immune-related, larval-reactive plasma proteins were members of the variable immunoglobulin and lectin domain (VIgL) family including several fibrinogen-related proteins (FREPs) [15], C-type lectin-related proteins (CREPs) and galectin-related proteins (GREPs) [18]. The appearance of multiple FREPs was not surprising as these molecularly diverse proteins are highly represented in snail plasma [16] and have been shown to be capable of binding to larval schistosomes [15]. Genes encoding these FREPs also have been reported from other gastropod species including *Aplysia californica* [34] and *Littorina littorea* [35].

One unexpected result that arose in our analysis of VIgL-FREP family proteins was the finding of proteins encoded in the *B*. *glabrata* genome that possessed a C-terminal fibrinogen domain (FReD), but lacked an N-terminal Ig-fold. The FReD-containing angiopoeitin-4-like and ficolin-2-like proteins identified in our study (Table 1) appear to represent a different class of FREPs [19] in *B*. *glabrata* that had to be distinguished from VIgL-FREP family members. Interestingly, when protein sequences that contained both Ig-fold and lectin domains were subjected to NCBI BLASTp searches, occasionally these would not be identified as members of the VIgL lectin family (e.g., FReD-containing protein XP_013072469, CTL-containing uncharacterized protein LOC106062883) based on gene predictions. These may well represent new VIgL-FREP and CREP subfamily members, respectively, but relatively low homology matches (typically <50% identity) to known FREPs/CREPs in the NCBI and VectorBase databases prevents placement into specific VIgL-lectin subfamilies. It should be noted that in our attempts to determine VIgL-FREP identities, in some cases, only partial sequences were predicted that did not include the N-terminal sequence containing the FREP-defining IgSF domain(s) [36]. However, regardless of the incomplete nature of sequence information for some FREP subfamilies and complications posed by the presence of non-VIgL FREPs in *B*. *glabrata* plasma, this study has clearly demonstrated that FREPs are highly capable of direct binding to proteins displayed at the sporocyst surface or released during early larval development.

Based on VIgL-FREPs identified from affinity column elutions, only a subset of FREP subfamilies (FREPs 2, 3, and 12) were detected with the capacity to bind larval proteins, suggesting a degree of selectivity in the interactions between sporocyst and FREP subfamily proteins. However, it is also quite possible that other FREP VIgL subfamily members may be binding larval proteins, but in quantities below the limits of MS-MS detection. In addition, although the parameters used in our MS analyses to identify each protein were stringent, due to the highly conserved nature of FREP subfamilies, definitive identification of specific FREPs may require further study. Regardless, Mem and LTP reactivity by FREP2 and 3 is consistent with previous reports of plasma-sporocyst interactions. In a sporocyst extract-*B*. *glabrata* plasma interaction study, a FREP2 was recovered from an immune complex pulled down with an anti-*S*. *mansoni* polymorphic mucin (*Sm*PoMuc) antibody [24]. Similarly, FREP2 and 3 were identified in a *B*. *glabrata* plasma-*Echinostoma* secretory-excretory protein complex [15]. Our recovery of larval-reactive thioester-containing proteins (TEPs) from eluted plasmas of both snail strains, in addition to FREP2, also mirrors a previous report [24] suggesting that the formation of immune complexes comprised of host FREPs/TEPs and parasite glycoconjugates (e.g., polymorphic mucins) may be involved in regulating host-parasite interactions. The identified FREP12 variant was of interest due to an apparent differential protein expression in the NMRI *B*. *glabrata* strain. However, at the transcriptional level, gene expression was detected in both NMRI and BS-90 strains. In this case, given the large number of FREP12 variants reported in *B*. *glabrata* (Supplementary File3 [18]), it is not surprising that transcript expression was detected in both strains since primers used apparently recognized sequences shared in common with other FREP12 variants.

Building on the finding of a galactose-binding protein in a plasma-sporocyst precipitation complex [24] and the recent discoveries of two additional VIgL domain family members (GREPs and CREPs) in *B*. *glabrata* [18,37] and other gastropod molluscs [34,35], we have identified several constitutively expressed GREPs and CREPs that are capable of binding proteins displayed by early developing *S*. *mansoni* larvae. Of special interest was the GREP protein that was recovered almost exclusively from BS-90 plasma eluates, again suggesting snail strain-specific differential protein expression. To corroborate the snail strain-specific GREP protein expression, a comparison of GREP transcript expression was conducted between NMRI and BS-90 *B*. *glabrata* strains. However, although 100% of BS-90 snails consistently showed strong GREP gene expression, 40% (4/10) of NMRI snails tested also expressed GREP transcripts. Therefore, although originally appearing to be a resistant snail-specific protein marker, GREP transcripts clearly are variably expressed in the susceptible NMRI snail strain. Of note, the sequencing of the BS-90 and NMRI amplicons (GREP1.1, GREP1.2) showed small, but consistent differences between each other and with the original GREP, suggesting polymorphism between snail strains. Given that the infection rate for our NMRI snail strain exposed to NMRI *S*. *mansoni* miracidia is approximately 80%, it would be interesting to see if GREP gene or protein expression may be linked to a residual resistant phenotype observed in this strain. Previous studies have shown that FREP3 protein and transcript expression are directly associated with *B*. *glabrata* resistance to *S*. *mansoni* infection [21,22,38]. Our finding of differentially expressed GREPs with larval binding activity now suggests that VIgL family lectins, other than FREPs, also may be potential determinants of immune compatibility in this host-parasite model system. An important question currently being addressed relates to the specific parasite molecules that serve as ligands interacting with GREP, and other VIgL family proteins. Because the schistosome proteins immobilized in Mem and LTP columns were enriched in glycoptoteins likely containing lacdiNAc (GalNAc β1–4 GlcNAc) and Lewis X oligosaccharides [39,40], it is probable that GalNAc and/or Gal residues are serving as ligands for galectin CRD-binding [41].

C-type lectins (CTLs) also were well represented as immune lectins, although only two were identified as belonging to the VIgL family of CREPs; namely CREP2 and CREP3. Contrary to the protein expression pattern exhibited by the GREPs, unique peptides for CREP2 and 3 were only recovered from NMRI plasma eluates of Mem and LTP columns. This finding raises questions as to whether the parasite may be using these immune proteins to coat or mask itself, thereby evading host immune recognition, or as a means of directly counteracting host responses by forming ineffective immune complexes [4–6,8]. Recent reports that larval *S*. *mansoni* produce a highly diversified population of polymorphic mucins [23,42] capable of binding plasma lectins [24] support the notion that larval counter-immune mechanisms may play an important role in successful establishment of infections [5,6,8]. Indeed, the release of a barrage of larval proteins during miracidial transformation that directly react to or complexes with soluble immune lectins could nullify their recognition or effector functions [9, 10]. In addition, many of the glycans associated with glycoprotein of *S*. *mansoni* sporocysts and LTP also are found naturally occurring in the plasma and other tissues of the snail host [38,43,44]. This sharing of glycans between host and parasite also may be reducing the “antigenicity” of invading larvae through a molecular mimicry-type mechanism [45]. Whether by mimicking or interfering with host molecules, the spectrum of recognizable ligands available for immune lectin reactivity is greatly diminished, and may be serving as a key evolutionary driver of host lectin diversification within snail populations or strains [6,8,25]. Further studies aimed at identifying the specific interacting partners comprising immune complexes [24,25] that are responsible for modulation of immune reactivity between parasite and host are needed.

One perplexing result was the contrary finding that, while larval-reactive plasma of NMRI snails exclusively contains CREP2 peptides compared to BS-90 snail plasma (Table 1), transcripts for CREP2 exhibited an opposite transcript expression pattern (Fig 3). A likely explanation is that the relatively low numbers of unique peptides identified in NMRI plasma was too low to predict protein abundance. The finding that the original CREP2 [18] possessed a sequence gap compared to our CREP2.1 protein suggests that sequence variation exists for this gene. In this case, isoforms may differ in mRNA stability or half-life, or may be subject to differential regulation.

Results of our plasma affinity pull-down experiments extend the initial findings of previous schistosome-snail protein-protein interactome studies [15,24] to include, not only multiple VIgL domain lectins with sporocyst/LTP binding activity, but other immune-related proteins. Although not previously reported from plasma, lipopolysaccharide (LPS) binding protein and the matrix adhesion protein dermatopontin were identified in the albumen gland [46,47] and hemocytes [48,49] of *B*. *glabrata*. Presumably their presence in plasma is due to active secretion from these, or other, cellular sources where they carry out their antibacterial/adhesive activities. The binding of HSP70 to larval matrices is an important, although not surprising, finding. Expression of HSPs, including HSP70, has been correlated with both up-or down-modulation of resistance in *B*. *glabrata* in response to schistosome infections, apparently through stress-induced mechanisms [31, 32, 50]. It should be emphasized that, although there is clearly a strong correlation between stress protein responses and infection status, a functional linkage (cause and effect) has yet to be established. This would include the role of plasma HSP70 in mediating possible immune interactions.

As previously mentioned, TEPs (including the iTEP and CD109 subgroups [51]) were highly enriched in the plasma of both snail strains eluted from affinity columns supporting their putative role as snail pattern recognition receptors (PRR). TEP-mediated larval recognition may be through an opsonization mechanism [52,53] whereby encapsulating hemocytes either react directly with larval-bound TEPs or first with other PRRs (e.g., circulating VIgL lectins) creating molecular complexes that are recognized by hemocytes [24]. A third TEP-like protein, identified as complement C4-like, was exclusively found in eluates of NMRI plasma. However, follow-up sequence comparison showed it to be essentially identical to the TEPs and was lumped into that group.

Another group of immune proteins with larval-binding activity included a cytolysin (biomphalysin) and several Zn-dependent metalloproteinases (Zn-MPs) related to the ADAM (A Disintegrin And Metalloproteinase) family subgroup. Biomphalysin, a pore-forming aerolysin, is known to be cytolytic to sporocysts [54], so its capacity for bind sporocyst membrane proteins would not be unexpected. Consistent with our present finding, an aerolysin-related protein (probable biomphalysin) also was identified previously in a sporocyst extract-plasma co-precipitation assay [24]. Zn-MPs of the ADAM family are matrix metalloproteinases that function in tissue morphogenesis/remodeling and inflammation [55]. Proteomic analysis of larval binding plasma proteins revealed three Zn-dependent ADAM-related proteins: an ADAM with a thrombospondin motif (ADAM-TS) and two other Zn-MP identified in NCBI as uncharacterized proteins. The ADAM-TS, known to use collagens and other connective tissue/matrix proteins as substrates [56], appeared to be differentially expressed at both the plasma protein and transcript levels in the susceptible NMRI snails. Functionally, a relatively high level of circulating ADAM-TS may be advantageous to invading schistosome larvae by serving to degrade endogenous host matrix proteins, thereby facilitating larval movement through snail tissues. The recent demonstration that venom allergen-like (VAL) proteins released from transforming *S*. *mansoni* miracidia induces host cell production/activity of matrix-MP enzymes [57] also supports the notion that high constitutive levels of host plasma ADAM-TS may actually be contributing to larval survival in susceptible *B*. *glabrata* strains.

Among other metal-dependent proteins with putative immune function, the predicted GATA Zn-finger domain protein was of special interest because it appears to be related to Cu-Zn superoxide dismutases (Cu-Zn SOD) and, based on the high unique peptide count, may be occurring in abundance in snail plasma. Previous studies have shown that reactive oxygen species (ROS), mainly H_2_O_2_, represent the primary effector molecules involved in the killing *S*. *mansoni* sporocysts [58], and that allele-specific expression of Cu-Zn SOD, enzymes responsible for H_2_O_2_ production, is linked to the resistance phenotype in *B*. *glabrata* [59,60]. Although the larval-binding SOD-like protein found in the present study is not related to previously identified SOD1, its constitutive high levels in snails of both strains suggest a possible role in innate responses to other peroxide-sensitive pathogens.

Finally, our finding of other non-immune sporocyst-reactive plasma proteins consistently represented in plasma pull-down samples also provide hints as to how primary sporocysts establish infections early in their development. For example, hemoglobins types 1 and 2 were highly overrepresented in our samples, which was not unexpected given that hemoglobins have been shown, not only to be the most abundant protein in snail plasma [61], but are capable of binding to the tegumental surface of sporocysts [62, 63]. It is speculated that tegumental binding of hemoglobin may be functioning as a parasite-protective molecular disguise for avoiding immune recognition [4, 64]. Other identified proteins known to engage in protein-protein (e.g., actins, tubulins, collagen) or protein-carbohydrate (amylase) interactions also may be predicted to bind larval proteins, and therefore, were not unexpected in our pull-down assays. It should also be noted that the finding of several nonimmune plasma proteins that were not expected to be in high abundance in plasma were enriched in, and recovered from, larval affinity columns. These include an acetylcholine binding protein, balbiani ring protein3, and apolipophorin protein. Although we cannot rule out nonspecific binding by some plasma proteins, the disproportionate enrichment of these proteins suggests that protein binding to columns was not due entirely to nonspecific column interactions, but reflects preferential binding to select larval proteins. The differential binding of several proteins (e.g. the abundant tubulins) almost exclusively to Mem columns also supports tissue-specific selectivity of the experimental system.

In summary, using a multiple protein-protein interaction experimental design, we have provided an expanded and detailed picture of the plasma proteins of susceptible (NMRI) and resistant (BS-90) strains of *B*. *glabrata* that are capable of binding membrane and secreted/excreted proteins of early developing *S*. *mansoni* larvae. Follow-up analyses of reactive plasma proteins using a proteogenomic approach has provided valuable insights into the identity and function of proteins, especially those comprising the innate immune system including diverse members of the VIgL domain gene family. One major advantage of this approach is that it does not require *a priori* knowledge of open reading frames or a completely annotated genome in order to make putative gene or protein identifications. Recent studies have demonstrated the value of this approach in protein discovery efforts and the annotating of genomes of various organisms [65,66]. Because the initial aim of this investigation was to establish a proteomic baseline for comparing the interactions between several complex protein systems, only plasmas from uninfected *B*. *glabrata* strains were used. Future studies that overlay the effects of larval infections on plasma protein profiles clearly are needed. Such investigations will not only provide a more comprehensive picture of the molecular “interactome” of larval schistosomes and their snail host, but will shed light on specific host and parasite factors driving compatibility polymorphism [25].

## Materials and methods

### Ethics statement

All experimental protocols involving mice used in the course of this study were reviewed and approved by the Institutional Animal Care and Use Committee (IACUC) at the University of Wisconsin-Madison under Animal Welfare Assurance No. A3368-01. Protocols are in compliance with OLAW regulations of the National Institutes of Health (NIH).

### Preparation of *B*. *glabrata* plasma and cultured *S*. *mansoni* sporocysts

Hemolymph was obtained from inbred *S*. *mansoni*-susceptible and–resistant *B*. *glabrata* snails (NMRI and BS-90 strains, respectively) by the headfoot retraction method [67]. Upon collection, hemolymph from ~50 snails of each strain (8–12 mm in diameter) were dispensed into multiple 1.5 ml microcentrifuge tubes containing cold Chernin’s balanced salt solution (CBSS) [68] creating a 1:1 dilution of CBSS:hemolymph. Tubes were centrifuged at 1000 rpm (Eppendorf) for 10 min at 4°C to pellet hemocytes followed by removal and pooling of the cell-free hemolymph (plasma) for each strain. Plasma samples were used either immediately or aliquoted and stored at 4°C for use within 4 days.

Sporocysts of the NMRI strain of *S*. *mansoni* were obtained by cultivation of axenically isolated miracidia as described by Yoshino and Laursen [69]. Briefly, eggs were isolated from the livers of mice infected with *S*. *mansoni* 7 wk prior to necropsy. Miracidia were transferred to 15-mL test tubes, immobilized on ice to concentrate, and distributed into wells of a 24-well tissue culture plate containing sterile CBSS (pH 7.4) supplemented with Pen-Strep antibiotics and trehalose (1 g/mL). After cultivation for 24 h at 26°C under normoxic conditions, the CBSS was removed, filtered through a 0.45 μm syringe filter to remove stray parasites and any cellular debris. The larval-conditioned CBSS medium was then transferred to an ultrafiltration unit fitted with a 3 kDa-cutoff filter (Millipore Corp., Billerica, MA) and centrifuged at 1680 rpm for 80 min at 4°C. Protein concentrations were estimated using a Nanodrop spectrophotometer (ND-1000, NanoDrop), followed by addition of protease inhibitors (Calbiochem, EMD Millipore) and storage at -20°C. This medium served as the source of larval proteins released during miracidium-to-sporocyst transformation (larval transformation proteins or LTP). *In vitro* transformed primary sporocysts were maintained in CBSS culture for an additional 24 h at which time larvae were transferred to 1.5 mL microcentrifuge tubes and washed 3x in CBSS by sedimentation to eliminate ciliated plates and cell debris prior to biotinylation.

### Biotinylation of sporocyst tegument/LTP proteins

Live 2-day cultured *S*. *mansoni* primary sporocysts, obtained as described above, were prelabeled with NHS-biotin (20 mM in CBSS, 10 min at 4°C), blocked in 0.2M glycine in CBSS, and washed 3x in cold CBSS. Biotinylated sporocysts were pelleted and immediately subjected to serial fractionation using a ProteoExtract Subcellular Proteome Extraction Kit (Merck KGaA, Darmstadt, Germany). Briefly, sporocysts were suspended in wash buffer (kit provided) for 10 min at 4°C, centrifuged for removal of buffer and then serially extracted in four extraction buffers that yielded enriched cytosolic, membrane, nuclear and cytoskeletal samples (**S1 Fig**) according to the manufacture’s protocol with modifications [70].

Similarly, concentrated LTP samples harvested from 24-h larval cultures were pooled for preparation of the biotinylated LTP. The amount of biotin solution added to the LTP sample was calculated as recommended by the manufacturer (EZ-link NHS-PEG4-Biotin, Thermo Scientific, Rockford, IL). LTP samples (2–10 μg/μL) were combined with ≥12-fold molar excess of biotin and incubated at 22°C for 20 min. Nonreacted biotin was removed by dialysis against Tris-buffered saline (TBS; 25 mM Tris-HCl, 0.15M NaCl, pH 7.0) for 2 h. Dialysis was repeated with buffer changes twice more, with the last dialysis being performed overnight at 4°C.

### Affinity column construction

Following protein concentration determination, the biotinylated membrane-enriched fraction (designated Mem) and LTP were introduced to mini-columns packed with streptavidin-coupled agarose beads (Biotinylated Protein Interaction Pull-Down Kit, Pierce, Rockford, IL). After overnight incubation at 4°C, unbound proteins were removed by washing the columns with 2 mL of TBS, followed by adding 250 μL of biotin blocking buffer to each column for 5 min. After blocking, columns were washed with 250 μL of TBS buffer and used immediately, or stored at 4°C and used within 2 days. For affinity separation of snail plasma proteins, 150 μL of plasma (500 μg/150 μL) from NMRI and BS-90 *B*. *glabrata* were applied to separate Streptavidin-Mem or -LTP affinity columns, allowed to incubate with the affinity matrix overnight at 4°C and washed 5x with 250 μL of wash buffer. Bound plasma proteins were eluted with 250 μL of sodium acetate elution buffer, pH 2.8. Eluted fractions were immediately neutralized by addition of 10 μL of 1M Tris buffer (pH 8.5) and aliquots subjected to SDS-PAGE separation and silver staining. After washing columns with TBS, blocking buffer was again introduced for 5 min, washed with TBS and re-used immediately, or stored at 4°C and used within 2 days. Eluted plasma samples that had been obtained at a single bleeding of NMRI and BS-90 snails and applied to a Mem or LTP affinity column represented a single biological replicate that included four separate samples: NMRI/Mem, NMRI/LTP, BS-90/Mem and BS-90/LTP. Proteomic analyses were performed on plasma elutes from 3 LTP and 4 Mem replicates.

In parallel with plasma-Mem and -LTP test columns, three control columns were simultaneously run: (1) biotinylated Mem/LTP-Streptavidin beads with no added plasma (buffer control), (2) Streptavidin beads alone + NMRI plasma (NMRI plasma control) and (3) Streptavidin beads alone + BS-90 plasma (BS-90 plasma control). Following treatments as described above, control columns were washed with blocking buffer, eluted with acetate elution buffer, and, after neutralization, each fraction was analyzed for spurious protein binding by SDS-PAGE/silver staining.

### Proteomic analysis of sporocyst membrane/LTP-binding plasma proteins

BS-90 and NMRI plasma eluates from Mem and LTP affinity columns were collected, their protein concentrations determined (ND-1000, NanoDrop Technologies, Wilmington, DE), followed by subjecting equal concentrations to enzymatic “in liquid” digestion. Enzymatic digestions and mass spectrometric analyses were performed at the Mass Spectrometry Facility (Biotechnology Center, University of Wisconsin-Madison). In short, soluble proteins in buffer were first precipitated in 15% (final concentration) TCA, incubated overnight at 4°C, and centrifuged for 10 min at 16,000xg. Resulting pellets were washed three times with ice-cold acetone, followed by re-solubilization of proteins in 10 μL of denaturation solution (8M Urea in 100mM NH_4_HCO_3_ pH 8.2) for 10 min. For tryptic digestion, denatured proteins were diluted to 50 μL final volume with 2.5 μL of 25mM DTT, 2.5 μL of MeOH, 27.25 μL of 25mM NH_4_HCO_3_, 0.25 μL of 1M Tris-HCl, 5 μL of 500mM NH_4_HCO_3_ and 2.5 μL trypsin solution (100 ng/μL in 25mM NH_4_HCO_3_) (Trypsin Gold, Promega Corp, Madison, WI). Digestions were conducted in two stages; first overnight incubation at 37°C, followed by addition of 1 μL of trypsin solution and a second incubation for 2 hr at 42°C. Reactions were terminated by acidification with 2.5% trifluoroacetic acid (TFA) to 0.3% final concentration. Peptides generated from tryptic digestion were concentrated on C18 OMIX tips (Varian Inc., Lake Forest, CA), eluted, dried and then reconstituted in 20 μL of 0.05% TFA. A volume of 8 μL was loaded for nanoLC-MS/MS analysis.

### NanoLC-MS/MS analyses

Peptides were analyzed by nanoLC-MS/MS using the Agilent 1100 nanoflow system (Agilent Technologies) connected to a hybrid linear ion trap-orbitrap mass spectrometer (LTQ-Orbitrap XL, Thermo Fisher Scientific) equipped with a nanoelectrospray ion source. HPLC was performed using an in-house fabricated 12-cm C18 column packed with 5μm C18 spherical silica particles (Column Engineering, Ontario, CA) and laser pulled tip (P-2000, Sutter Instrument) using 360μm x 75μm fused silica tubing. Sample loading (8μL) and desalting were done at 10 μL/min using a trapping column in line with the autosampler (Zorbax 300SB-C18, 5μM, 5x0.3mm, Agilent Technologies). Peptide elution used solvents comprised of 0.1M acetic acid in water (solvent A) and 0.1M acetic acid, 95% acetonitrile in water (solvent B). The gradient consisted of a 20 min loading and desalting period with column equilibration at 0% solvent B, an increase to 40% B over 200 min, ramp to 60% B over 20 min, increase to 100% B in 5 min and hold for 3 min. The column was then re-equilibrated at 1% B for 30 min. The flow rate for peptide elution and re-equilibration was at 200 nL/min. The LTQ-Orbitrap was set to acquire MS/MS spectra in data-dependent mode as follows: MS survey scans from m/z 300 to 2000 were collected in centroid mode at a resolving power of 100,000. MS/MS spectra were collected on the 5 most-abundant signals in each survey scan. Dynamic exclusion was employed to increase dynamic range and maximize peptide identifications. This feature excluded precursors up to 0.55 m/z below and 1.05 m/z above previously selected precursors. Precursors remained on the exclusion list for 40 sec. Singly-charged ions and ions for which the charge state could not be assigned were rejected from consideration for MS/MS analysis.

### Bioinformatic analyses

The original raw MS/MS peptide datasets were initially searched against the six-frame translation of the *Biomphalaria glabrata* genome super-contig sequence database (899,428 sequences; http://biology.unm.edu/biomphalaria-genome/; version 4.3) and subsequently, against the corresponding scaffold translations found in the *B*. *glabrata* genome assembly in VectorBase (https://www.vectorbase.org/organisms/biomphalaria-glabrata). All queries incorporated an in-house *Mascot* search engine, with Met oxidation and Asp/Glu deamidation as variable modifications. Peptide mass tolerance was set at 15 ppm and fragment mass was set at 0.6 Da. Putative ORFs containing exact peptide matches were subjected to pBLAST searches against the non-redundant NCBI protein database to obtain initial protein identifications. With continuing assembly and annotation of the *B*. *glabrata* genome in VectorBase [71], we re-analyzed the entire proteomic dataset using Mascot and Sequest against the *B*. *glabrata* NCBInr database. For assignment of final protein identities, Protein Prophet algorithm’s protein identification criteria [26] were established at > 99.0% probability to achieve an FDR < 1.0% and to contain at least 2 identified peptides (with > 93.0% probability to achieve an FDR < 1.0% by the Scaffold Local FDR algorithm). The complete raw dataset used in bioinformatics analyses of the MS data are available in the PRIDE file submitted to proteomeXchange (Ref#1-20160909-104700). Protein domains were identified using SMART (Simple Modular Architecture Research Tool) using normal mode and including outlier homologues and PFAM domains; http://smart.embl-heidelberg.de/smart/set_mode.cgi?NORMAL=1). Multiple-sequence alignments were accomplished using Clustal Omega (http://www.ebi.ac.uk/Tools/msa/clustalo/) [72].

Analyses of MS-generated plasma peptides/spectra data revealed considerable variability in identified proteins/peptides between replicate experiments in the various matrix/plasma combinations. This replicate-to-replicate variability likely was due to a combination of factors including inherent variability in both quantity and quality of individual proteins in plasma samples, the relatively small subset of enriched proteins recovered from affinity columns combined with the often small quantities of individual eluted proteins, variability in the construction of affinity matrices (column-to-column variation) and finally, limited run-to-run reproducibility inherent in mass spec analyses of complex protein samples, especially for low abundance proteins [73]. Due to these limitations we undertook a more qualitative comparison of peptide presence/absence in combined replicate datasets to provide an overall assessment of plasma proteins with larval-binding potential. In some cases, an estimate of relative protein abundance, based on unique peptide numbers, was used to identified high abundance proteins or those with potential for differential binding between snail strains or larval tissue sources.

### cDNA sequencing and transcript expression analyses of selected immune-related proteins

In order to corroborate initial findings of potential differential binding of immune-related proteins from NMRI and BS-90 snail strains, PCR were performed targeting FREP12, GREP, CREP2, and ADAM-TS. Protein selection was based on an arbitrary criterion of possessing a minimum of four unique peptide sequences exclusive to one snail strain (with the exception of the GREP) in order to increase the likelihood of true differential protein expression. Specific forward and reverse PCR primers were designed to cover the coding regions of each transcript (**S7 Table**), followed by PCR amplification of whole body–derived cDNA of individual NMRI and BS-90 *B*. *glabrata* snails (detailed in next section). A positive PCR loading control consisted of primers designed to amplify a similar sized amplicon of *B*. *glabrata* α-actinin, known to be similarly expressed in both snail strains. PCR products from test and control reactions were simultaneously run on a 1.2% agarose gel, stained with ethidium bromide, and visualized under UV light (GelDoc-It Imaging System, UVP LLC, Upland, CA).

#### RNA extraction and cDNA synthesis

Ten NMRI and ten BS90 *B*. *glabrata* snails ranging from 7 to 9 mm in diameter were used to extract whole snail total RNA. RNA from each snail was individually extracted using Trizol reagent (Invitrogen) following the manufacturer’s protocol. Isolated total RNA samples were resuspended in nuclease-free water, quantified using a Nanodrop spectrophotometer (ND-1000), diluted to 180 ng/ μL and treated with DNase (Turbo-DNA-free kit; Life Technologies) at 37°C for 30 min. After Dnase treatment, 360 ng of total RNA/sample were used to synthesize cDNA using OligodT and random primers. Complementary DNA was synthesized using GoScript cDNA synthesis kit (Promega, Madison, WI), followed by aliquoting and storage of the resulting cDNA of both snail strains at -20°C prior to PCR analysis.

#### Transcript amplification and sequencing

Forty ng of snail cDNA were incorporated into PCRs using the standard protocol from Flexi Taq (Promega). Reactions were performed at the same time on all 20 snail samples according to the manufacturer’s recommendations using a Tm of 52–53°C for all transcripts and α-actinin (loading control) amplifications with an extension step of 1min/1kb min at 72°C for 40 cycles. Primer sequences, amplicon sizes and specific Tm are listed in S7 Table. Following PCR, samples were run on a 1.2% agarose gel in parallel with a DNA marker (Perfect DNA Markers 0.1-12kb, Novagen) and imaged using a GelDoc-It Imaging System (UVP). In addition, amplicons for GREP, CREP2 and FREP12 were gel-excised from three individual NMRI and BS-90 lanes, followed by purification of DNA using a Qiaquick gel extraction kit (Qiagen), quantification (Nanodrop) and direct sequencing of samples using BigDye Terminator v. 3.1 (Applied Biosystems) using standard protocol and cycle conditions. Sequencing reactions were purified using CleanSeq magnetic bead (Beckman Coulter, Agencourt Bioscience), after which samples were resuspended in 50 μL of ddH_2_O and sent to the University of Wisconsin Biotechnology Center for further sequencing.

## Supporting information

S1 Fig **Silver stained SDS-PAGE-fractionated *Biomphalaria glabrata* plasma proteins eluted from biotinylated sporocyst membrane (bMem) (A) and biotinylated larval transformation protein (bLTP) (B) affinity columns.** Plasma from susceptible NMRI (NM) or resistant BS-90 (BS) *B*. *glabrata* strains was introduced into streptavidin affinity columns pre-loaded with larval bMem, bLTP or with no larval proteins (Neg; control column). Following extensive washing to remove unbound proteins, eluted plasma proteins from bMem, bLTP or no protein control (Neg) columns were collected and subjected to SDS-PAGE analysis (Lanes 1–3). Larval protein columns lacking snail plasma (TBS; Lane 3) yielded no detectable eluted larval proteins. In addition, to show the extent of potential nonspecific binding to the affinity matrix, plasma from both strains was introduced into naked streptavidin columns (lacking biotinylated larval proteins) and eluates assessed by SDS-PAGE (Lanes 4 and 5). Overall, there was the strong enrichment of plasma proteins eluted from larval protein affinity columns.
(TIF)

S1 Table Summary of the number of unique peptide sequences and identified proteins recovered from NMRI and BS-90 *Biomphalaria glabrata* plasma samples following elution from Mem and LTP affinity columns. Total numbers of unique peptides and plasma proteins identified from all sample replicates combined were similar between snail strains and sporocyst membrane (Mem) and larval transformation protein (LTP) affinity matrices.
(PDF)

S2 Table Listing of predicted non-immune *Biomphalaria glabrata* plasma proteins eluted from Mem and LTP affinity columns identified in VectorBase. Protein identifications (Sequence ID) were determined using Mascot and Sequest against the current *B*. *glabrata* annotated NCBInr database (see Materials and Methods for details).
(PDF)

S3 Table Protein sequences, identified domains and genomic locations in VectorBase of NMRI and BS-90 *Biomphalaria glabrata* VIgL domain-containing plasma proteins eluted from sporocyst membrane-enriched (Mem) affinity columns. Protein identifications (Sequence ID) were determined using Mascot and Sequest against the current *B*. *glabrata* annotated NCBInr database. The numbers following the Scaffold idenfications (:xxx-yyy) designate the location of the coding gene sequence within the designated scaffold. Peptide sequences in red represent the original peptides identified in the tandem MS analyses of plasma proteins.
(PDF)

S4 Table Protein sequences, identified domains and genomic locations in VectorBase of NMRI and BS-90 *Biomphalaria glabrata* VIgL domain-containing plasma proteins eluted from larval transformation protein (LTP) affinity columns. Protein identifications (Sequence ID) were determined using Mascot and Sequest against the current *B*. *glabrata* annotated NCBInr database. The numbers following the Scaffold idenfications (:xxx-yyy) designate the location of the coding gene sequence within the designated scaffold. Peptide sequences in red represent the original peptides identified in the tandem MS analyses of plasma proteins.
(PDF)

S5 Table Protein sequences, identified domains and genomic locations in VectorBase of other immune-related NMRI and BS-90 *Biomphalaria glabrata* plasma proteins eluted from sporocyst membrane-enriched fraction (Mem) affinity columns. Protein identifications (Sequence ID) were determined using Mascot and Sequest against the current *B*. *glabrata* annotated NCBInr database. The numbers following the Scaffold idenfications (:xxx-yyy) designate the location of the coding gene sequence within the designated scaffold. Peptide sequences in red represent the original peptides identified in the tandem MS analyses of plasma proteins.
(PDF)

S6 Table Protein sequences, identified domains and genomic locations in VectorBase of other immune-related NMRI and BS-90 *Biomphalaria glabrata* plasma proteins eluted from larval transformation protein (LTP) affinity columns. Protein identifications (Sequence ID) were determined using Mascot and Sequest against the current *B*. *glabrata* annotated NCBInr database. The numbers following the Scaffold idenfications (:xxx-yyy) designate the location of the coding gene sequence within the designated scaffold. Peptide sequences in red represent the original peptides identified in the tandem MS analyses of plasma proteins.
(PDF)

S7 Table PCR primer sequences used to amplify transcripts encoding GREP, CREP2, FREP12, and ADAM-TS from cDNA synthesized from isolated *Biomphalaria glabrata* whole body RNA. (PDF)
