# Supplementary figures and images for: Proteomic analysis of Biomphalaria glabrata plasma proteins with binding affinity to those expressed by early developing larval Schistosoma mansoni
Source: PLoS Pathog. 2017 May 16;13(5):e1006081. doi: 10.1371/journal.ppat.1006081 (PMC5433772; doi:10.1371/journal.ppat.1006081)

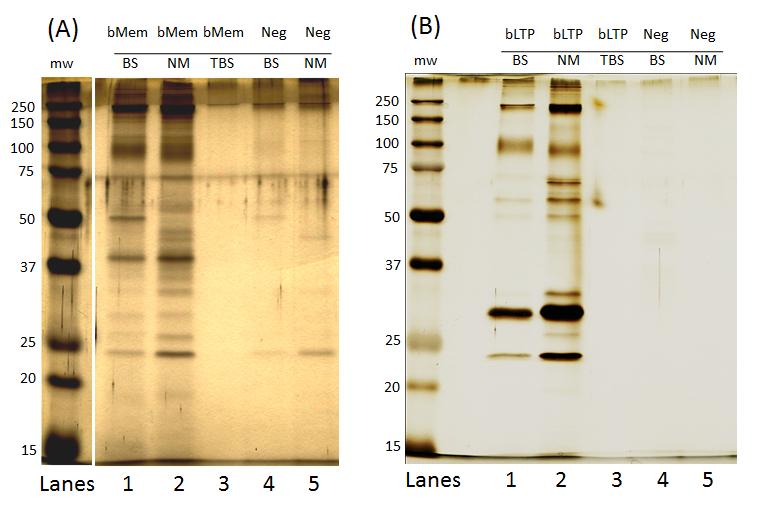

Supplement: S1 Fig — Silver stained SDS-PAGE-fractionated Biomphalaria glabrata plasma proteins eluted from biotinylated sporocyst membrane (bMem) (A) and biotinylated larval transformation protein (bLTP) (B) affinity columns. Plasma from susceptible NMRI (NM) or resistant BS-90 (BS) B. glabrata strains was introduced into streptavidin affinity columns pre-loaded with larval bMem, bLTP or with no larval proteins (Neg; control column). Following extensive washing to remove unbound proteins, eluted plasma proteins from bMem, bLTP or no protein control (Neg) columns were collected and subjected to SDS-PAGE analysis (Lanes 1–3). Larval protein columns lacking snail plasma (TBS; Lane 3) yielded no detectable eluted larval proteins. In addition, to show the extent of potential nonspecific binding to the affinity matrix, plasma from both strains was introduced into naked streptavidin columns (lacking biotinylated larval proteins) and eluates assessed by SDS-PAGE (Lanes 4 and 5). Overall, there was the strong enrichment of plasma proteins eluted from larval protein affinity columns. (TIF) [file ppat.1006081.s001.tif]
