# Supplementary material for: Proteomic analysis of Biomphalaria glabrata plasma proteins with binding affinity to those expressed by early developing larval Schistosoma mansoni
Source: PLoS Pathog. 2017 May 16;13(5):e1006081. doi: 10.1371/journal.ppat.1006081 (PMC5433772; doi:10.1371/journal.ppat.1006081)
Supplement: S1 Table — Total numbers of unique peptides and plasma proteins identified from all sample replicates combined were similar between snail strains and sporocyst membrane (Mem) and larval transformation protein (LTP) affinity matrices. (PDF) [file ppat.1006081.s002.pdf]

S1 Table. Summary of the number of unique peptide sequences and identified proteins recovered from NMRI and BS-90 *B. glabrata* plasma samples following elution from Mem and LTP affinity columns

Mem (Sporocyst Membrane Proteins)		
	**NMRI**	**BS-90**
# unique peptides	564	480
# protein ID	58	51
# unidentified proteins	5	2
**LTP (Larval Transformation Proteins)**		
	**NMRI**	**BS-90**
# unique peptides	531	451
# protein ID	45	36
# unidentified proteins	3	1
