# Supplementary material for: Proteomic analysis of Biomphalaria glabrata plasma proteins with binding affinity to those expressed by early developing larval Schistosoma mansoni
Source: PLoS Pathog. 2017 May 16;13(5):e1006081. doi: 10.1371/journal.ppat.1006081 (PMC5433772; doi:10.1371/journal.ppat.1006081)
Supplement: S2 Table — Protein identifications (Sequence ID) were determined using Mascot and Sequest against the current B. glabrata annotated NCBInr database (see Materials and Methods for details). (PDF) [file ppat.1006081.s003.pdf]

S2 Table. Listing of predicted non-immune *B. glabrata* plasma proteins eluted from Mem and LTP affinity columns identified in VectorBase.

Protein Identification	Unique Peptide Count				VectorBase Scaffold
	BS-90		NMRI		
	Mem	LTP	Mem	LTP	
**hemoglobin type 1** [*Biomphalaria glabrata*] ; Sequence ID: CAJ44466.1; Identities: 100%	108	128	143	128	LGUN_random_Scaffold5741:1590-15334; minus strand
**PREDICTED: uncharacterized protein LOC106051763** [*Biomphalaria glabrata*]; Sequence ID: XP_013062423.1; Identities: 100%<br>**haemoglobin type 1** [*B. glabrata*]; Sequence ID: CAJ44466.1; Identities: 76%	12	11	6	7	LG17_random_Scaffold37: 105709-105474; 103671-103436; 101378-101137; 99539-99300; 91005-90767; 88372-88137; 23383-23148; 21154-20907; 19765-19530; 16554-16319; minus strand
**PREDICTED: uncharacterized protein LOC106051722** [*Biomphalaria glabrata*]; Sequence ID: XP_013062383.1 ; Identities: 100%<br>**haemoglobin type 1** [*B. glabrata*] Sequence ID: CAJ44466.1; Identities: 87%	10	5	3	5	LG17_random_Scaffold37:10,424-11,812; minus strand; BGLB000834-PA
**PREDICTED: uncharacterized protein LOC106077292** [*Biomphalaria glabrata*]; Sequence ID: XP_010393514.1; Identities: 100%<br>**haemoglobin type 1** *[B. glabrata*]; Sequence ID: CAJ44466.1; Identities: 65%	4	6	6	5	LGUN_random_Scaffold558: 30767-30464; 24999-24708; 20689-20454; 16076-15841; 13539-13304; 10586-10351; 7603-7362; 2289-2054; 1556-590; minus strand
**PREDICTED: uncharacterized protein LOC106078048** **[***Biomphalaria glabrata*]; Sequence ID: XP_013094243.1; Identities: 100%<br>**haemoglobin type 1** [*B. glabrata*]; Sequence ID: CAJ44466.1; Identities: 60%	3	6	8	8	LGUN_random_Scaffold5741: 12584-12495; 11353-11244; 9616-9322; 8037-7419; minus strand

**PREDICTED: uncharacterized protein LOC106050210,** partial [*Biomphalaria glabrata*]; Sequence ID: XP_013060614.1; Identities: 100% **haemoglobin type 1** [*B. glabrata*]; Sequence ID: CAJ44466.1 ; Identities: 58%	5	8	6	7	LGUN_random_Scaffold7018: 3821-3568; 371-76; minus strand
**hemoglobin type 2** [*Biomphalaria glabrata*] Sequence ID: CAJ44467.1; Identities: 100%	81	102	108	108	LGUN__random_Scaffold 8143:20,982-37,676; minus strand. BGLB013430
**PREDICTED: uncharacterized protein LOC106051916** [*Biomphalaria glabrata*]; Sequence ID: XP_013062584.1; Identities: 100% **haemoglobin type 2** [*B. glabrata*]; Sequence ID: CAJ44467.1; Identities: 98%	7	7	10	6	LGUN_random_Scaffold8143:20982:37676:-1 gene:BGLB013430; 97%; plus strand
**PREDICTED: uncharacterized protein LOC106050211,** partial [*Biomphalaria glabrata*]; Sequence ID:XP_013060615.1 ; Identities: 100% **haemoglobin type 2** [*B. glabrata*]; Sequence ID: CAJ44467.1; Identities: 96%	3	3	3	2	LGUN_random_Scaffold7018:4623:18026:1|gene:BGLB012730 87%; plus strand
**PREDICTED: uncharacterized protein LOC106051747,** partial [*Biomphalaria glabrata*];Sequence ID: XP_013062404.1; Identities: 100% **haemoglobin type 2** [*B. glabrata*]; Sequence ID: CAJ44467.1; Identities: 86%	6	7	6	4	LGUN_random_Scaffold8143:20982:37676:-1|gene:BGLB013430; 86%; plus strand
**Chain A, acetylcholine-binding protein Type 2 (Bgachbp2)** [*Biomphalaria glabrata*]; Sequence ID: 4AOE_A ; Identities: 100%	10	15	9	17	LG29_random_Scaffold14:635533-635416; 634747-634508; 632875-632202; minus strand
**PREDICTED: acetylcholine-binding protein-like isoform X1** [*Biomphalaria glabrata*]; Sequence ID: XP_ 013067426.1; Identities: 100%	4	10	4	9	LG29_random_Scaffold14:635533-635416; 634747-634508; 632875-632202; minus strand
**PREDICTED: actin-2-like** [*Biomphalaria glabrata*]; Sequence ID: XP_013083929.1; Identities: 100%	1	0	2	2	LGUN_random_Scaffold29:432435:441953:1|gene:BGLB008299; plus strand

**hypothetical protein LOTGIDRAFT_193218** [*Lottia gigantea*]; Sequence ID: XP_009060604.1; Identities: 100%<br>**PREDICTED: actin** [*Crassostrea brasiliana*]**;** Sequence ID: ANB49595.1; Identities: 99%	5	2	7	4	LGUN_random_Scaffold10528:12872:19460:1|gene:BGLB00391; plus strand
**PREDICTED: actin, alpha skeletal muscle** [*Biomphalaria glabrata*]; Sequence ID: XP_013073050.1; Identities: 95%	2	0	1	0	LGUN_random_Scaffold29:432435:441953:1|gene:BGLB008299 plus strand
**PREDICTED: tubulin alpha-1A chain-like, partial** [*Biomphalaria glabrata*]; Sequence ID: XP_013091074.1; Expect: 0.0; Identities: 100%	11	0	8	1	LGUN_random_Scaffold5085:5474-17809:1|gene:BGLB010861 (100%); plus strand
**PREDICTED: tubulin alpha-3 chain-like, partial** [*Biomphalaria glabrata*]; Sequence ID: XP_013095645.1 ; Identities: 100%	4	0	1	1	LGUN_random_Scaffold6191:6220-9108:-1|gene:BGLB012164; plus strand
**PREDICTED: tubulin beta-4B chain-like** [*Biomphalaria glabrata*]; Sequence ID: XP_013097130.1; Identities: 100%	2	0	1	0	LGUN_random_Scaffold6940:15330-18731:1|gene:BGLB012690 (100%); plus strand
**PREDICTED: tubulin beta-4B chain-like** [*Biomphalaria glabrata*]; Sequence ID: XP_013085141.1; Identities: 100%	16	0	13	0	LGUN_random_Scaffold3175:23130:28038:1|gene:BGLB008716 (100%); plus strand
**PREDICTED: collagen alpha-1(XII) chain-like, partial** [*Biomphalaria glabrata*]; Sequence ID: XP_013088320.1 ; Identities: 100%	6	0	5	2	LGUN_random_Scaffold409: 210568-210376; 208117-207998; 203161-202925; 200670-200506; 198286-198104; 195116 194857; minus strand
**PREDICTED: collagen alpha-5(VI) chain-like** [*Biomphalaria glabrata*]; Sequence ID: XP_013079619.1 ; Identities: 100%	0	0	4	7	LGUN_random_Scaffold2059:1: 17949-18112; 19250-19482; 21462-21627; 22126-22308; 31136-31259; 33936-34068; 36698-36930; 39753-39921; 41246-41428; plus strand
**PREDICTED: uncharacterized protein LOC106073418** [*Biomphalaria glabrata*]; Sequence ID: XP_013089410.1; Identities: 100%<br>**PREDICTED: collagen alpha-1(IX) chain-like, partial** [*B. glabrata*]; Sequence ID: XP_013071005.1; Identities: 70%	0	2	0	2	LGUN_random_Scaffold105369: 585-5; minus strand

**PREDICTED: balbiani ring protein 3-like** [*Biomphalaria glabrata*]; Sequence ID: XP_013096729.1 ; Identities: 100%	8	10	9	13	LGUN_random_Scaffold675: 24344-21090; minus strand
**PREDICTED: apolipophorin-like isoform X1** [*Biomphalaria glabrata*]; Sequence ID: XP_013082155.1 ; Identities: 100%	6	0	9	8	LG4_random_Scaffold2455: 41449-41188; 39683-39359; 36375-36110; 33330-33059; 32080-31815; 30252-25604; 18006-17780; 13866-13496; 6868-6117; minus strand
**PREDICTED: alpha-amylase-like** [*Biomphalaria glabrata*]; Sequence ID: XP_013062082.1 ; Identities: 100%	2	2	5	2	LGUN_random_Scaffold784:118162-121490:-1|gene:BGLB013250
**PREDICTED: ATP synthase subunit alpha, mitochondrial-like** [*Biomphalaria glabrata*]; Sequence ID: XP_013081788.1 ; Identities: 100%	4	0	3	0	LGUN_random_Scaffold2464:29420-32015:1|gene:BGLB007667
**PREDICTED: ATP synthase subunit beta, mitochondrial-like** [*Biomphalaria glabrata*]; Sequence ID: XP_013070512.1; Identities: 100%	6	0	6	0	LGUN_random_Scaffold10059:2817-7549:-1|gene:BGLB003755
**PREDICTED: sodium/potassium-transporting ATPase subunit alpha-like** [*Biomphalaria glabrata*]; Sequence ID: XP_013071165.1; Identities: 100%	5	0	4	0	LGUN_random_Scaffold107:64347-71294:-1|gene:BGLB004031
**PREDICTED: elongation factor 1-alpha** [*Biomphalaria glabrata*]; Sequence ID: XP_013067348.1; Identities: 100%	2	0	3	2	LG28i_random_Scaffold9449:4199-6514:-1|gene:BGLB001639
**PREDICTED: ubiquitin-40S ribosomal protein S27a** [*Biomphalaria glabrata*]**;** Sequence ID: XP_013063486.1; Identities: 100%	3	1	1	1	LGUN_random_Scaffold868:32636:33553:-1|gene:BGLB013769 LGUN_random_Scaffold868:1:102120:135986-35764; 32796-32596
**PREDICTED: tyrosine-protein phosphatase non-receptor type 23-like** [*Biomphalaria glabrata*]; Sequence ID: XP_013069839.1 ; Identities: 100%	1	2	1	1	LG9i_random_Scaffold325:325130:325976:1|gene:BGLB003636 LG9i_random_Scaffold325: 326111-326271; 325799-325962; 337767-337976; 339268-339428; 343490- 343683; 346069-348652; 351757-351981; 358275- 358477; 358667-360177

**Histone H4** [*Larimichthys crocea*]; Sequence ID: KKF20647.1 ; Identities: 100%	4	0	4	0	LGUN_random_Scaffold3683:39221:39397:1|gene:BGLB009366
**PREDICTED: adenosine deaminase CECR1-like, partial** [*Biomphalaria glabrata*]; Sequence ID: XP_013061189.1; Identities: 100%	0	0	3	0	LGUN_random_Scaffold7376:1:18597:1: 5703-5853; 6641-6818; 8965-9097; 13395-13527; 16836-16913 plus strand
**PREDICTED: clathrin heavy chain 1** [*Biomphalaria glabrata*]; Sequence ID: XP_013079752.1; Identities: 98%	3	0	0	0	LGUN_random_Scaffold2072:39208:58064:-1|gene:BGLB007033
**PREDICTED: V-type proton ATPase catalytic subunit A-like** [*Biomphalaria glabrata*]; Sequence ID: XP_013086122.1; Identities: 96%	2	0	0	0	LGUN_random_Scaffold3436:56868:63955:1|gene:BGLB009092
**PREDICTED: V-type proton ATPase subunit B** [*Biomphalaria glabrata*]; Sequence ID: XP_013087752.1; Identities: 99.8%	2	0	0	0	LG8_random_Scaffold135:164139:169688:1|gene:BGLB003477
**PREDICTED: putative tyrosinase-like protein tyr-1** [*Biomphalaria glabrata*]; Sequence ID: XP_013065893.1; Identities: 100%	2	0	0	0	LG20i_random_Scaffold3077:1:54679:1: 29371-29078; 27996-27830; 23386-23126; 22813-22597; 22259- 22108; 20827-20517; 19464-19267; 18992-18836; 18366-18277
**PREDICTED: ovalbumin-like isoform X2** [*Biomphalaria glabrata*]; Sequence ID: XP_013088530.1; Identities: 100%	2	0	1	0	LGUN_random_Scaffold4153:1:27349:1: 18675-18513; 17521-17299; 5618-5480; 4454-4305; 2924-2702
