# Supplementary material for: Proteomic analysis of Biomphalaria glabrata plasma proteins with binding affinity to those expressed by early developing larval Schistosoma mansoni
Source: PLoS Pathog. 2017 May 16;13(5):e1006081. doi: 10.1371/journal.ppat.1006081 (PMC5433772; doi:10.1371/journal.ppat.1006081)
Supplement: S3 Table — Protein identifications (Sequence ID) were determined using Mascot and Sequest against the current B. glabrata annotated NCBInr database. The numbers following the Scaffold idenfications (:xxx-yyy) designate the location of the coding gene sequence within the designated scaffold. Peptide sequences in red represent the original peptides identified in the tandem MS analyses of plasma proteins. (PDF) [file ppat.1006081.s004.pdf]

S3 Table. Protein sequences, identified domains and genomic locations in VectorBase of NMRI and BS-90 *Biomphalaria glabrata* VIgL domain-containing plasma proteins eluted from sporocyst membrane-enriched (Mem) affinity columns.

Protein ID	Scaffold No.	Predicted Protein Sequence
**FREP 2.28** [*Biomphalaria glabrata*] (100%); Sequence ID: ADK11410<br><br>Domains:<br>-Signal peptide: 1-20<br>-Ig: 38-148<br>-FBG: 181-382	500-1158:<br>LGUN_random_Scaffold563550:<br>640-1300; rev. frame; 1994bp<br>LGUN_random_Scaffold9301:<br>368-1028; 12407bp<br>199-502:<br>LGUN_random_Scaffold534902:<br>1310-1613; 1684bp	MASLPLRLVLLVSMVTLIRSSSWLNFTGNSETIRELIQPLKLTCTFQISKNDSENDSQVLFMSIYHETKRVIASISKYQPVAT<br>SLYPSVTKVQGQIYHSNESKDSYLQVTWTHPKLSESGKYFCLAHAWNSTSQNSVFDADITVNVIKSSTDDLAVALSYIQD<br>RLDKDGVDSIQISRASPTLPESCRDVISSEDRVVVTLASGLKVMCDTKTDGGGWIIFQRRINGYVDFYRGWKEYRDGFG<br>DYDIGEFYLGNENIFKLTSSKKYDLRIDLEFNNTKYFAFYTRFEILGEQDYYKLQIGGYSGNAGDALTNIHNDKFFSTYDKD<br>NDLGTSGSCAVTHKGAWWYRDCYDSSLNGKWGSDRINWSKLTGITKSVTFTEMKIREIELN
**FREP 3-2 precursor** [*Biomphalaria glabrata*] (100%); Sequence ID: AAK28656<br><br>Domains:<br>-Signal peptide: 1-18<br>-Ig: 28-134 / 183-301<br>-Coiled coil: 314-382<br>-FBG: 451-655	LG3_random_Scaffold2841:<br>18324:26062; BGLB000204;<br>253aa/ 655aa; 65764bp	MARLFLLFILCVFVVSLAGSELVIDVQPNVISPEITPQLVINCSITNNEVQTLDLIKSLTLSRYNETIREFDELIALDSLTLNLKQ<br>FVRFKYSQISFGNRYITLILHNPTQFDARIYKCNATGTNSEGANISLFAKKAVEYETNSTALIEEIRRIKKDENYCSFKKDDLS<br>DSKQRSRVYFSGSSDIIKERIEPLTLKCTFQVLKTDQNETSRLQSLYILHESKGVIAYVNKDQPVVTSLQGSNIQDVEGEIYD<br>NAIKDSYLQVTWSNLKHSESGKYFCEAHNKYSEGRIDKSSNMLTITVERPTFDDLVEAMHKLFTQVDGAKESLKAINQNI<br>KNINKDLDFKEQNITSIKQEVIRNQNNIQILSEDLNIKEQNLTSIKADLSTKQQTFLNIKEDVILNQQIIHKIKQDLNTYRHN<br>MSNIEEHLEVILTNLSTASIKVKNQTDEGSKMSYPPRKSCRDVNSTDERVVVTLTSGLKVMCDTKTDGGGWIIFQRRIN<br>GNVDFYRGWKEYRDGFGDYNIGEFYLGNENIYMLTSTGQYNLRIDLKYKNKAFFAQYSGFKILSEKEKYKLNIGAYSGNS<br>GDNFSSHNNAFFTTFDRDNDEYSYNCAVDYTGAWWYHSSCLNCNLNGKWGSSDFAKGVNWYDLSRFDSSVSFTEM<br>KIREI
**PREDICTED: Ficolin-2-like** [*Biomphalaria glabrata*] (100%); Sequence ID: XP_013071685<br><br>Domains:<br>-Ig: none<br>-Coiled coil: 12-53<br>-FBG: 71-280	LGUN_random_Scaffold11134:<br>377-790; 2673-3506	MLIVTVATATFDDLVKVIQKLMKQADEDKEKIQENKQKIKNIQEDFDTRQQNLSPFKDILVNGKKDYKEMNTSDSILPD<br>RPCNVNSTKRRLVLNLASGLKVMCDTMTDGGGWIIIQRRIKGMVDFNRDWQEYRDGFGDYNIGEFYLGNENIYKLTSS<br>GNYELRVDLEINYSFYYALYSKFQIMNESNKYKLKISGYLGNATDSLAWHDNLNFSTYKDNKNNIPNCLFKYPGPGGWW<br>FGAGHGSNLNGVWRSDRFNQGINWYGLTGYKKSAYFSEMKIREKY

**PREDICTED: Fibrinogen C domain-containing protein** [*Biomphalaria glabrata*] (50%); XP_013072469.1		
**PREDICTED: Angiopoietin-4-like** [*Biomphalaria glabrata*] (100%); Sequence ID: XP_013069966<br><br><u>Domains:</u><br>-Ig: none<br>-Coiled coil: 97-192<br>-Internal repeat: 202-286/ 286-363<br>-FBG: 403-563<br><br>**PREDICTED: Fibrinogen C domain-containing protein** [*Biomphalaria glabrata*] (47%); XP_013093995.1	LG3_random_Scaffold99: 2047-3202; 5313-5785	MYIFHETNGVIATINKGQTVVTAIQEVGSNNVKGELSDEKLHESYLQVTWRNLRSSDSGKYFCGAHVIGSDGRAERLNEMVTIQVKLPTFEDLVNVIQKLLIQANDDREILQDNKQNIKSIQNELRNNQDNVIKMYKDLDNHEQNFIRIKKDLDSNQQNIKIINQDLDNQKQNIIRINTDLDSKEQQFTRFNKDLDSKQQDIIRIDKDLNATEHNITRINKDIDSKQQDIIRIDKDLNATEHNITRINKDIDSKQQDIIRIDKDLNATKHNITRINKDIDSKQQDIIRIDKDLNATKHNITRINKDLDSKQQNIIRIDKDLNATKHNITRINKNLDSKQQDIISIKQDLESSKKDFNEHRQNISIFKDILDTVIYNLSSSLTDVKQELTKELSDGIKCISGHIHAASCRNVRSSDTYVFVLLPSGLKVMCDTKTDGGGWIIFQRRINGKVNFYRGWEEYRGGFGDFDIGEFYLGNENIFKLTSTGQYDLRIDLEFKNTKYFAQYEDFKVLSETEKYKLQIGDYSGYAGDDLSPHNNMFFSTFDRDNDGQKKISTVHKVIIKNQNENLISDSK
**PREDICTED: Fibrinogen C domain-containing protein** [*Biomphalaria glabrata*] (100%); Sequence ID: XP_013072469<br><br><u>Domains:</u><br>-Signal peptide: 1-19<br>-Ig: 27-141<br>-Coiled coil: 383-450<br>-FBG: 452-627	LGUN_random_Scaffold1199: 57142-57604;61307-61835; 65995-66891	MAHMVHLTLCVVLVSLSSSELIIDVRPDIISLEITPQLVINCSITNNKVQHLDVMNSLTLSRYDKTIRDFIALITLDSSTLNLKQLTKFSNSQVSFGNLYLALTLHYPTQFDAKVYRCSVNGNNANGTDVSLSAKKGVEYETNSTALIEEIRRLKKDEDTYKCSFKKTDRPGSRVHFFASSEIIKERIEPLTLNCSFQVSYLFISFHNVFNNMSTPEAYETVEHILYEYRMLRNTIEDTTSFETERTGAWLGLTLYGDKRTLQNTGRDLVETNGVIATINKGQPVLTTIHGGHSNSRNAKGEIFDNELKDSYLQVTLSNLQFSESGKYFCEAHVKRSQGGVERLNKMLAISVLSPTFEDLVKVIEKVLRKSDEDNIRIRENGQTLRNIQEDSDTKEKNIKSIEEDLNTKQQNIISLKEDFNAFQRTMNTFREKLEVISANVSKIERERTEDKEIASCREVKTREERVVVTLASGLKVMCDTKTDGGGWIIFQRRTYGIVDFYRSWQEYRDGFGDVSIGEFYLGNEHIYNLTSSAKYDLRIDIKYNRNSYYAQYSDFQILSEKDKYKLKIGAYSGNAGDSLSYHNNMFFSTYDKDNDLYGSNCATNYLSAWWYNSCHNSNLNGQWGSKVNSNGVIWNTLTKGESASFTEIKIREK
**Galectin-related protein precursor (GREP)** [*Biomphalaria glabrata*] (100%); SequenceID: AKS26835.1<br><br><u>Domains:</u><br>Signal peptide: 1-20<br>Ig: 28-133 /208-325<br>-Gal-binding: 473-610	LGUN_random_Scaffold_18083: 2985-3023; 2124-2167; 2738-2840; 1688-2124; 624-705; 123-183;<br>LGUN_random_Scaffold_47310: 864-1192;<br>LGUN_random_Scaffold_12074 1:407-476;<br>LGUN_random_Scaffold_5606: 17851-18203; 15817-16198	MKRLLLPLLLLTSLLQGSLTELIIQVNPGTISPGLTPKLTVNCSISNESVAELNAIQSLSMSRYNETSKTFDVLYTIDKETLSLKQIVELKGAQISFGNLFILLTIPSPSQYDCQTYSCIANGYNKFGNNDSISTKVKVESRTNITEYIKEINRLKKLEANSIMEDNVPLKNIEKLILANMNIQMCSLTTNKTSEDLIEKATLHFSVNSKTIKEFLEPMIMKCTFQVSKNNASQNLTVHYMYIRHETNGVLATISRDQQVGISQDTSLSQVQGQLSDQDFNISYLQVIWIKPSPSQSGKYVCGANIVNQEGQLEKLKASLEITIEKTSLEDLVPIVIDLLAERTKVRHILEASMKEVIEIKQQVRSAVIENVRLINQNSSTSFVLFSNIEFSGCKPNHTSQIKTLAINCLRNVHCAALMCAKDLTCTPCDRSAISGLRFPDHGSEMYLKRSLVLSQVEKRLPPVLVLSESIAVPSGLRAEDLIYFRVTLLKYDNFYIDLYEDNYNINYQFRARCDDDQCSIKGDCSESDCKSKFVTIGSKTSGFWSRSFVTRDYPFVLNEEFEMYLLIHQDSVDVYIKDKLFANIKTPRKPEDVRVIQFGGTIVLHELSL

**PREDICTED: Uncharacterized protein LOC106063810, partial** [*Biomphalaria glabrata*] (100%); Sequence ID: XP_013077728<br><br><u>Domains:</u><br>-Ig: 1-73<br>-lectin domain: none (N-term seq only)<br><br>**Galectin-related protein precursor (GREP)** [*Biomphalaria glabrata*] (96%); Sequence ID: AKS26835.1	LGUN_random_Scaffold18083: 1688-2120; 624-705; 75-186;	MSLYNETSKTFDVLYTIDKETLSLKQIVELKGVQISFGNMFILLTIPSPSQYDCQTYSCIANGYNKYGNNDSISTKVKVESRTNITEYIKEINRLKKLEANSIMEDNVPLKNIEKLILANMNIQMCSLTTNKTSEDLIEKATLHFSVNSKTIKELLQPMIMKCTFQVSKNDASQNFTVHYMYIRHGNKDTTIHWRIQEGGG
**C-type lectin-related protein 2 precursor, partial (CREP2)** [*Biomphalaria glabrata*] (98.5%); Sequence ID: AKS26832<br><br><u>Domains:</u><br>-Ig-like: 15-132<br>-Coiled coil: 142-174<br>-C-lect: 196-325	LGUN_random_Scaffold35213: 1403 -1468<br>LGUN_random_Scaffold34937: 746 -1051 (71.9%)<br>LGUN_random_Scaffold205317: 1 -272 (98.2%)<br>LGUN_random_Scaffold46202: 1045-1228 (100%)<br>LGUN_random_Scaffold113935: 1-178 (97.8%)	FRELDLGSRLTFTGNSETIKEIFEPLILSCSFQVAKHDVKENGNVILMYIFQETKGVIATISKGQPFAYSTEPRHTFAQGNIGDEKLNKSYLQVTRKNPNSLESGKYFCLAHVRDSAGKDKVFHASIKINIEKSNSDDLVPALGRVLERLDRIEERQEATEQKIVDAQEDFNIQIGNLSSVLDQEKPKKEEDNVLCNAGFHKITDGQISACVWVSNVSMNYIQARDDCKEKGAHLLTLKTQEKFTLLHKNDPGRYLWIGLTDRLIEDIFKWDDDNSICNPLCRGRLFSTGEPNDSGGEEDCVVYYPTRMKLNDNNCGSTNHYMCEHPI
**C-type lectin-related protein 3 precursor, partial (CREP3)** [*Biomphalaria glabrata*] (100%); Sequence ID: AKS26833<br><br><u>Domains:</u><br>-Signal peptide: 1-19<br>-Ig: 29-145<br>-Coiled-coil: 226-251<br>-C-lect: 261-360	LG3_random_Scaffold2051: 76877-77044; 74469-74550;<br>LGUN_random_Scaffold38320: 919-1386;<br>LG3_random_Scaffold2051: 66872-67025; 63650-63835	MKTLHLVLALCAFIVTVLCSSPTVTFTANSETVKEQFQPLILSCSVKVSGNEPIQDANVHLLYILHETKRIIARISKDQPVGTSQNPGSIQVQGQINENKAMHSYLQVTWTNPKFTDSGKYFCLAHVRNSTGQDNVLDADITINVGKLTTDDLTMVVSRLLERVDEGEVKQEENRQNILSVEEDLTDRLVNTTIVVSRLLERVNNGEEKQDENSRKLLKMEEYVYNLIANLSRIQEALNQNIEKATLKMETTTITTSRGNCKRGFDKITYGQLFACVWVSSAPLDFFGARDSCKALGARLMTLKTRDKFNLLETNNVGNKEFYIGLTDFVTEGIFRWTDDDSISNLSSIQSFFRTGEPNDHAG

**PREDICTED: Uncharacterized protein LOC106065543** [*Biomphalaria glabrata*] (100%); Sequence ID: XP_013079834<br><br><u>Domains:</u><br>-Signal peptide: none<br>-Ig: none<br>-Coiled coil: none<br>-C-lect: 144-283<br><br>**PREDICTED: C-type lectin domain family protein** [*Biomphalaria glabrata*] (36%); Sequence ID: XP_013081459.1	LGUN_random_Scaffold20861: 821-2283	MLKINSLVLSRVTSADNDTIEELIILNYNESNNTSKTSKNPKGSIGFNGASYVSLTWPNPSYRDASKYKCEARGVSTSFNKI SAAAYASVETDEPDIRSLVDELLHLRKETYETSNSLKEVKQKYASLNEVLQKSVNKLNAEKQLSKNSFFISSNVFQGKRYY MSRKQASNDINKAQATCAFYGGYLVEIDTTDEYTFVVNFIKSVSANDSTFSCVYNGLMDPENDGIWTHVFTKKDSKFLP WGKGEPQNTADYNCMCLEKSYNWRYNDLPCYYADALRYLCEIPEDEQTVSN
**PREDICTED: Uncharacterized protein LOC106062883 "CREP-Like"** [*Biomphalaria glabrata*] (100%); Sequence ID: XP_013076651<br><br><u>Domains:</u><br>-Signal peptide: 1-19<br>-Ig: 26-148<br>-Coiled coil: 148-176<br>-C- lect: 199-335<br><br>**PREDICTED: C-type lectin domain family protein** [*Biomphalaria glabrata*] (49%); Sequence ID: XP_013081459.1	LGUN_random_Scaffold16778: 502-660; 1390-2477	MITIPCVFLCVLYVAVSWAATIDIKPNVITVPVTRNLVIVCSLKRVNTPELSALFSLTLLHSNAMDEPKFTSLASVNTLDNKI STFSLDESATVAGLINNSGESFLRLTWKNPMSEKSGLYRCSFNGVGVLGNPLTLTSTGAVVVSVTDYDSLTDKMKDFEN SSKELKAKFDILSDYTNNLHTNYNSLISRLSRSRDAAFYSTSSFNGSRYYLPRDLDVLQVTWAQATCELYGGHLAEIDSNEE FHFVRNFLVSKNLFDVVVTGANDEEKEGTWIYNRNKAPLKVFNWATTEPNSGRIANCQCFWKGGNWYMADARCVY FNNKPNDGYVYSYLCEMPESSTDV
**PREDICTED: Uncharacterized protein LOC106065054** [*Biomphalaria glabrata*] (100%); Sequence ID: XP_013079269; 612 aa	LG4_random_Scaffold311: 172599 -172997; 177150-177253; 177834-177972; 179067-179206; 182629 182787; 183969 -184116;	MLFLKLVTLVLLYKCNKVLADDDTPAETQSTVNWYCPEGWTAFNDGHNITCLWFSNTSLYQVKAQTICKNFGSSLVKL HTEDKIKAFKENFSKLENDIYIGLRKERGYDASYMWDGINGLEIANISILPTSVKDSIHYKLSCVYINTQGEWDIDDCETEY NFVCEKSVECKPGSFGDKCFNQCHCLGEPCNPKSSEIRCKYGCQTGWMGNACDLEKQDPDVKYYCVNSQEKGKYVNI RIYTKGVHYRSIYGLNVNKTRSPWCYGSSISEDIDSSLPVTITIPVKEELQEVLESGSCAGEHLSNDTYLWTVVIKENEGILLE HDLQVTIKCDFSKSESLIRNSAYSVIGQPTELHKQFLEPQSTEDDVSMQVVDSYSGQVISEAAVGSSVTLTIKFGLKEGSLI

<u>Domains :</u><br>-Signal peptide: 1-20<br>-C-lectin: 36-165<br>-EGF: 170-212<br>-ZP: 262-474<br>-Trans-membr: 549-571	185191-185348; 187829-187984; 191164-191717	KGITPYDCYATSPDNKFKKQLIDSQGCPMSGSPISAFSERDDNSIQTQWFPLFAFEGEQNVVFQCSFDLCFLKGCQVGCRAFRFHKRSAEDYPETLHEHFTKSTIAVLANKIPISMPESESADNNSLSKSSQTPKSSNILAYFNPVTCSIFLVLLVLIIVTYMSFMYALRKSVGNIRREIDYVISRDKLLCYGKIPKDKNCDCNCPYNC
**PREDICTED: Uncharacterized protein LOC106061984** [*Biomphalaria glabrata*] (100%); Sequence ID: XP_013075688<br><br><u>Domains:</u><br>-low complexity: 25-37/ 58-72<br>-Ig-like : 88-206<br>-Coiled coil: 216-276<br>-lectin domains: none<br><br>**PREDICTED: Fibroleukin-like** [*Biomphalaria glabrata*] (45%); Sequence ID: XP_013070005.1	LGUN_random_Scaffold15796: BGLB005673	MHYRNINCQTANLHFISEFPARLVTTTTSSSTPETTTKILSTKIEWYTSTTAQPKIKTTSTVTMKQPMTTTTVNISSPPESRLQIYGSSEIIQELIEPLTIKCLYKTLIQGERDTSALQSLYLLHETNGVIAYINKQQPVIATIQGDNFKNVEGEIFDNESKDSYLQVTWNDLKRSESGKYFCEAYVNHSDGRFDKMNEMLTIIVKSPTFDDLVKVIQKLQRQAEVDKETVRENQHKIKTIQEDLDTKQQDIISLKEDMNTTKQDIMSIKEDLDTKSQNILSIRENFDTNKHNMIIFQDNLTMTVANLSAALMEVENSGKVFTFYIISLYSLCEAYILN
