# Supplementary material for: Proteomic analysis of Biomphalaria glabrata plasma proteins with binding affinity to those expressed by early developing larval Schistosoma mansoni
Source: PLoS Pathog. 2017 May 16;13(5):e1006081. doi: 10.1371/journal.ppat.1006081 (PMC5433772; doi:10.1371/journal.ppat.1006081)
Supplement: S4 Table — Protein identifications (Sequence ID) were determined using Mascot and Sequest against the current B. glabrata annotated NCBInr database. The numbers following the Scaffold idenfications (:xxx-yyy) designate the location of the coding gene sequence within the designated scaffold. Peptide sequences in red represent the original peptides identified in the tandem MS analyses of plasma proteins. (PDF) [file ppat.1006081.s005.pdf]

**S4 Table. Protein sequences, identified domains and genomic locations in VectorBase of NMRI and BS-90 *Biomphalaria glabrata* VIgL domain-containing plasma proteins eluted from larval transformation protein (LTP) affinity columns.**

| Protein ID | Scaffold No. | Predicted Protein Sequence |
|---|---|---|
| **FREP 2.28** [*Biomphalaria glabrata*] (100%); Sequence ID: ADK11410<br><br>Domains:<br>-Signal peptide: 1-20<br>-Ig: 38-148<br>-FBG: 181-382 | 500-1158:<br>LGUN_random_Scaffold563550: 640-1300; rev. frame; 1994bp<br>LGUN_random_Scaffold9301: 368-1028; 12407bp<br>199-502:<br>LGUN_random_Scaffold534902: 1310-1613; 1684bp | MASLPLRLVLLVSMVTLIRSSSWLNFTGNSETIRELIQPLKLTCTFQISKNDSENDSQVLFMSIYHETKRVIASISK<br>YQPVATSLYPSVTKVQGQIYHSNESKDSYLQVTWTHPKLSESGKYFCLAHAWNSTSQNSVFDADITVNVIKSST<br>DDLAVALSYIQDRLDKDGVDSIQISRASPTLPESCRDVISSEDRVVVTLASGLKVMCDTKTDGGGWIIFQRRING<br>YVDFYRGWKEYRDGFGDYDIGEFYLGNENIFKLTSSKKYDLRIDLEFNNTKYFAFYTRFEILGEQDYYKLQIGGYS<br>GNAGDALTNIHNDKFFSTYDKDNDLGTSGSCAVTHKGAWWYRDCYDSSLNGKWGSDRINWSKLTGITKSVT<br>FTEMKIREIELN |
| **FREP 3-2 precursor** [*Biomphalaria glabrata*] (100%); Sequence ID: AAK28656<br><br>Domains:<br>-Signal peptide: 1-18<br>-Ig: 28-134/183-301<br>-Coiled coil: 314-382<br>-FBG: 451-655 | LG3_random_Scaffold2841: 18324:26062; BGLB000204; 253aa/ 655aa; 65764bp | MARLFLLFILCVFVVSLAGSELVIDVQPNVISPEITPQLVINCSITNNEVQTLDLIKSLTLSRYNETIREFDELIALDSL<br>TLNLKQFVRFKYSQISFGNRYITLILHNPTQFDARIYKCNATGTNSEGANISLFAKKAVEYETNSTALIEEIRRIKKD<br>ENYCSFKKDDLSDSKQRSRVYFSGSSDIIKERIEPLTLKCTFQVLKTDQNETSRLQSLYILHESKGVIAYVNKDQPV<br>VTSLQGSNIQDVEGEIYDNAIKDSYLQVTWSNLKHSESGKYFCEAHNKYSEGRIDKSSNMLTITVERPTFDDLVE<br>AMHKLFTQVDGAKESLKAINQNIKNINKDLDFKEQNITSIKQEVIRNQNNIQILSEDLNIKEQNLTSIKADLSTKQ<br>QTFLNIKEDVILNQQIIHKIKQDLNTYRHNMSNIEEHLEVILTNLSTASIKVKNQTDEGSKMSYPPRKSCRDVNS<br>TDERVVVTLTSGLKVMCDTKTDGGGWIIFQRRINGNVDFYRGWKEYRDGFGDYNIGEFYLGNENIYMLTSTG<br>QYNLRIDLKYKNKAFFAQYSGFKILSEKEKYKLNIGAYSGNSGDNFSSHNNAFFTTFDRDNDEYSYNCAVDYTG<br>AWWYHSSCLNCNLNGKWGSSDFAKGVNWYDLSRFDSSVSFTEMKIREI |
| **FREP12 FBG variant 2, partial** [*Biomphalaria glabrata*] (100%); Sequence ID: AAT58639;<br><br>Domains:<br>-Ig: none; C-term seq only<br>-FBG: 1-128 | LGUN_random_Scaffold1434: 56555:64579: BGLB000011 (86%)<br>LGUN_random_Scaffold2702: 15650:23178:BGLB000021 (86%)<br>LGUN_random_Scaffold4104: 4421:17121:BGLB0000133 (85%) | TKTDGGGWIIFQRRINGKVNFYRGWKEYRDGFGDYDIGEFYLGNENIFKLTSTGQYDLRIDLEFKNTKYFAQYE<br>DFKVLSETEKYKLQIGDYLGNAGDDLSPHNNMFFSTFDRDNDVDSHLNCAEYCS |
| **PREDICTED: ficolin-2-like** [*Biomphalaria glabrata*] (100%); Sequence ID: XP_013071685; | LGUN_random_Scaffold11134: 377-790; 2673-3506 | MLIVTVATATFDDLVKVIQKLMKQADEDKEKIQENKQKINIQEDFDTRQQNLSPFKDILVNGKKDYKEMNTSD<br>SILPDRPCNVNSTKRRLVLNLASGLKVMCDTMTDGGGWIIIQRRIKGMVDFNRDWQEYRDGFGDYNIGEFYL<br>GNENIYKLTSSGNYELRVDLEINYSFYYALYSKFQIMNESNKYKLKISGYLGNATDSLAWHDNLNFSTYKDNKN<br>NIPNCLFKYPGPGGWWFGAGHGSNLNGVWRSDRFNQGINWYGLTGYKKSAYFSEMKIREKY |

| | | |
|---|---|---|
| Domains:<br>-Ig: none<br>-Coiled coil: 12-53<br>-FBG: 71-280<br><br>**PREDICTED: Fibrinogen C domain-containing protein** [*Biomphalaria glabrata*] (50%); Sequence ID: XP_013072469.1 | | |
| **PREDICTED: angiopoietin-4-like** [*Biomphalaria glabrata*] (100%); Sequence ID: XP_013069966<br><br>Domains:<br>-Ig: none<br>-Coiled coil: 97-192<br>-Internal repeat: 202-286/ 286-363<br>-FBG: 403-563<br><br>**PREDICTED: Fibrinogen C domain-containing protein** [*Biomphalaria glabrata*] (47%); Sequence ID: XP_013093995.1 | LG3_random_Scaffold99:2047-3202; 5313-5785 | MYIFHETNGVIATINKGQTVVTAIQEVGSNNVKGELSDEKLHESYLQVTWRNLRSSDSGKYFCGAHVIGSDGRAERLNEMVTIQVKLPTFEDLVNVIQKLLIQANDDREILQDNKQNIKSIQNELRNNQDNVIKMYKDLDNHEQNFIRIKKDLDSNQQNIKIINQDLDNQKQNIIRINTDLDSKEQQFTRFNKDLDSKQQDIIRIDKDLNATEHNITRINKDIDSKQQDIIRIDKDLNATEHNITRINKDIDSKQQDIIRIDKDLNATKHNITRINKDIDSKQQDIIRIDKDLNATKHNITRINKDLDSKQQNIIRIDKDLNATKHNITRINKNLDSKQQDIISIKQDLESSKKDFNEHRQNISIFKDILDTVIYNLSSSLTDVKQELTKELSDGIKCISGHIHAASCRNRSSDTYVFVLLPSGLKVMCDTKTDGGGWIIFQRRINGKVNFYRGWEEYRGGFGDFDIGEFYLGNENIFKLTSTGQYDLRIDLEFKNTKYFAQYEDFKVLSETEKYKLQIGDYSGYAGDDLSPHNNMFFSTFDRDNDGQKKISTVHKVIIKNQNENLISDSK |
| **PREDICTED: Fibrinogen C domain-containing protein** [*Biomphalaria glabrata*] (100%); Sequence ID: XP_013072469<br><br>Domains:<br>-Signal peptide: 1-19<br>-Ig: 27-141 | LGUN_random_Scaffold1199: 57142-57604;61307-61835; 65995-66891 | MAHMVHLTLCVVLVSLSSSELIIDVRPDIISLEITPQLVINCSITNNKVQHLDVMNSLTLSRYDKTIRDFIALITLDSSTLNLKQLTKFSNSQVSFGNLYLALTLHYPTQFDAKVYRCSVNGNNANGTDVSLSAKKGVEYETNSTALIEEIRRLKKDEDTYKCSFKKTDRPGSRVHFFASSEIIKERIEPLTLNCSFQVSYLFISFHNVFNNMSTPEAYETVEHILYEYRMLRNTIEDTTSFETERTGAWLGLTLYGDKRTLQNTGRDLVETNGVIATINKGQPVLTTIHGGHSNSRNAKGEIFDNELKDSYLQVTLSNLQFSESGKYFCEAHVKRSQGGVERLNKMLAISVLSPTFEDLVKVIEKVLRKSDEDNIRIRENGQTLRNIQEDSDTKEKNIKSIEEDLNTKQQNIISLKEDFNAFQRTMNTFREKLEVISANVSKIERERTEDKEIASCREVKTREERVVVTLASGLKVMCDTKTDGGGWIIFQRRTYGIVDFYRSWQEYRDGFGDVSIGEFYLGNEHIYNLTSSAKYDLRIDIKYNRNSYYAQYSDFQILSEKDKYKLKIGAYSGNAGDSLSYHNNMFFSTYDKDNDLYGSNCATNYLSAWWYNSCHNSNLNGQWGSKVNSNGVIWNTLTKGESASFTEIKIREK |

| | | |
|---|---|---|
| -Coiled coil: 383-450<br>-FBG: 452-627 | | |
| **Galectin-related protein precursor (GREP)** [*Biomphalaria glabrata*] (100%); SequenceID: AKS26835.1<br><br><u>Domains:</u><br>-Signal peptide: 1-20<br>-Ig: 28-133/208-325<br>-Gal-binding: 473-610 | LGUN_random_Scaffold_18083: 2985-3023; 2124-2167; 2738-2840; 1688-2124; 624-705; 123-183;<br>LGUN_random_Scaffold_47310: 864-1192;<br>LGUN_random_Scaffold_120741: 407-476;<br>LGUN_random_Scaffold_5606: 17851-18203; 15817-16198 | MKRLLLPLLLLTSLLQGSLTELIIQVNPGTISPGLTPKLTVNCSISNESVAELNAIQSLSMSRYNETSKTFDVLYTIDKETLSLKQIVELKGAQISFGNLFILLTIPSPSQYDCQTYSCIANGYNKFGNNDSISTKVKVESRTNITEYIKEINRLKKLEANSIMEDNVPLKNIEKLILANMNIQMCSLTTNKTSEDLIEKATLHFSVNSKTIKEFLEPMIMKCTFQVSKNNASQNLTVHYMYIRHETNGVLATISRDQQVGISQDTSLSQVQGQLSDQDFNISYLQVIWIKPSPSQSGKYVCGANIVNQEGQLEKLKASLEITIEKTSLEDLVPIVIDLLAERTKVRHILEASMKEVIEIKQQVRSAVIENVRLINQNSSTSFVLFSNIEFSGCKPNHTSQIKTLAINCLRNVHCAALMCAKDLTCTPCDRSAISGLRFPDHGSEMYLKRSLVLSQVEKRLPPVLVLSESIAVPSGLRAEDLIYFRVTLLKYDNFYIDLYEDNYNINYQFRARCDDDQCSIKGDCSESDCKSKFVTIGSKTSGFWSRSFVTRDYPFVLNEEFEMYLLIHQDSVDVYIKDKLFANIKTPRKPEDVRVIQFGGTIVLHELSL |
| **PREDICTED: Uncharacterized protein LOC106063810, partial** [*Biomphalaria glabrata*] (100%); Sequence ID: XP_013077728<br><br><u>Domains:</u><br>-Ig: 1-73<br>-lectin domain: none (N-term seq only)<br><br>**Galectin-related protein precursor (GREP)** [*Biomphalaria glabrata*] (96%); Sequence ID: AKS26835.1 | LGUN_random_Scaffold18083: 1688-2120; 624-705; 75-186; | MSLYNETSKTFDVLYTIDKETLSLKQIVELKGVQISFGNMFILLTIPSPSQYDCQTYSCIANGYNKYGNNDSISTKVKVESRTNITEYIKEINRLKKLEANSIMEDNVPLKNIEKLILANMNIQMCSLTTNKTSEDLIEKATLHFSVNSKTIKELLQPMIMKCTFQVSKNDASQNFTVHYMYIRHGNKDTTIHWRIQEGGG |
| **C-type lectin-related protein 2 precursor, partial (CREP2)** [*Biomphalaria glabrata*] (100%); Sequence ID: AKS26832<br><br><u>Domains:</u><br>-Ig-like: 15-132 | LGUN_random_Scaffold35213: 1403 -1468 (98.5%)<br>LGUN_random_Scaffold34937: 746-1051 (71.9%)<br>LGUN_random_Scaffold205317: 1 -272 (98.2%)<br>LGUN_random_Scaffold46202: 1045-1228 (100%) | FRELDLGSRLTFTGNSETIKEIFEPLILSCSFQVAKHDVKENGNVILMYIFQETKGVIATISKGQPFAYSTEPRHTFAQGNIGDEKLNKSYLQVTRKNPNSLESGKYFCLAHVRDSAGKDKVFHASIKINIEKSNSDDLVPALGRVLERLDRIEERQEATEQKIVDAQEDFNIQIGNLSSVLDQEKPKKEEDNVLCNAGFHKITDGQISACVWVSNVSMNYIQARDDCKEKGAHLLTLKTQEKFTLLHKNDPGRYLWIGLTDRLIEDIFKWDDDNSICNPLCRGRLFSTGEPNDSGGEEDCVVYYPTRMKLNDNNCGSTNHYMCEHPI |

| | | |
|---|---|---|
| -Coiled coil: 142-174<br>-C-lect: 196-325 | LGUN_random_Scaffold113935: 1-178 (97.8%) | |
| **C-type lectin-related protein 3 precursor, partial (CREP3)** [*Biomphalaria glabrata*] (100%); Sequence ID: AKS26833<br><br><u>Domains:</u><br>-Signal peptide: 1-19<br>-Ig: 29-145<br>-Coiled-coil: 226-251<br>-C-lect: 261-360 | LG3_random_Scaffold2051: 76877-77044; 74469-74550; LGUN_random_Scaffold38320: 919-1386; LG3_random_Scaffold2051: 66872-67025; 63650-63835 | MKTLHLVLALCAFIVTVLCSSPTVTFTANSETVKEQFQPLILSCSVKVSGNEPIQDANVHLLYILHETKRIIARISKDQPVGTSQNPGSIQVQGQINENKAMHSYLQVTWTNPKFTDSGKYFCLAHVRNSTGQDNVLDADITINVGKLTTDDLTMVVSRLLERVDEGEVKQEENRQNILSVEEDLTDRLVNTTIVVSRLLERVNNGEEKQDENSRKLLKMEEYVYNLIANLSRIQEALNQNIEKATLKMETTTITTSRGNCKRGFDKITYGQLFACVWVSSAPLDFFGARDSCKALGARLMTLKTRDKFNLLETNNVGNKEFYIGLTDFVTEGIFRWTDDDSISNLSSIQSFFRTGEPNDHAG |
| **PREDICTED: Uncharacterized protein LOC106065543** [*Biomphalaria glabrata*] (100%); Sequence ID: XP_013079834<br><br><u>Domains:</u><br>-Signal peptide: none<br>-Ig: none<br>-Coiled coil: none<br>-C-lect: 144-283<br><br>**PREDICTED: C-type lectin domain family protein** [*Biomphalaria glabrata*]; (36%); Sequence ID: XP_013081459.1 | LGUN_random_Scaffold20861: 821-2283 | MLKINSLVLSRVTSADNDTIEELIILNYNESNNTSKTSKNPKGSIGFNGASYVSLTWPNPSYRDASKYKCEARGVSTSFNKISAAAYASVETDEPDIRSLVDELLHLRKETYETSNSLKEVKQKYASLNEVLQKSVNKLNAEKQLSKNSFFISSNVFQGKRYYMSRKQASNDINKAQATCAFYGGYLVEIDTTDEYTFVVNFIKSVSANDSTFSCVYNGLMDPENDGIWTHVFTKKDSKFLPWGKGEPQNTADYNCMCLEKSYNWRYNDLPCYYADALRY LCEIPEDEQTVSN |
| **PREDICTED: Uncharacterized protein LOC106062883 "CREP-Like"** [*Biomphalaria glabrata*] (100%); Sequence ID: XP_013076651 | LGUN_random_Scaffold16778: 502-660; 1390-2477 | MITIPCVFLCVLYVAVSWAATIDIKPNVITVPVTRNLVIVCSLKRVNTPELSALFSLTLLHSNAMDEPKFTSLASVNTLDNKISTFSLDESATVAGLINNSGESFLRLTWKNPMSEKSGLYRCSFNGVGVLGNPLTLTSTGAVVVSVTDYDSLTDKMKDFENSSKELKAKFDILSDYTNNLHTNYNSLISRLSRSRDAAFYSTSSFNGSRYYLPRDLDVLQVTWAQATCELYGGHLAEIDSNEEFHFVRNFLVSKNLFDVVVTGANDEEKEGTWIYNRNKAPLKVFNWATTEPNSGRIANCQCFWKGGNWYMADARCVYFNNKPNDGYVYSYLCEMPESSTDV |

| | | |
|---|---|---|
| Domains:<br>-Signal peptide: 1-19<br>-Ig: 26-148<br>-Coiled coil: 148-176<br>-C-lect: 199-335<br><br>**PREDICTED: C-type lectin domain family protein** [*Biomphalaria glabrata*] (32%); Sequence ID: XP_013081459.1 | | |
| **PREDICTED: Uncharacterized protein LOC106062128** [*Biomphalaria glabrata*] (100%); Sequence ID: XP_013075857<br><br>Domains:<br>-Ig: none<br>-Internal repeat: 1-64/ 57/120<br>-C-lect: 1-61<br><br>**PREDICTED: Techylectin-like protein** [*Biomphalaria glabrata*] (89%); Sequence ID:XP_013089083.1 | LGUN_random_Scaffold6870 :6587:15188: BGLB000131 (91%; 147/156) | MNSTKQDIISLKDNMNSTKQYIVSIKKDLDTTQANMKSLNEDFYTNIQNITSIKKDMNTYKENILRIETDLDSNKQNIVSIRKEADTNKQDIIRIKEDLDTKQQNMLSIRENVDTNRHNMSIFQENLTMMVANLSTELKEVQSQIHKDPRKMGELE |
| **PREDICTED: Uncharacterized protein LOC106065054** [*Biomphalaria glabrata*] (100%); Sequence ID: XP_013079269<br><br>Domains :<br>-Signal peptide: 1-20<br>-C-lectin: 36-165<br>-EGF: 170-212 | LG4_random_Scaffold311: 172599 -172997; 177150-177253; 177834-177972; 179067-179206; 182629 182787; 183969 -184116; 185191-185348; 187829-187984; 191164-191717 | MLFLKLVTLVLLYKCNKVLADDDTPAETQSTVNWYCPEGWTAFNDGHNITCLWFSNTSLYQVKAQTICKNFGSSLVKLHTEDKIKAFKENFSKLENDIYIGLRKERGYDASYMWDGINGLEIANISILPTSVKDSIHYKLSCVYINTQGEWDIDDCETEYNFVCEKSVECKPGSFGDKCFNQCHCLGEPCNPKSSEIRCKYGCQTGWMGNACDLEKQDPDVKYYCVNSQEKGKYVNIRIYTKGVHYRSIYGLNVNKTRSPWCYGSSISEDIDSSLPVTITIPVKEELQEVLESGSCAGEHLSNDTYLWTVVIKENEGILLEHDLQVTIKCDFSKSESLIRNSAYSVIGQPTELHKQFLEPQSTEDDVSMQVVDSYSGQVISEAAVGSSVTLTIKFGLKEGSLIKGITPYDCYATSPDNKFKKQLIDSQGCPMSGSPISAFSERDDNSIQTQWFPLFAFEGEQNVVFQCSFDLCFLKGCQVGCRAFRFHKRSAEDYPETLHEHFTKSTIAVLANKIPISMPESESADNNSLSKSSQTPKSSNILAYFNPVTCSIFLVLLVLIIVTYMSFMYALRKSVGNIRREIDYVISRDKLLCYGKIPKDKNCDCNCPYNC |

| | | |
|---|---|---|
| -ZP: 262-474<br>-Trans-membr: 549-571 | | |
| **PREDICTED: Uncharacterized protein LOC106061984** [*Biomphalaria glabrata*] (100%); Sequence ID: XP_013075688<br><br><u>Domains:</u><br>-low complex: 25-37/ 58-72<br>-Ig-like : 88-206<br>-Coiled coil: 216-276<br>-Lectin domains: none<br><br>**PREDICTED: Fibroleukin-like** [*Biomphalaria glabrata*] (45%); Sequence ID: XP_013070005.1 | LGUN_random_Scaffold15796: BGLB005673 (100%) | MHYRNINCQTANLHFISEFPARLVTTTTSSSTPETTTKILSTKIEWYTSTTAQPKIKTTSTVTMKQPMTTTTVNIS<br>SPPESRLQIYGSSEIIQELIEPLTIKCLYKTLIQGERDTSALQSLYLLHETNGVIAYINKQQPVIATIQGDNFKNVEGE<br>IFDNESKDSYLQVTWNDLKRSESGKYFCEAYVNHSDGRFDKMNEMLTIIVKSPTFDDLVKVIQKLQRQAEVDK<br>ETVRENQHKIKTIQEDLDTKQQDIISLKEDMNTTKQDIMSIKEDLDTKSQNILSIRENFDTNKHNMIIFQDNLT<br>MTVANLSAALMEVENSGKVFTFYIISLYSLCEAYILN |
