# Supplementary material for: Proteomic analysis of Biomphalaria glabrata plasma proteins with binding affinity to those expressed by early developing larval Schistosoma mansoni
Source: PLoS Pathog. 2017 May 16;13(5):e1006081. doi: 10.1371/journal.ppat.1006081 (PMC5433772; doi:10.1371/journal.ppat.1006081)
Supplement: S5 Table — Protein identifications (Sequence ID) were determined using Mascot and Sequest against the current B. glabrata annotated NCBInr database. The numbers following the Scaffold idenfications (:xxx-yyy) designate the location of the coding gene sequence within the designated scaffold. Peptide sequences in red represent the original peptides identified in the tandem MS analyses of plasma proteins. (PDF) [file ppat.1006081.s006.pdf]

S5 Table. Protein sequences, identified domains and genomic locations in VectorBase of other immune-related NMRI and BS-90 *Biomphalaria glabrata* plasma proteins eluted from sporocyst membrane-enriched fraction (Mem) affinity columns.

Protein ID	Scaffold No.	Predicted Protein Sequence
**PREDICTED: Lipopolysaccharide-binding protein-like** [*Biomphalaria glabrata*] (100%);<br>Sequence ID: XP_013070669.1<br><br><u>Domains:</u><br>-Bactericidal permeability-increasing protein (BPI) / Lipopolysaccharide-binding protein (LBP): 1-193/ 180-384	LGUN_random_Scaffold1023: 50441-50613; 50252-50331; 49394-49526;46627-46718; 45543-45623; 43433-43591; 40841-40951; 39401-39593	MLLINLLGHYFNSFRLENVAIPDIDMSFNPVQRGLTMNLRNLGVDVLLDYRVRYKLLFVPISNSGGVSVN<br>FRGVELTATVGIDQFEDTSPRLVSRSCSAAIGDFNINFSGSMAWLLNLLKGLFRGKIRDTIRSKICDKVT<br>EAINTQGADKLRQIPLESDIADKFVIDYRLLSPVLFTDSYLETQHKKLYKRGTLGYAAHTHGYLQYVISP<br>DKLPAQSKGFLNTTCPDSACAGTLIPLLAEKYPNSFVTLTISSSKAPDFNITSNGLGLTLYGTLDLSTTS<br>ASAVVTANVVISIAGAAFVRNDRIVGNVAQFRTVLADIDSQRLISLYTFQISSINKALDDGMNMVVIPQL<br>NAVGDTGIALPVLKDLTFQNPRIVYQDKSVLISTNVQYQGIV
**Thioester-containing protein 1.5 (TEP1.5)** [*Biomphalaria glabrata*] (100%); Sequence ID: ADE45333<br><br><u>Domains:</u><br>-Signal peptide: 1-21<br>-Proteinase-binding alpha-macroglobulins: 126-219/ 463-599/ 718-808/ 978-1241<br>-Thiol ester: 929-958<br>-Alpha-2-macroglobulin-receptor: 1342-1426	LGUN_random_Scaffold4524: 9507-9566; 9149-9332; 8894-8916; 4705-4867; 1865-1933; 1610-1736;<br>LGUN_random_Scaffold10725: 6705-6750; 4397-4481; 2567-2681; 2042-2159; 1413-1527;<br>LGUN_random_Scaffold9700: 5915-6144; 3125-3226; 2777-2839; 709-909;<br>LGUN_random_Scaffold8482: 12144-12290; 10814-10917; 10616-10675; 10307-10337;<br>LGUN_random_Scaffold2300: 37595-37626;<br>LGUN_random_Scaffold76986: 250-267;<br>LGUN_random_Scaffold8482: 10166-10290;<br>LGUN_random_Scaffold7766: 10058-10180; 9732-9849;<br>LGUN_random_Scaffold45951: 828-903;<br>LGUN_random_Scaffold7766: 277-407;<br>LGUN_random_Scaffold53075: 386-529;<br>LGUN_random_Scaffold6063:	MRMKLNLILFVFYLVFQECQGGKYFISAPRNVVPGTAYDISVDILKQDIGNVTVEAILQDYSFSIPEGPK<br>SLLTANGTFSPGVRGTLSMPIDFNLHCSYCRILLKGYNPLQFEQDIFIQISSDILSILIQTDKAIYKPKE<br>RVNFRILAVYYNLQLYTGTFHYEILDPYDNKINVLSGVSGTFGVVEGFFDLSDQPSFGTWKINVRTETVS<br>GAESQFFEVAEYDLPRFQVDVGLPPFALLSDTTLSGSVEAKYTFGQPVYGLVLLQIGENVDTIDKCNVNR<br>KVTEISFEIKGKGNFSVPLEDIRRSVHLNEKKKIKITAFVIEASTGIKLNGSSVITYYGNRYQIKFLEMT<br>PAVFKPGLQYTAYVQVTTPDGLPPTESNLSLSVYTSVTYQMTVPDQELYSPSSFSGSYPLPGQNMSLPAN<br>GILSIDIDIPLNATSIDIKVSLNKQTTAEKRISKSYSMSNNYLQLSLLSKLVKAESDVLIKITSTEAIDS<br>LAYEIRSRSDHVKSGVLELNGQREFNATFKVGPSWAPIAQLLMYYIRRDSNEIVTDSLAFNVEGMFENKV<br>NVAFKENETDINKNVSLELSADSDSQIYVLAVDQSVLLLKTGNDLTPNKVKDSFISKFHKGEVPTDSNFA<br>LSYSGSSINEVFSNMGLVIATDLNIFAPFRPIALGRFPSSGFDRQGMMGAPMAMSFRDDNAMESASFEMD<br>VATSTKPVERVRSFFPESWLWTSVKSINGHATLTTTVPDTITSWIVSAFATNSDTGLGVAPTTSKLRVFR<br>PFFVSLTYPRSVTRNEQFIVQATVFNYLPVDLMVTVSLKENPFLTPITPGPGNQASNIQVRANEQGIVYF<br>SLSALTVGSLDTEVSARSNMAADAIVRQILIKHEGAPVVYNNPILINLSNNQSTFEKNIAFTLPDSLVPE<br>SQRIRVKVTGDLIGSTVQSLTSLLTLPTGCGEQSLVKFTPNIHIGRYLKATNQLSEELNKKIIDLLNDGY<br>QRQLTYKRYDNGFSAFGNYDISSSTWLTALVVTSFAEAQEFIFVDKEIILKASMLLIDRQNIDGSFNEFG<br>KVLDRNTQGTTTAGPALTAFVLVALLKAKELADVQDCKNNNKCRYYLLGNATLNATRNLERLMLADSIDD<br>QFSLAVASYALAEAKSQLAQSTFEKLLTFVKQEGGLEYWSANSTVNNEELNRFINWRPPRLQARPIDILI<br>TSYAILTYSSLGRLDEALPSVRWLTLQKNAQGGFVSTQDTVVGLQALSFYGSKSFRPDTNITIYVSDMNT<br>HLTMNVNSENALSLQIQEIQSNSQDFSITASGSGLALLDIEYSFNVLKELSKPVFDANTVLLDDKLDSFN<br>IMVCTKFLMKHDTGMVVQELSIPSGFVPDLSTLGQVAGVKRSERKGSIVAIYFDKISGSSLCYSIVMTRE<br>AKVAKSQKSYVRTYDYYEPANQATVFYQPRTLRDSTVCDVCLNCCP

	31434-31613; 29915-29997; 25214-25375; 23259-23332; LGUN_random_Scaffold7260: 85338740; 5514-5738; 11544-11696; 11926-11989; 12187-12304; 13837-13988; 15855-15963; 16394-16478	
**Thioester-containing protein (TEP)** [*Biomphalaria glabrata*] (100%); Sequence ID: ACL00841.1<br><br>Domains:<br>-Signal peptide: 1-21<br>-Proteinase-binding alpha-macroglobulins: 126-219/ 463-599/ 718-808/ 978-1241<br>-Thiol ester: 929-958<br>-Alpha-2-macroglobulin-receptor: 1342-1426	LGUN_random_Scaffold4524: 9507 9566; 9149-9332; 8894-8916; 4705-4867; 1865-1933; 1610-1736; LGUN_random_Scaffold10725: 6705-6750; 4397-4481; 2567-2681; 2042-2159; 1413-1527; LGUN_random_Scaffold9700: 5915-6144; 3125-3226; 2777-2839; 709-909; LGUN_random_Scaffold8482: 12144-12290; 10814-10917; 10616-10675; 10307-10337; LGUN_random_Scaffold2300: 37595-37626; LGUN_random_Scaffold76986: 250-267; LGUN_random_Scaffold8482: 10166-10290; LGUN_random_Scaffold7766: 10058-10180; 9732-9849; LGUN_random_Scaffold45951: 828-903; LGUN_random_Scaffold7766: 277-407; LGUN_random_Scaffold53075: 386-529; LGUN_random_Scaffold6063: 31434-31613; 29915-29997; 25214-25375; 23259-23332; LGUN_random_Scaffold7260: 85338740; 5514-5738; 11544-11696; 11926-11989; 12187-12304; 13837-13988; 15855-15963; 16394-16478	MRMKLNLILFVFYLVFQECQGGKYFISAPRNVVPGTAYDISVDILKQDIGNVTVEAILQDYSFSIPEGPK<br>SLLTANGTFSPGVRGTLSMPIDFNLHCSYCRILLKGYNPLQFEQDIFIQISSDILSILIQTDKAIYKPKE<br>RVNFRILAAYYNLQLYTGTFHYEILDPYDNKINVLSGVSGTFGVVEGFFDLSDQPSFGTWKINVRTETVS<br>GEESQLFEVAEYDLPRFQVDVGLPPFALLSDTTLSGSVEAKYTFGQPVYGLVLLQIGENVDTIDKCNVNR<br>KVTEISFEIKGKENFSVPLEDIRRSVHLNEKKKIKITAFVTEASTGIKLNGSSVITYYGNRYQIKFLEMT<br>PAVFKPGLQYTAYVQVTTPDGLPPTDSNLSLSVYTSVTYQMTVPDQELYSPSSFSGSYPLPGQNMSLPAN<br>GILSIDIDIPLNATSIDIKVSLNKQTTAEKRISKSYSMSNNYLQLSLLSKLVKAESDVLIKITSTEAIDS<br>LAYEIRSRSDRVKSGVLELNGQREFNATFKVEPSWAPIAQLLMYYIRRDSNEVVTDSLAFNVEGMFKNKV<br>NVAFKENETDINKNVSLELPADSDSQIYVLAVDXSVLLLKTGNDXTPXKVKDSFXSXFHKGAIPTDSNFA<br>LSYSGSSIXEVFSNMGLVIATDLNIFAPFRPIALGRFPSSGFDRQXMMGAPXAMSFXDDXAMXSASFEMD<br>VTTSTKPVERVRSFFPESWLWTSVKSINGHATLTTTVPDTITSWIGSAFATNSDTGLGVAPTTSKLPGFR<br>PFFGSLTYPRSVTRNEQFIVQATVFNYLPVDLMVTVSLKENPFLTPVTPGPGNQASNIQVRANEQRTVYF<br>SLSALIVGALDIEVSARSNMAADAIVRQILVRHEGAPVVYNNPILISLSNNQSTFEKNIAFTLPDSLVPE<br>SHRIRVKVTGDLIGSTVQSLTSLLTLPTGCGEQSLVKFTPNIHIGRYLKATNQLSKELNKKIIDLLNNGY<br>QRQLTYKRYDNGFSAFGNYDLSSSTWLTALVVTSFAEAQEFIFVDKEIILKASMLLIDRQNLDGSFNEFG<br>KVLDRNTQGTTAGPALTAFVLVALLKAKELADVQYCKNNNKCRYYLLGNATLNATRNLERLMLADSIDDQ<br>FSLAVTSYALAEAKSQLAQSTFEKLLTFVKQEGGLEYRSANSTVNNEELNRFINWRPPRLQARPIDILIT<br>SYAILTYSPLGRLDEALPSVRWLTLQKNAQGGFVSTQTDTVVGLQALSTYGSKSFRPDTNITIYVSDMNTH<br>LTMNVKSDNALSLQIQEIQSNSQDFSITASGSGLALLDIEYSFNVLKELSKPVFDVNTVLLDDKLDSFNI<br>MVCTKFLLKHDTGMVVQEVSIPSGFVPDLSTLGQVAGVKRSERKGSIVAIYFDKISGSSLCYSIVMTREA<br>KVAKSQKSYVRTYDYYEPANQATVFYQPRTLRDSTVCDVCLNCCP

**PREDICTED: CD109 antigen-like** [*Biomphalaria glabrata*] (100%);<br>Sequence ID: XP_013071291.1<br><br><u>Domains:</u><br>-Proteinase-binding alpha-macroglobulins: 47-138/ 348-500/ 649-740/ 918-1168<br>-Thiol ester: 869-898<br>-Alpha-2-macroglobulin-receptor: 1271-1355	LGUN_random_Scaffold108: 84237-84416; 83387-83461; 82641-82779; 80795-80861; 78882-79106; 78232-78463; 76389-76589; 76048-76202; 75142-75294; 51880-52111; 41664-41787; 35799-36007; 39854-39999; 39487-39666; 39487-39666; 39487-39666; 38763-38921; 36931-37116; 35799-36007; 34930-35082; 32766-32916; 31828-32071	MESLHFLFYSQLPADLPSSTYTLNVKGSGGLTFDKSENLNYNNKETSVFIQLNKAIFKPGDTVNFRVFGV<br>YSDLKSYTDPIDISIYDANSNKIKQWLKVTPTNGVITQELTLSTQPVLGDWKISVDAGRTKEEKVFTVAE<br>YVLPKFEVDVVTPSYALTTDNDVTVTVKSKYTYGKPVNGTADVLVKLHESFNTFDYSRALPVTTLQVPLN<br>GEAKVIIPMSQVKAISANLNQHVLIVIANVTESLTGNQMSGNGTVTLYDKGVKLDFPASNPKTFKPALQY<br>IAYLKVTQPDGLPMTSTAEQVKVSIRVTAELPGTTPTPYYWYVPPTESRDLPALSLAIPDNGLVAIPVDV<br>PADAKDVHVTANFQGVSKELTLGKSHSPSNSYIQLILKSGSVIKFQILSRGGIVKTGTVDANGQLVYQFS<br>IPSDSSMAPNARIVLYYVRADGEIVTDSISFDISGAFKNKVSIDLDKTDVEPGDDVTVTVKADPDSTAYC<br>LAIDQSVLLLKGGNDVTDNDVYTELKEYDTITESGSNKGIIDCPMCKRRKRMIWWPFPTYYGGSDAQQIF<br>SNAGVVVLTDATVYHYQEPIHLFNIPNFFQCGRSLNGQLRKRCFASFSPVMNLVSSVVSDPITLPVTETE<br>TKTEDLQQPTKTRSNFVETWLWNSLDIGANGSASITATVPDTITSWVASAFAINSASGLGVAPTQAHLRV<br>FRPFFVSLNLPYSVTRGEHLALQANVFNYMTEDMQVRVTLAKSDNFFNIEIDANGAEVLKQVESVQDVMI<br>KAGEAKSVYFPIVPADLGKIDIEVKAQSTKAADAVRRQLLVEAEGVPKIYNVPVLIDLTEGKTSFSKTVD<br>LTLPSNTVKGSELARISAVGDLMGPTIAGLDSLLQMPTGCGEQTMIGLAPDVYVTDYLKSVNQLSGDIQT<br>KALSYMESGYQRELTYKHTDGSFSAFGNSDASGSMWLTAFVTRVFKQAKAHIYIDDEVLIKAIQWMVSKQ<br>NANGSFPEPGNVIHKNMQGQAGSGVGLTLFVLISLLENKDLLVNTNAAGILVDEARQKALVYAEQEVAKT<br>DDLYILNMAAYAFQLANSSQVQTVLSKLEQKATVKDGRKYWHQPEQPKTTNTWDYPNPTKAVDIEMTSYA<br>LLTYAARGNIVAGKSIMQWLTEQRNSNGGFSSTQDTVLALNALSEFAKQTYSNNFNVQITTQLNATTSYT<br>FNIDKTNSLLLQSRETPNVPSQVKIDATGSGMALVQVAVSFNVESEIFETTFDLTVKLIEETINNLFVET<br>CAKWLGSGPSSAMAVQEIGIPSGFEADIESIPQLDILKRIETQNKKVILYFDQIGTTPVCLNFRAVRTGL<br>VAKSQPAAIRVYDYYEPRNQVTAFYQSQILKDSTVCDVCKECDNCV
**PREDICTED: Heat shock protein 70 kDa cognate 4** [*Biomphalaria glabrata]* (100%);<br>Sequence ID: XP_013082115.1<br><br><u>Domains:</u><br>-Heat shock proteins, Hsp70 chaperones: 6-612	LGUN_random_Scaffold253: 223515:226430: BGLB007783	MSKAPAVGIDLGTTYSCVGVFQHGKVEIIANDQGNRTTPSYVAFTDNERLIGDAAKNQVAMNPENTVFDA<br>KRLIGRRFDDPTVASDMKHWPFTVINEGGKPKIRVEYKGEKKTFFPEEISSMVLTKMKETAEAYLGKTVT<br>DAVVTVPAYFNDSQRQATKDAGTISGLNVLRIINEPTAAAIAYGLDKKVGGERNVLIFDLGGGTFDVSIL<br>TIEDGIFEVKSTAGDTHLGGEDFDNRMVNHFIQEFKRKHKKDISENKRAVRRLRTACERAKRTLSSSTQA<br>NIEIDSLFEGIDFYTSITRARFEELNADLFRGTLEPVEKSLRDAKLDKAQVHEIVLVGGSTRIPKIQKLL<br>QDFFNGKELNKSINPDEAVAYGAAVQAAILHGDKSEEVQDLLLLDVAPLSLGIETAGGVMTALIKRNTTI<br>PTKQTQTFTTYSDNQPGVLIQVYEGERAMTKDNNLLGKFELTGIPPAPRGVPQIEVTFDIDANGILNVSA<br>ADKSTGKENKITITNDKGRLSKEEIERMVNDAEKYKNEDEKQKTRISAKNALESYAFHMKSTVEDEKLKD<br>KISADDKKIIIDKCNEIIHWLDANQLADEEEFQHKQKEIEGVCNPIITKLYQGMGGAGGMPDFSGAAGAG<br>AQHSAGTGGGSGPTIEEVD
**Heat shock protein 60** [*Biomphalaria glabrata*] (100%); Sequence ID: ACL00842<br><br><u>Domains:</u><br>-Cpn60_TCP1 : 45-548	LGUN_random_Scaffold791: 163741:171751: BGLB013300	MLRVASVFRSSATRQLVPMLCRHYAKDIKFGSDARALMLQGVDLLADAVAVTMGPKGRNVILEQSWGSPK<br>ITKDGVTVAKGIDLKDKFQNIGAKLVQDVANNTNEEAGDGTTSATVLARSIAKEGFERISRGANPVEIRR<br>GVMLAVDAVVEHLKKMSRQVTTPEEIAQVATISANGDKSIGELISSAMKKVGRDGVITVKDGKTLKDELE<br>TIEGMKFDRGYISPYFMNTAKGAKCEFQDALVLLSEKKISSIQSIIPALELANQARKPLLIVAEDVDGEA<br>LSTLVLNRIKVGLQVCAVKAPGFGDNRKNTLIDMAIATGGVVFGDEGNLYKLEDIQMQDFGNVGEVTVTK<br>DDTLLMKGKGNKADIEKRIAQIKDEIEISTSEYEKEKFGERLAKLSNGVAVLKIGGTSEVEVNEKKDRIN<br>DALNATRAAVEEGIVPGGGTALLRCISVLDSVKTENEDQITGVNIIRKALRVPALTIAQNAGVDAHVVVE<br>KVLNSSGDIGYDALNNEYVNLIEQGIIDPTKVVRTALVDAAGVASLLTTAEAVVVDLPKEEKEAGMGGMG<br>MGGMGGMGGMM
**Biomphalysin** [*Biomphalaria glabrata*] (100%);<br>Sequence ID: AGG38744.1	LGUN_random_Scaffold10: 1097564:1099282: BGLB000137	MFLQIFVAVTLVQYVSSQCTYSSWWYSFDTPGQSKCNEINSYINALDRNDVNWADDALSNLEGVQCCRPP<br>APWNNVEQQVVYEDWTATLDSDYTWAFCRVGYFLQGLYRSDTGWPRFKGYLFNLESARCTKPANHPLNYG<br>TCQDIDVSSCMGRKGQCSCPGGYFLTGLYRADGDDLYFLKKIRCCTPAAKPLEMDEKSKIQTRIMDTTLW<br>NMATLAHYMGYGWCYGCHGLAVGEDFTRNGFTWAADTRTFWGKWCEGDKNGERLNLVFGDWGFAVKEIIY

Domains:<br>-Signal peptide: 1-17<br>-Aerolysin: 195-531		GKSVIEDLQAESVDSGVLYNRASSPVTESIERSKTIQETITHSTTSTFTNSHGLGVELEFEIASVKGKAS<br>YKTRFEYSTSTTNSKSISETQGFTKQSSITLGPMEGAKYEVIMSKSRTTVPYTAIITTKFSTEMKGFLRW<br>EDGNGNFHQDYRTNSGRPTFNYRFGDSSVPFYKALKKQSDNNEGVWMWGMLFQKFPDARRVTNRLTDETQ<br>YQFTLAGKLEKVEGTSVNVKWEKMKLNRRDVSGNDEPGSNITTYIAASGPADKPAVVEYPKVNLNNKEPF<br>KPIEIPVTEVKV
**Dermatopontin 2** *[Biomphalaria glabrata]* (100%); Sequence ID: AAZ80786<br><br>Domains:<br>-Dermatopontin: 11-156	LGUN_random_Scaffold8114: 1204:2972: BGLB013420	MASVASAAYVNDWDQPFNFRCPDGQVVSYVSSIHNNRREDRRWEFLCRSTRQTHSCTDSGYVNDFDGPLV<br>YTCPGNKVMVGVHSYHNNRREDRRFGFYCCDVQGSTPRDCYTTNYVNDWDEKLTLVVPEGTAVKAAYSHH<br>DNRREDRRWQFQICTL
**PREDICTED: Millepora cytotoxin-1-like** [*Biomphalaria glabrata*] (100%); Sequence ID: XP_013061974.1<br><br>Domains:<br>-Signal peptide: 1-24<br>-Dermatopontin: 29-174	LGUN_random_Scaffold7789: 3993-4096, 5800-6107, 8666-9418	MPRMYERYISIVLCSLLVLNTVHCVTYQTEEQEDWTVECAPNHVITDIVSDFFFSDRLWNITCAPIRPKI<br>NITECVWSNYHNAPQYDFLFDCPMNSILAGISSFHRSRDRVFKFYCCTNKDYLVHACQHTLTINNAKGFI<br>SFRVPEAMYVRGLFSSYDSTAGDRLYRIHLCKLDPADPTVECPTTELTTQAVTKLVNQTETPLLESESED
**PREDICTED: uncharacterized protein LOC106068782** [*Biomphalaria glabrata*] (100%);<br>Sequence ID: XP_013083706.1<br><br>Domains:<br>-Signal peptide : 1-20<br>-Zn-dependent metalloprotease, ADAM-like or reprolysin-like subgroup: 31-228<br><br>**PREDICTED: ADAM family mig-17-like isoform X2** *[Biomphalaria glabrata]* (42%); XP_013069827.1	LGUN_random_Scaffold286: 44298-44384; 42897-42974; 39464-39607; 36011-36117; 34918-35050; 34015-34151; 32322-32458; 31272-31438; 29557-29654; 27862-28646	MVRRIAVLLCLSILVMTSSCQVQNLVAEVYVVVEDSTVQSYAQQSSPAQTIAEATNSLRNDIDLVITQTN<br>VLLSSLVQYGINIEVRKRKVDILSTNIINTFLLETTYIADSSAAKASFQSWLNLQNAYSTLNYDFAVLWT<br>GFDLFGTSGIFENSITSFAAMCKSTGVSVMEFDPTARDIVATAKIIAYLLSSLNDGITSTYIMAIINAPD<br>DANRWFYSNSSAQSIKEFVALPRASCLLSTNSASVKPAVTYATYTGSLLDPDVICQRSLNDSRSYMCKSL<br>PQIYNNQPPKGNAVCSQIYCRIPKTSICTPAFTSDGMICENQKRCTKGQCSSYPTPSVDPNCLWGDQKTL<br>DYPLIPFSGTCKPFLDTYGAANCYTRPINETCCNTCKTYNSGIPGCEYGDKSSACVLFTQAQVCPIYKNT<br>LCCNYCRNYVGRRSVPEGGYPELTPSPQQNVTEVPVVGDYLQGKRR
**PREDICTED: uncharacterized protein LOC106068784** [*Biomphalaria glabrata*] (100%);<br>Sequence ID : XP_013069827.1<br><br>Domains:<br>-Signal peptide: 1-21<br>-Disintegrin and metalloproteinase: 90-333	LGUN_random_Scaffold286: 70002-70112; 67477-67586; 65313-65458; 63714-63819; 62425-62557; 60967-61102; 60466-60602; 58410-58575; 53454-54138	MWSPAVILCFALLVLTKSCQGQTYVLEAYIVADDSAVQNQVREIATTESYSVKYTKAVANLRKDIDYIVT<br>QVNVLLGSLSQNGVTMEMRIRRLDILSGNIITTFMPGTSYVVDSIVARNSFQNWLNLQNAYANINYDFAL<br>LWTGFNLYGTSGLADNSITNVAQMCGAAGITVVEFDPTAREAVATARAIGNLLGASNDGALSTSIMAIIN<br>APGDVNRWSYSTCSASSIKDFIKLPKASCLLTTNIASTKPSVTFSSYTGNLFDPDIICQRALNDSRTYMC<br>KSLPQIYNNLLPKGNAVCGQIFCRIPNTMTCTPVYTSDGLVCDNQKRCSLGQCSSYSTPVVDTNCVFGDQ<br>KKLEFPAVPFYGTCQAFISQYGAVNCYSSPINESCCSTCKSYNNGRVGCEYGDRSLDCVKYTKASICPTY<br>KNTLCCGYCYNYVGKRSPSDEPVPKLNITLPKIIGQEPDIMPFEEFNSQP

**PREDICTED: ADAM family mig-17-like isoform X2 [Biomphalaria glabrata]** (44%) XP_013069827.1		
**PREDICTED: ADAM with thrombospondin motifs 9-like** [*Biomphalaria glabrata*] (100%) Sequence ID: XP_013075983.1<br><br>Domains:<br>-Metallopeptidase :36-152	LGUN_random_Scaffold16032: 213-334; 1737-1893; 3892-4003; 5778-5914; 7097-7228; 10218 -10401	MYGVNVEARIKKLDILSSNIIPSSALLPGTENVAPSENAMATFENWLVAQNSYNNIQYDFAIFWTGYDLR DAEDLEGRTHIGTICDSRYAVSMSEFDRTYHTALLTAQQISELLGSQHDGVLSSITNRWFFSSAVAGDIK NKLASLSPNCLLTTSATSTKPFVEVYEYDGHILNPDITCQRRLNYTNSYMCTGWHLYNNQATGGDRVCTT IYCNEMDEVNCKRIETPEGMICESGKRCRHGSCVVDIHTPTNIDPSCVFGDQKEVTLGNFTGTCHEYIRM FSARVCYNSFVNQVCCMSCKAHHTGSTGT
**PREDICTED: Serpin B6-like protein** [*Biomphalaria glabrata*] (100%); Sequence ID: XP_013077880.1<br><br>Domains:<br>-Signal peptide: 1-27<br>-low complexity: 50-61/ 63-76<br>Serpin: 92-458	LGUN_random_Scaffold1827: 27197-27439; 31889-32190; 39463-40100; 39670-40571; 39564-40002	MNTSRILVLVVFLVLLAVIALTKRADAGDEQELSAAKNEISRYRRTLLSRNRGGNRDRDNRTKQGKKKGN NSKKQKLSPEERIGVANSHVALNVFTSLYPSINSNTIFSPHSLHTALAMTMLGAKKDTKKEFYISLGLKA AKVGKSEVHGIYKKLLQSLSSRPDIILSYANSVYVKSGLELKKKFRTGLTTHYHAKVDNFNMTDPEGPER SINAWVANATRGKISDILKKGDVNINTAMIIINAVYLNASWAIQFQTFNTKPKPFYSLNSGVQTIPTMHI VQFYNYANHHDTEVIELNFNGSQLAMYILLPSAKSNLNQLLQNMKASSALANPFDELLTGLERRNLNLSL PTFKIQSDVIDLVPALKNLGLKKAFNSSQADFKGIAKQKLFLDKVIQKAVISVNETGCEAAAATDVIFGL KSSRPTPALEVKVDRPFLYVIRSKAQKVVLFMGTFVGV
**PREDICTED: 14-3-3 protein epsilon-like** [*Biomphalaria glabrata*] (100%); Sequence ID: XP_013082420.1<br><br>Domains:<br>-14-3-3: 4-245	LGUN_random_Scaffold260: 178321:180720: BGLB007883	MADREDNVYQAKLAEQAERYDEMVSSMKKVAQQDTELTVEERNLLSVAYKNVIGARRASWRIVSSLEQKD EGKVSEEKQQITREYRKQIESELNSICHDVLSVIDNNLIPNAATGESKVFYYKMKGDYHRYLAEFATGND RKEAAENSLVAYKAASDISMTELAPTHPIRLGLALNFSVFYYEILNSPDRACRLAKAAFDDAIAELDTLS EESYKDSTLIMQLLRDNLTLWTSDMQGENDGGEQEQQTVQDIEGEDAS
**PREDICTED: transforming growth factor-beta-induced protein** [*Biomphalaria glabrata*] (100%); Sequence ID: XP_013079848.1<br><br>Domains:<br>-Signal peptide: 1-17<br>-FAS1 (fasciclin-like): 59-158/ 195-296/ 376-476/ 513-614	LGUN_random_Scaffold209: 176383-176605; 177276-177462; 179882-180008; 180681-180831; 188684-188904; 189702-189891; 192064-192299	MKYFCLVITVVISLTKGQTVTPFPDVVSALTASGHFETFLDLLKPSGLLERINSSSHFTLFAPTDDAFAK LPADTFNALKSDPIQLALVLGYHVVLSSSFKAGTQQDTVLKSSNGLPIRINTYSLIHSNAASGVNITLKN IPVVHGYIQGVNTVLNPPIGNIVQIAMNRTDLKTFTSWVVSSNLVQFFLNDDDVTLFVPNDDAIKKLSPT TLDYLNSHPSALADVLKYHVMSDYTLFTIGMTHSMTLKSADQHHDLLMILEDGNGGLFVNHAKIVERDIA CIEGVLHIIDNVLIPPHVLVAIQDTSVAVGKQQIMKSIYLLLAVLLLKRGSAQTTEAPPLPDVEDVLAAD SRFTTFYQLLKTTDLLEEINETNHFTIFAPTDAAFAKLPAGSLDALKADVDKLKETIGYHVVLNSSYHVH GSQQDSILKSSTQLPIRINTYSLAHTVTAEGVNITIRNISVNHGYIQGIDGVMKPPTGNVVDLGTTRSDI STFESLLVKANQTAYFTSDHSTTLFIPTDDAFKKLSAETLDYLNNHISDLTDVLRFHYVKQYSLYSLGMK HAFTIQSADHSHDYLMLLEDDNGGMRVNQAKIIEKDISSINGVIHIIDDVLIPPRVLVAIRDQSIVVG
**PREDICTED: neo-calmodulin-like protein** [*Biomphalaria glabrata*]	LG27_random_Scaffold161: 16903:18554 :BGLB001498	MAEQLTEETIAEFKEAFSLFDKDGDGTITTKELGTVMRSLGQNPTEAELQDMINEVDADGNGTIDFPEFL TMMARKMKDTDTEEELREAFRVFDKDGNGFISAAELRHVMTNLGEKLTDEEVDEMIREADTDGDGQVNYD GECHSASYHMLILHLPRGFHPPQRSS

(100%); Sequence ID: XP_013067017.1<br><br><u>Domains:</u><br>- EF-hand, calcium binding motif: 12-40/ 48-76/ 85-113/ 121-150		
**PREDICTED: GATA zinc finger domain-containing protein** [*Biomphalaria glabrata*] (100%); Sequence ID: XP_013076718.1<br><br><u>Domains:</u><br>-Signal peptide: 1-25<br>-Cu-Zn SOD : 352-497<br><br>**Superoxide dismutase (Cu-Zn)** [*Megathura crenulata*]<br>CAF22060.1 (40%)	LGUN_random_Scaffold1689: 27317 -27807; 36361 -36477; 37754-38211; 52024-52107; 66296-66541; 76688 -76877	MKPMQMFNLRVLFFVSILVILSVHCQKFSKKGKHLNEQYKPNLKIKENKFINIGGNVSLNDTIEQNITSE<br>ETQDNKENMNETISLKTVNAKENVTSVETQTMTSQIEFLNASNHISDLDIVNITQDETLSNSSESYKKPS<br>TSCDCHVHHLMDKLKKDLSSMRTYVKERHNPIQIHIYKGEKIKSSQMSEPSKDNDKKELGKGKTKDQSKS<br>YSKQLNKNDDDNLQDHVHVHSKGNLRHNHSKYNHIHSLNNQDLRRNYQNGHSNVKYKPKNTKFHNQITHD<br>HYEPSVSRQRHFKVQETHIHLNIQVSGRNEEDAKEKERHKMSVEQIKKEGLLEKDLTAKNEIPIFARCDM<br>KTYNSGHNISGTVFLHQLPGQYLKVEVQLHGFSVQSNHRSTDIKEEKQFLNLSSNDISLNKQLHGFHVHE<br>FGDSSKGCHSMGGHFNPEGVHHGYRNNSHGHVGDWGNIDVDMYGDSNSNFTVQAASLIGPNSILGRGIVV<br>SLHAITHKELLECEKVDFG
**PREDICTED: uncharacterized protein LOC106059379** [*Biomphalaria glabrata*] (100%);<br>Sequence ID: XP_013072448.1<br><br><u>Domains:</u><br>-Copper type II, ascorbate-dependent monooxygenase: 1-100	LGUN_random_Scaffold1196: 16772-16869; 15959-16119; 14884-15048; 11587-12745	MSIQLYRNKVLIYNLTNDELYNLDAPVTHYPKQHVQILPGDEIITRCVYNTTSAATSDPSGQVQGEMCYG<br>YLNVYPQSALRIPDGTCVASTSLSYCERSQSIPVDGCDWKTFLSYGDLELIDMYCQLRSSCQLQGSCKRV<br>QSSQNNASSPSHDFSSILSIDDTDSISMKSNVTDGRVSKGSTDNISATQNNTNDTFNSQSIPSDNSIVPN<br>ISLFDVNTQNTTYSSSLIEANTTSVNHSASLADASIVKILSSDSIGNINSQVENYFNETIKHFAGKMSVN<br>QTASNDLDLLETKRQDTTSNITSTAHLGGVQLNATLSAKDKSVAETRNSSNGLTSTYHNSDTGHISTTHI<br>NNKGQISSTSVLDLGHVSTSFIDSNGAICTVYIDSNGNVSTSYNDGNGRVSTTHIDRKGQISTTHLDSDG<br>HISISRIDSKGLVSTIRTDSKGRNSKRDDTSVEVGSYTYGDSIGHVEPKPSVRDNETASQSLELSFNPKD<br>IFHNKAISVHKVACYLSFYMCLIQVIIVTLRLL
**PREDICTED: uncharacterized protein LOC106079481** [*Biomphalaria glabrata*] (100%); Sequence ID: XP_013096101<br><br><u>Domains</u>:<br>-EF-hand, calcium binding motif : 44-72/ 105-133<br><br>**Calcium binding protein 1** [*Biomphalaria glabrata*] (37%); Sequence ID: AAV91525.1	LGUN_random_Scaffold6449: 3374-3477; 6563-6717; 7143-7387; 8008-8087	MMFLLLALIPALALAQTTADLTVTIDGVFDALDYDFDNEIARDELTKFFQTYDANHDNRISRADYTAAID<br>ENFPNETHDPIITNAYRNLFDVLDNNKNGFIEPNDLDAVFRNADDNNNGVVTRAEFHKYFNEIFIFLVLG
