# Supplementary material for: Proteomic analysis of Biomphalaria glabrata plasma proteins with binding affinity to those expressed by early developing larval Schistosoma mansoni
Source: PLoS Pathog. 2017 May 16;13(5):e1006081. doi: 10.1371/journal.ppat.1006081 (PMC5433772; doi:10.1371/journal.ppat.1006081)
Supplement: S6 Table — Protein identifications (Sequence ID) were determined using Mascot and Sequest against the current B. glabrata annotated NCBInr database. The numbers following the Scaffold idenfications (:xxx-yyy) designate the location of the coding gene sequence within the designated scaffold. Peptide sequences in red represent the original peptides identified in the tandem MS analyses of plasma proteins. (PDF) [file ppat.1006081.s007.pdf]

S6 Table. Protein sequences, identified domains and genomic locations in VectorBase of other immune-related NMRI and BS-90 *Biomphalaria glabrata* plasma proteins eluted from larval transformation protein (LTP) affinity columns.

Protein ID	Scaffold No.	Predicted Protein Sequence
**PREDICTED: Lipopolysaccharide-binding protein-like** [*Biomphalaria glabrata*] (100%);<br>Sequence ID: XP_013070669.1<br><br><u>Domains:</u><br>-Bactericidal permeability-increasing protein (BPI)/LPS-binding protein (LBP): 1-193/ 180-384	LGUN_random_Scaffold1023: 50441-50613; 50252-50331; 49394-49526;46627-46718; 45543-45623; 43433-43591; 40841-40951; 39401-39593	MLLINLLGHYFNSFRLENVAIPDIDMSFNPVQRGLTMNLRNLGVDVLLDYRVRYKLLFVPISNSGGVSVN<br>FRGVELTATVGIDQFEDTSPRLVSRSCSAAIGDFNINFSGSMAWLLNLLKGLFRGKIRDTIRSKICDKVT<br>EAINTQGADKLRQIPLESDIADKFVIDYRLLSPVLFTDSYLETQHKKLYKRGTLGYAAHTHGYLQYVISP<br>DKLPAQSKGFLNTTCPDSACAGTLIPLLAEKYPNSFVTLTISSSKAPDFNITSNGLGLTLYGTLDLSTTS<br>ASAVVTANVVISIAGAAFVRNDRIVGNVAQFRTVLADIDSQRLISLYTFQISSINKALDDGMNMVVIPQL<br>NAVGDTGIALPVLKDLTFQNPRIVYQDKSVLISTNVQYQGIV
**Thioester-containing protein 1.5 (TEP1.5)** [*Biomphalaria glabrata*] (100%); Sequence ID: ADE45333<br><br><u>Domains:</u><br>-Signal peptide: 1-21<br>-Proteinase-binding alpha-macroglobulins: 126-219/463-599/718-808/978-1241<br>-Thiol ester: 929-958<br>-Alpha-2-macroglobulin-receptor: 1342-1426	LGUN_random_Scaffold4524: 9507-9566; 9149-9332; 8894-8916; 4705-4867; 1865-1933; 1610-1736;<br>LGUN_random_Scaffold10725: 6705-6750; 4397-4481; 2567-2681; 2042-2159; 1413-1527;<br>LGUN_random_Scaffold9700: 5915-6144; 3125-3226; 2777-2839; 709-909;<br>LGUN_random_Scaffold8482: 12144-12290; 10814-10917; 10616-10675; 10307-10337;<br>LGUN_random_Scaffold2300: 37595-37626;<br>LGUN_random_Scaffold76986: 250-267;<br>LGUN_random_Scaffold8482: 10166-10290;	MRMKLNLILFVFYLVFQECQGGKYFISAPRNVVPGTAYDISVDILKQDIGNVTVEAILQDYSFSIPEGPK<br>SLLTANGTFSPGVRGTLSMPIDFNLHCSYCRILLKGYNPLQFEQDIFIQISSDILSILIQTDKAIYKPKE<br>RVNFRILAVYYNLQLYTGTFHYEILDPYDNKINVLSGVSGTFGVVEGFFDLSDQPSFGTWKINVRTETVS<br>GAESQFFEVAEYDLPRFQVDVGLPPFALLSDTTLSGSVEAKYTFGQPVYGLVLLQIGENVDTIDKCNVNR<br>KVTEISFEIKGKGNFSVPLEDIRRSVHLNEKKKIKITAFVIEASTGIKLNGSSVITYYGNRYQIKFLEMT<br>PAVFKPGLQYTAYVQVTTPDGLPPTESNLSLSVYTSVTYQMTVPDQELYSPSSFSGSYPLPGQNMSLPAN<br>GILSIDIDIPLNATSIDIKVSLNKQTTAEKRISKSYSMSNNYLQLSLLSKLVKAESDVLIKITSTEAIDS<br>LAYEIRSRSDHVKSGVLELNGQREFNATFKVGPSWAPIAQLLMYYIRRDSNEIVTDSLAFNVEGMFENKV<br>NVAFKENETDINKNVSLELSADSDSQIYVLAVDQSVLLLKTGNDLTPNKVKDSFISKFHKGEVPTDSNFA<br>LSYSGSSINEVFSNMGLVIATDLNIFAPFRPIALGRFPSSGFDRQGMMGAPMAMSFRDDNAMESASFEMD<br>VATSTKPVERVRSFFPESWLWTSVKSINGHATLTTTVPDTITSWIVSAFATNSDTGLGVAPTTSKLRVFR<br>PFFVSLTYPRSVTRNEQFIVQATVFNYLPVDLMVTVSLKENPFLTPITPGPGNQASNIQVRANEQGIVYF<br>SLSALTVGSLDTEVSARSNMAADAIVRQILIKHEGAPVVYNNPILINLSNNQSTFEKNIAFTLPDSLVPE<br>SQRIRVKVTGDLIGSTVQSLTSLLTLPTGCGEQSLVKFTPNIHIGRYLKATNQLSEELNKKIIDLLNDGY<br>QRQLTYKRYDNGFSAFGNYDISSSTWLTALVVTSFAEAQEFIFVDKEIILKASMLLIDRQNIDGSFNEFG<br>KVLDRNTQGTTTAGPALTAFVLVALLKAKELADVQDCKNNNKCRYYLLGNATLNATRNLERLMLADSIDD<br>QFSLAVASYALAEAKSQLAQSTFEKLLTFVKQEGGLEYWSANSTVNNEELNRFINWRPPRLQARPIDILI<br>TSYAILTYSSLGRLDEALPSVRWLTLQKNAQGGFVSTQDTVVGLQALSFYGSKSFRPDTNITIYVSDMNT<br>HLTMNVNSENALSLQIQEIQSNSQDFSITASGSGLALLDIEYSFNVLKELSKPVFDANTVLLDDKLDSFN

	LGUN_random_Scaffold7766: 10058-10180; 9732-9849; LGUN_random_Scaffold45951: 828-903; LGUN_random_Scaffold7766: 277-407; LGUN_random_Scaffold53075: 386-529; LGUN_random_Scaffold6063: 31434-31613; 29915-29997; 25214-25375; 23259-23332; LGUN_random_Scaffold7260: 8533-8740; 5514-5738; 11544-11696; 11926-11989; 12187-12304; 13837-13988; 15855-15963; 16394-16478	IMVCTKFLMKHDTGMVVQELSIPSGFVPDLSTLGQVAGVKRSERKGSIVAIYFDKISGSSLCYSIVMTRE AKVAKSQKSYVRTYDYYEPANQATVFYQPRTLRDSTVCDVCLNCCP
**Thioester-containing protein (TEP)** [*Biomphalaria glabrata*] (100%); Sequence ID: ACL00841.1<br><br>Domains:<br>-Signal peptide: 1-21<br>-Proteinase-binding alpha-macroglobulins: 126-219/463-599/718-808/978-1241<br>-Thiol ester: 929-958<br>-Alpha-2-macroglobulin-receptor: 1342-1426	LGUN_random_Scaffold4524: 9507-9566; 9149-9332; 8894-8916; 4705-4867; 1865-1933; 1610-1736; LGUN_random_Scaffold10725: 6705-6750; 4397-4481; 2567-2681; 2042-2159; 1413-1527; LGUN_random_Scaffold9700: 5915-6144; 3125-3226; 2777-2839; 709-909; LGUN_random_Scaffold8482: 12144-12290; 10814-10917; 10616-10675; 10307-10337; LGUN_random_Scaffold2300: 37595-37626; LGUN_random_Scaffold76986: 250-267; LGUN_random_Scaffold8482: 10166-10290; LGUN_random_Scaffold7766: 10058-10180; 9732-9849; LGUN_random_Scaffold45951:	MRMKLNLILFVFYLVFQECQGGKYFISAPRNVVPGTAYDISVDILKQDIGNVTVEAILQDYSFSIPEGPK SLLTANGTFSPGVRGTLSMPIDFNLHCSYCRILLKGYNPLQFEQDIFIQISSDILSILIQTDKAIYKPKE RVNFRILAAYYNLQLYTGTFHYEILDPYDNKINVLSGVSGTFGVVEGFFDLSDQPSFGTWKINVRTETVS GEESQLFEVAEYDLPRFQVDVGLPPFALLSDTTLSGSVEAKYTFGQPVYGLVLLQIGENVDTIDKCNVNR KVTEISFEIKGKENFSVPLEDIRRSVHLNEKKKIKITAFVTEASTGIKLNGSSVITYYGNRYQIKFLEMT PAVFKPGLQYTAYVQVTTPDGLPPTDSNLSLSVYTSVTYQMTVPDQELYSPSSFSGSYPLPGQNMSLPAN GILSIDIDIPLNATSIDIKVSLNKQTTAEKRISKSYSMSNNYLQLSLLSKLVKAESDVLIKITSTEAIDS LAYEIRSRSDRVKSGVLELNGQREFNATFKVEPSWAPIAQLLMYYIRRDSNEVVTDSLAFNVEGMFKNKV NVAFKENETDINKNVSLELPADSDSQIYVLAVDXSVLLLKTGNDXTPXKVKDSFXSXFHKGAIPTDSNFA LSYSGSSIXEVFSNMGLVIATDLNIFAPFRPIALGRFPSSGFDRQXMMGAPXAMSFXDDXAMXSASFEMD VTTSTKPVERVRSFFPESWLWTSVKSINGHATLTTTVPDTITSWIGSAFATNSDTGLGVAPTTSKLPGFR PFFGSLTYPRSVTRNEQFIVQATVFNYLPVDLMVTVSLKENPFLTPVTPGPGNQASNIQVRANEQRTVYF SLSALIVGALDIEVSARSNMAADAIVRQILVRHEGAPVVYNNPILISLSNNQSTFEKNIAFTLPDSLVPE SHRIRVKVTGDLIGSTVQSLTSLLTLPTGCGEQSLVKFTPNIHIGRYLKATNQLSKELNKKIIDLLNNGY QRQLTYKRYDNGFSAFGNYDLSSSTWLTALVVTSFAEAQEFIFVDKEIILKASMLLIDRQNLDGSFNEFG KVLDRNTQGTTAGPALTAFVLVALLKAKELADVQYCKNNNKCRYYLLGNATLNATRNLERLMLADSIDDQ FSLAVTSYALAEAKSQLAQSTFEKLLTFVKQEGGLEYRSANSTVNNEELNRFINWRPPRLQARPIDILIT SYAILTYSPLGRLDEALPSVRWLTLQKNAQGGFVSTQDTVVGLQALSTYGSKSFRPDTNITIYVSDMNTH LTMNVKSDNALSLQIQEIQSNSQDFSITASGSGLALLDIEYSFNVLKELSKPVFDVNTVLLDDKLDSFNI MVCTKFLLKHDTGMVVQEVSIPSGFVPDLSTLGQVAGVKRSERKGSIVAIYFDKISGSSLCYSIVMTREA KVAKSQKSYVRTYDYYEPANQATVFYQPRTLRDSTVCDVCLNCCP

	828-903;<br>LGUN_random_Scaffold7766: 277-407;<br>LGUN_random_Scaffold53075: 386-529;<br>LGUN_random_Scaffold6063: 31434-31613; 29915-29997; 25214-25375; 23259-23332;<br>LGUN_random_Scaffold7260: 8533-8740; 5514-5738; 11544-11696; 11926-11989; 12187-12304; 13837-13988; 15855-15963; 16394-16478	
**PREDICTED: CD109 antigen-like** [*Biomphalaria glabrata*] (100%);<br>Sequence ID: XP_013071291.1<br><br>Domains:<br>-Proteinase-binding alpha-macroglobulins: 47-138/ 348-500/ 649-740/ 918-1168<br>-Thiol ester: 869-898<br>-Alpha-2-macroglobulin-receptor: 1271-1355	LGUN_random_Scaffold108: 84237-84416; 83387-83461; 82641-82779; 80795-80861; 78882-79106; 78232-78463; 76389-76589; 76048-76202; 75142-75294; 51880-52111; 41664-41787; 35799-36007; 39854-39999; 39487-39666; 39487-39666; 39487-39666; 38763-38921; 36931-37116; 35799-36007; 34930-35082; 32766-32916; 31828-32071	MESLHFLFYSQLPADLPSSTYTLNVKGSGGLTFDKSENLNYNNKETSVFIQLNKAIFKPGDTVNFRVFGV<br>YSDLKSYTDPIDISIYDANSNKIKQWLKVTPTNGVITQELTLSTQPVLGDWKISVDAGRTKEEKVFTVAE<br>YVLPKFEVDVVTPSYALTTDNDVTVTVKSKYTYGKPVNGTADVLVKLHESFNTFDYSRALPVTTLQVPLN<br>GEAKVIIPMSQVKAISANLNQHVLIVIANVTESLTGNQMSGNGTVTLYDKGVKLDFPASNPKTFKPALQY<br>IAYLKVTQPDGLPMTSTAEQVKVSIRVTAELPGTTPTPYYWYVPPTESRDLPALSLAIPDNGLVAIPVDV<br>PADAKDVHVTANFQGVSKELTLGKSHSPSNSYIQLILKSGSVIKFQILSRGGIVKTGTVDANGQLVYQFS<br>IPSDSSMAPNARIVLYYVRADGEIVTDSISFDISGAFKNKVSIDLDKTDVEPGDDVTVTVKADPDSTAYC<br>LAIDQSVLLLKGGNDVTDNDVYTELKEYDTITESGSNKGIIDCPMCKRRKRMIWWPFPTYYGGSDAQQIF<br>SNAGVVVLTDATVYHYQEPIHLFNIPNFFQCGRSLNGQLRKRCFASFSPVMNLVSSVVSDPITLPVTETE<br>TKTEDLQQPTKTRSNFVETWLWNSLDIGANGSASITATVPDTITSWVASAFAINSASGLGVAPTQAHLRV<br>FRPFFVSLNLPYSVTRGEHLALQANVFNYMTEDMQVRVTLAKSDNFFNIEIDANGAEVLKQVESVQDVMI<br>KAGEAKSVYFPIVPADLGKIDIEVKAQSTKAADAVRRQLLVEAEGVPKIYNVPVLIDLTEGKTSFSKTVD<br>LTLPSNTVKGSELARISAVGDLMGPTIAGLDSLLQMPTGCGEQTMIGLAPDVYVTDYLKSVNQLSGDIQT<br>KALSYMESGYQRELTYKHTDGSFSAFGNSDASGSMWLTAFVTRVFKQAKAHIYIDDEVLIKAIQWMVSKQ<br>NANGSFPEPGNVIHKNMQGQAGSGVGLTLFVLISLLENKDLLVNTNAAGILVDEARQKALVYAEQEVAKT<br>DDLYILNMAAYAFQLANSSQVQTVLSKLEQKATVKDGRKYWHQPEQPKTTNTWDYPNPTKAVDIEMTSYA<br>LLTYAARGNIVAGKSIMQWLTEQRNSNGGFSSTQDTVLALNALSEFAKQTYSNNFNVQITTQLNATTSYT<br>FNIDKTNSLLLQSRETPNVPSQVKIDATGSGMALVQVAVSFNVESEIFETTFDLTVKLIEETINNLFVET<br>CAKWLGSGPSSAMAVQEIGIPSGFEADIESIPQLDILKRIETQNKKVILYFDQIGTTPVCLNFRAVRTGL<br>VAKSQPAAIRVYDYYEPRNQVTAFYQSQILKDSTVCDVCKECDNCV

**PREDICTED: complement C4-like** (LOC106078805), [*Biomphalaria glabrata*] (100%);<br>Sequence ID: XP_013095289<br><br><u>Domains:</u><br>-Alpha-2-macroglobulin-receptor: 12-96	LGUN_random_Scaffold6063: 21546-21671; 19546-19697; 18165-18273; 17575-17738 / LGUN_random_Scaffold7260: 12179-12304; 13837-13988; 15855-15963; 16394-16557	MVCTKFLLKHDTGMVVQELSIPSGFVPDLSTLGQVAGVKRSERKGSIVAIYFDKISGSSLCYSIVMTREA<br>KVAKSQKSYVRTYDYYEPANQATVFYQPRTLRDSTVCDVCPNCCP
**Biomphalysin** [*Biomphalaria glabrata*] (100%);<br>Sequence ID: AGG38744.1<br><br><u>Domains:</u><br>-Signal peptide: 1-17<br>-Aerolysin: 195-531	LGUN_random_Scaffold10:1097564:1099282: BGLB000137	MFLQIFVAVTLVQYVSSQCTYSSWWYSFDTPGQSKCNEINSYINALDRNDVNWADDALSNLEGVQCCRPP<br>APWNNVEQQVVYEDWTATLDSDYTWAFCRVGYFLQGLYRSDTGWPRFKGYLFNLESARCTKPANHPLNYG<br>TCQDIDVSSCMGRKGQCSCPGGYFLTGLYRADGDDLYFLKKIRCCTPAAKPLEMDEKSKIQTRIMDTTLW<br>NMATLAHYMGYGWCYGCHGLAVGEDFTRNGFTWAADTRTFWGKWCEGDKNGERLNLVFGDWGFAVKEIIY<br>GKSVIEDLQAESVDSGVLYNRASSPVTESIERSKTIQETITHSTTSTFTNSHGLGVELEFEIASVKGKAS<br>YKTRFEYSTSTTNSKSISETQGFTKQSSITLGPMEGAKYEVIMSKSRTTVPYTAIITTKFSTEMKGFLRW<br>EDGNGNFHQDYRTNSGRPTFNYRFGDSSVPFYKALKKQSDNNEGVWMWGMLFQKFPDARRVTNRLTDETQ<br>YQFTLAGKLEKVEGTSVNVKWEKMKLNRRDVSGNDEPGSNITTYIAASGPADKPAVVEYPKVNLNNKEPF<br>KPIEIPVTEVKV
**Dermatopontin 2** [*Biomphalaria glabrata*]; (100%); Sequence ID: AAZ80786<br><br><u>Domains:</u><br><u>-Dermatopontin: 11-156</u>	LGUN_random_Scaffold8114: 1204:2972: BGLB013420	MASVASAAYVNDWDQPFNFRCPDGQVVSYVSSIHNNRREDRRWEFLCRSTRQTHSCTDSGYVNDFDGPLV<br>YTCPGNKVMVGVHSYHNNRREDRRFGFYCCDVQGSTPRDCYTTNYVNDWDEKLTLVVPEGTAVKAAYSHH<br>DNRREDRRWQFQICTL
**PREDICTED: Millepora cytotoxin-1-like** [*Biomphalaria glabrata*] (100%);<br>Sequence ID: XP_013061974.1<br><br>Domains:<br>-Signal peptide: 1-24<br>-Dermatopontin: 29-174	LGUN_random_Scaffold7789: 3993-4096, 5800-6107, 8666-9418	MPRMYERYISIVLCSLLVLNTVHCVTYQTEEQEDWTVECAPNHVITDIVSDFFFSDRLWNITCAPIRPKI<br>NITECVWSNYHNAPQYDFLFDCPMNSILAGISSFHRSRDRVFKFYCCTNKDYLVHACQHTLTINNAKGFI<br>SFRVPEAMYVRGLFSSYDSTAGDRLYRIHLCKLDPADPTVECPTTELTTQAVTKLVNQTETPLLESESED
**PREDICTED: uncharacterized protein LOC106068782** [*Biomphalaria glabrata*] (100%);<br>Sequence ID: XP_013083706.1<br><br><u>Domains:</u>	LGUN_random_Scaffold286: 44298-44384; 42897-42974; 39464-39607; 36011-36117; 34918-35050; 34015-34151; 32322-32458; 31272-31438; 29557-29654; 27862-28646	MVRRIAVLLCLSILVMTSSCQVQNLVAEVYVVVEDSTVQSYAQQSSPAQTIAEATNSLRNDIDLVITQTN<br>VLLSSLVQYGINIEVRKRKVDILSTNIINTFLLETTYIADSSAAKASFQSWLNLQNAYSTLNYDFAVLWT<br>GFDLFGTSGIFENSITSFAAMCKSTGVSVMEFDPTARDIVATAKIIAYLLSSLNDGITSTYIMAIINAPD<br>DANRWFYSNSSAQSIKEFVALPRASCLLSTNSASVKPAVTYATYTGSLLDPDVICQRSLNDSRSYMCKSL<br>PQIYNNQPPKGNAVCSQIYCRIPKTSICTPAFTSDGMICENQKRCTKGQCSSYPTPSVDPNCLWGDQKTL<br>DYPLIPFSGTCKPFLDTYGAANCYTRPINETCCNTCKTYNSGIPGCEYGDKSSACVLFTQAQVCPIYKNT

-Signal peptide : 1-20<br>-Zn-dependent metalloprotease, ADAM- or reprolysin-like subgroup: 31-228<br><br>**PREDICTED: ADAM family mig-17-like isoform X2** *[Biomphalaria glabrata]* (42%); Sequence ID: XP_013069827.1		LCCNYCRNYVGRRSVPEGGYPELTPSPQQNVTEVPVVGDYLQGKRR
**PREDICTED: uncharacterized protein LOC106068784** [*Biomphalaria glabrata*] (100%);<br>Sequence ID: XP_013083707.1<br><br>Domains:<br>-Signal peptide: 1-21<br>-Disintegrin and metalloproteinase: 90-333<br><br>**PREDICTED: ADAM family mig-17-like isoform X2** [*Biomphalaria glabrata*] (44%); Sequence ID: XP_013069827.1	LGUN_random_Scaffold286: 70002-70112; 67477-67586; 65313-65458; 63714-63819; 62425-62557; 60967-61102; 60466-60602; 58410-58575; 53454-54138	MWSPAVILCFALLVLTKSCQGQTYVLEAYIVADDSAVQNQVREIATTESYSVKYTKAVANLRKDIDYIVT<br>QVNVLLGSLSQNGVTMEMRIRRLDILSGNIITTFMPGTSYVVDSIVARNSFQNWLNLQNAYANINYDFAL<br>LWTGFNLYGTSGLADNSITNVAQMCGAAGITVVEFDPTAREAVATARAIGNLLGASNDGALSTSIMAIIN<br>APGDVNRWSYSTCSASSIKDFIKLPKASCLLTTNIASTKPSVTFSSYTGNLFDPDIICQRALNDSRTYMC<br>KSLPQIYNNLLPKGNAVCGQIFCRIPNTMTCTPVYTSDGLVCDNQKRCSLGQCSSYSTPVVDTNCVFGDQ<br>KKLEFPAVPFYGTCQAFISQYGAVNCYSSPINESCCSTCKSYNNGRVGCEYGDRSLDCVKYTKASICPTY<br>KNTLCCGYCYNYVGKRSPSDEPVPKLNITLPKIIGQEPDIMPFEEFNSQP
**PREDICTED: Serpin B6-like protein** [*Biomphalaria glabrata*] (100%);<br>Sequence ID: XP_013077880.1<br><br>Domains:<br>-Signal peptide: 1-27<br>-Low complexity: 50-61/ 63-76<br>Serpin: 92-458	LGUN_random_Scaffold1827: 27197-27439; 31889-32190; 39463-40100; 39670-40571; 39564-40002	MNTSRILVLVVFLVLLAVIALTKRADAGDEQELSAAKNEISRYRRTLLSRNRGGNRDRDNRTKQGKKKGN<br>NSKKQKLSPEERIGVANSHVALNVFTSLYPSINSNTIFSPHSLHTALAMTMLGAKKDTKKEFYISLGLKA<br>AKVGKSEVHGIYKKLLQSLSSRPDIILSYANSVYVKSGLELKKKFRTGLTTHYHAKVDNFNMTDPEGPER<br>SINAWVANATRGKISDILKKGDVNINTAMIIINAVYLNASWAIQFQTFNTKPKPFYSLNSGVQTIPTMHI<br>VQFYNYANHHDTEVIELNFNGSQLAMYILLPSAKSNLNQLLQNMKASSALANPFDELLTGLERRNLNLSL<br>PTFKIQSDVIDLVPALKNLGLKKAFNSSQADFKGIAKQKLFLDKVIQKAVISVNETGCEAAAATDVIFGL<br>KSSRPTPALEVKVDRPFLYVIRSKAQKVVLFMGTFVGV

**PREDICTED: neo-calmodulin-like protein** [*Biomphalaria glabrata*] (100%); Sequence ID: XP_013067017.1<br><br><u>Domains:</u><br>- EF-hand, calcium binding motifs: 12-40, 48-76/ 85-113/ 121-150	LG27_random_Scaffold161: 16903:18554 :BGLB001498	MAEQLTEETIAEFKEAFSLFDKDGDGTITTKELGTVMRSLGQNPTEAELQDMINEVDADGNGTIDFPEFL TMMARKMKDTDTEEELREAFRVFDKDGNGFISAAELRHVMTNLGEKLTDEEVDEMIREADTDGDGQVNYD GECHSASYHMLILHLPRGFHPPQRSS
**PREDICTED: GATA zinc finger domain-containing protein** [*Biomphalaria glabrata*] (100%); Sequence ID: XP_013076718.1<br><br><u>Domains:</u><br>-Signal peptide: 1-25<br>-Cu-Zn SOD : 352-497<br><br>**Superoxide dismutase (Cu-Zn)** [*Megathura crenulata*] (40%); Sequence ID: CAF22060.1	LGUN_random_Scaffold1689: 27317 -27807; 36361 -36477; 37754-38211; 52024-52107; 66296-66541; 76688 -76877	MKPMQMFNLRVLFFVSILVILSVHCQKFSKKGKHLNEQYKPNLKIKENKFINIGGNVSLNDTIEQNITSE ETQDNKENMNETISLKTVNAKENVTSVETQTMTSQIEFLNASNHISDLDIVNITQDETLSNSSESYKKPS TSCDCHVHHLMDKLKKDLSSMRTYVKERHNPIQIHIYKGEKIKSSQMSEPSKDNDKKELGKGKTKDQSKS YSKQLNKNDDDNLQDHVHVHSKGNLRHNHSKYNHIHSLNNQDLRRNYQNGHSNVKYKPKNTKFHNQITHD HYEPSVSRQRHFKVQETHIHLNIQVSGRNEEDAKEKERHKMSVEQIKKEGLLEKDLTAKNEIPIFARCDM KTYNSGHNISGTVFLHQLPGQYLKVEVQLHGFSVQSNHRSTDIKEEKQFLNLSSNDISLNKQLHGFHVHE FGDSSKGCHSMGGHFNPEGVHHGYRNNSHGHVGDWGNIDVDMYGDSNSNFTVQAASLIGPNSILGRGIVV SLHAITHKELLECEKVDFG
**PREDICTED: uncharacterized protein LOC106059379** [*Biomphalaria glabrata*] (100%); Sequence ID: XP_013072448.1<br><br><u>Domains:</u><br>-Copper type II, ascorbate-dependent monooxygenase: 1-100	LGUN_random_Scaffold1196: 16772-16869; 15959-16119; 14884-15048; 11587-12745	MSIQLYRNKVLIYNLTNDELYNLDAPVTHYPKQHVQILPGDEIITRCVYNTTSAATSDPSGQVQGEMCYG YLNVYPQSALRIPDGTCVASTSLSYCERSQSIPVDGCDWKTFLSYGDLELIDMYCQLRSSCQLQGSCKRV QSSQNNASSPSHDFSSILSIDDTDSISMKSNVTDGRVSKGSTDNISATQNNTNDTFNSQSIPSDNSIVPN ISLFDVNTQNTTYSSSLIEANTTSVNHSASLADASIVKILSSDSIGNINSQVENYFNETIKHFAGKMSVN QTASNDLDLLETKRQDTTSNITSTAHLGGVQLNATLSAKDKSVAETRNSSNGLTSTYHNSDTGHISTTHI NNKGQISSTSVLDLGHVSTSFIDSNGAICTVYIDSNGNVSTSYNDGNGRVSTTHIDRKGQISTTHLDSDG HISISRIDSKGLVSTIRTDSKGRNSKRDDTSVEVGSYTYGDSIGHVEPKPSVRDNETASQSLELSFNPKD IFHNKAISVHKVACYLSFYMCLIQVIIVTLRLL
