# Supplementary material for: Proteomic analysis of Biomphalaria glabrata plasma proteins with binding affinity to those expressed by early developing larval Schistosoma mansoni
Source: PLoS Pathog. 2017 May 16;13(5):e1006081. doi: 10.1371/journal.ppat.1006081 (PMC5433772; doi:10.1371/journal.ppat.1006081)
Supplement: S7 Table — (PDF) [file ppat.1006081.s008.pdf]

S7 Table. PCR primer sequences used to amplify transcripts encoding FREP12, GREP, CREP2, and ADAM-TS from *Biomphalaria glabrata* whole body extracts.

**Primer**	**Forward (5'-3')**	**Reverse (5'-3')**	**Amplicon size (bp)**	**Tm (°C)**
Alpha-actinin	GGATTCGTCGTACTAGACCTTGGTT	TGCTTGACTTCTCCCCATTTGTT	897	53
GREP	CCAGAGTTCAGTTCTGTCAAAATG	GAGGCTTAGCTCATGAAGAACTAT	1835	53
CREP2	TCGGGAACTAGATCTAGGTTCAAG	GGTGTTCACACATGTAATGGTTAG	977	52
ADAM-TS	TACGTGACATACCCGGATCA	CAAGCCTTACGTACCTGTACTC	977	52
BgFREP12	CGGTGGATGGATCATCTTTCA	TGCACAGTTTAAGTGACTGTCT	358	52
